# Tumour boards and their quality of structures, processes, and team performance in multidisciplinary cancer care: a systematic review

**DOI:** 10.1186/s12913-026-14447-9

**Published:** 2026-03-31

**Authors:** Andrea Schweiger, Nicole Grössmann-Waniek, Guido Offermanns, Alexandra Kratki, Tarquin Mittermayr

**Affiliations:** 1 Department of Management, University of Klagenfurt, Universitätsstraße 65-67, Klagenfurt, 9020 Austria; 2 Karl Landsteiner Institute for Hospital Organisation, Auhofstraße 10, Vienna, 1130 Austria; 3 Austrian Institute for Health Technology Assessment GmbH, Josefstädterstrasse 39, Vienna, 1080 Austria

**Keywords:** Multidisciplinary team meeting, Oncology, Tumour boards, Patient management, Quality, Structures and processes, Team performance

## Abstract

**Background:**

In many healthcare systems, multidisciplinary team meetings (MDTs or tumour boards) play a central role in cancer care. MDTs give treatment recommendations based on the current medical knowledge of therapies and drugs. However, they require a high range of financial, human, and time resources, while the effectiveness of MDTs and their benefits for patients is still insufficiently scientifically evaluated. A comprehensive systematic review of the existing literature was conducted, summarising the available evidence on the effectiveness of MDTs. The aim of this systematic review was to analyse the structures and processes of MDTs, to identify factors that are crucial running a high-quality MDT regarding team performance, and their impact on patient management.

**Methods:**

We systematically reviewed the literature in PubMed, using different keywords regarding the effectiveness of multidisciplinary teamwork in MDTs. Studies published between 2011 and November 2025 concerning MDTs in clinical settings were considered. PICO elements were used as parameters to set up inclusion and exclusion criteria. The selection of articles followed the PRISMA 2020 statement. Three researchers independently screened and evaluated 371 full-text articles out of 3,430 abstracts through the Rayyan tool and Endnote. Data from 97 selected studies were extracted, including 85 original research articles and 12 systematic or scoping reviews. Relevant information was categorised according to author, study design, methods, objective, and conclusion.

**Results:**

Results show the pivotal role of MDTs in cancer care for improving quality, coordination, and evidence-based discussions in cancer care. Although validated key performance indicators for MDT quality exist, they are inconsistently implemented in routine practice due to structural, organisational, and resource-related constraints.

**Conclusion:**

Future studies should focus on assessing MDTs within specific tumour types to facilitate comparability and to better understand their impact on patient management outcomes. Implementational constraints should be faced by targeted organisational development, systematic quality assessment, leadership and communication training to further improve MDT performance, decision-making and quality of care.

**Supplementary Information:**

The online version contains supplementary material available at 10.1186/s12913-026-14447-9.

## Background

In cancer care tumour boards (multidisciplinary team meetings or MDTs) are considered an essential component of the diagnostic process by linking clinical information from different disciplines and serve as a central coordination point in the patient’s cancer journey [1]. They provide treatment recommendations based on available information regarding systemic and other therapies, while requiring a substantial range of financial, human, and time resources [2, 3]. Internationally, MDTs have become the gold standard practice for cancer patients, aiming to deliver high-quality cancer care and improve communication, coordination, decision-making, and survival [4–6]. Nevertheless, evidence suggests that MDTs do not always perform optimally in clinical decision-making and that not all recommendations are implemented [7–9]. For instance, the decision-making process in MDTs is characterised by the inconsistent attendance of core disciplines, making it challenging to exchange complete patient information. This may lead to repeated patient presentation until the MDT can reach a decision, or to recommendations being made based on incomplete information. In some cases, recommendations are subsequently modified outside the MDT meeting, meaning that the final decision is no longer multidisciplinary. Both scenarios can harm patients, leading to delayed treatment, suboptimal decisions, or additional resource use [8, 10, 11]. Validated self-assessment instruments are already in use internationally for assessing the quality of MDTs and enhancing their effectiveness. These instruments identify the key components of successful teamwork. They are used to evaluate decision-making processes and patterns of communication or leadership such as ATLAS (A Tumour Leadership Assessment instrument) [12], MDT-MODe (Multidisciplinary Tumour board – Metric of Decision Making) [13–15], MDT-QuIC (Multidisciplinary Tumour board - quality-improvement checklist) [16], MeDiC (Measure of Case-Discussion Complexity) [17], MDT-FIT (Multidisciplinary Team Feedback for Improving Teamwork) [18] and TEAM (Team Evaluation and Assessment Measure) [19]. Despite the availability of these instruments, robust evidence on MDT performance remains limited, largely due to methodological challenges, including the lack of appropriate comparison groups. Consequently, the relationship between MDT team performance and its impact on patient management is still insufficiently understood, despite the widespread implementation of MDTs in cancer care.

This systematic review aims to analyse and describe the key performance indicators of MDTs based on the structure-process-outcome model [20] with a focus on factors influencing the efficiency, functioning, and quality of MDTs. Beyond providing a comprehensive overview of the existing literature, this review seeks to synthesise evidence on MDT performance and identify approaches for refocusing and improving MDT meetings. The results will serve as a basis for developing more effective and sustainable models of MDT working.

## Methods

### Systematic literature search

A systematic review was conducted in PubMed using different keywords regarding the effectiveness of multidisciplinary teamwork in MDTs. A literature search was additionally conducted in three databases (Cochrane, Centre for Reviews and Dissemination (CRD), and Embase via Science Direct) to validate the results from PubMed. All relevant articles identified in other databases were also indexed in PubMed. Consequently, the systematic search was limited to PubMed. The literature search was updated three times (search dates: 17 June 2024, 24 March 2025, and 27 November 2025) during data extraction to identify any newly published articles. All search updates were conducted using the same predefined search strategy, screening processes and eligibility criteria to ensure methodological consistency. The last update on 27 November 2025, defines the evidence base of the review. A full description of the executed search is provided in Additional file 1 – Supplement A. Further articles were identified through citation tracking in Google Scholar, and additional relevant studies were identified using PubMed’s “related articles” function. During abstract screening, studies published between 2011 and 27 November 2025 were considered and assessed according to predefined PICO (Population, Intervention, Comparison, Outcome)-based inclusion and exclusion criteria (Tables 1 and 2).

**Table 1:** Inclusion criteria set by PICO-elements

Population	Intervention	Control	Outcomes	Study design	Publication year
Tumour boards (MDTs)	Multidisciplinary tumour boards Multidisciplinary team meetings (MDTs) in cancer care	Team performance Team function	**Quality and processes** Characteristics of an effective tumour board (documentation, communication, coordination, decision-making process, facilitators and barriers in team dynamics, effective teamwork in cancer care, team performance, adherence to recommendations) **Impact on patient management with team performance** **Implementation of virtual MDTs** **Resource requirements of MDTs**	Observational studies Longitudinal studies Cohort studies Mixed-methods study design Cross-sectional studies Systematic and scoping literature reviews Evaluation reports Feasibility studies	2011 and later

**Table 2:** Exclusion criteria set by PICO-elements

Population	Intervention	Control	Outcomes	Study design	Publication year
Individual board members Molecular or genetic tumour boards Rare diseases Geriatric tumour boards Paediatric tumour boards	Specific medical outcome Outpatient Primary care Rehabilitation Survival rate as outcome Perspectives of mandatory professions Perspectives of non-mandatory professions No aspects of teamwork	No team performance No team function	Medication Treatment effects Staging Survival rate Impact of MDTs on survival rate Impact on patient management without team functioning	Abstract Case reports Correspondence Conference review Editorial Narrative studies Short Communication Validation studies	2011 and later

**Fig. 1:**
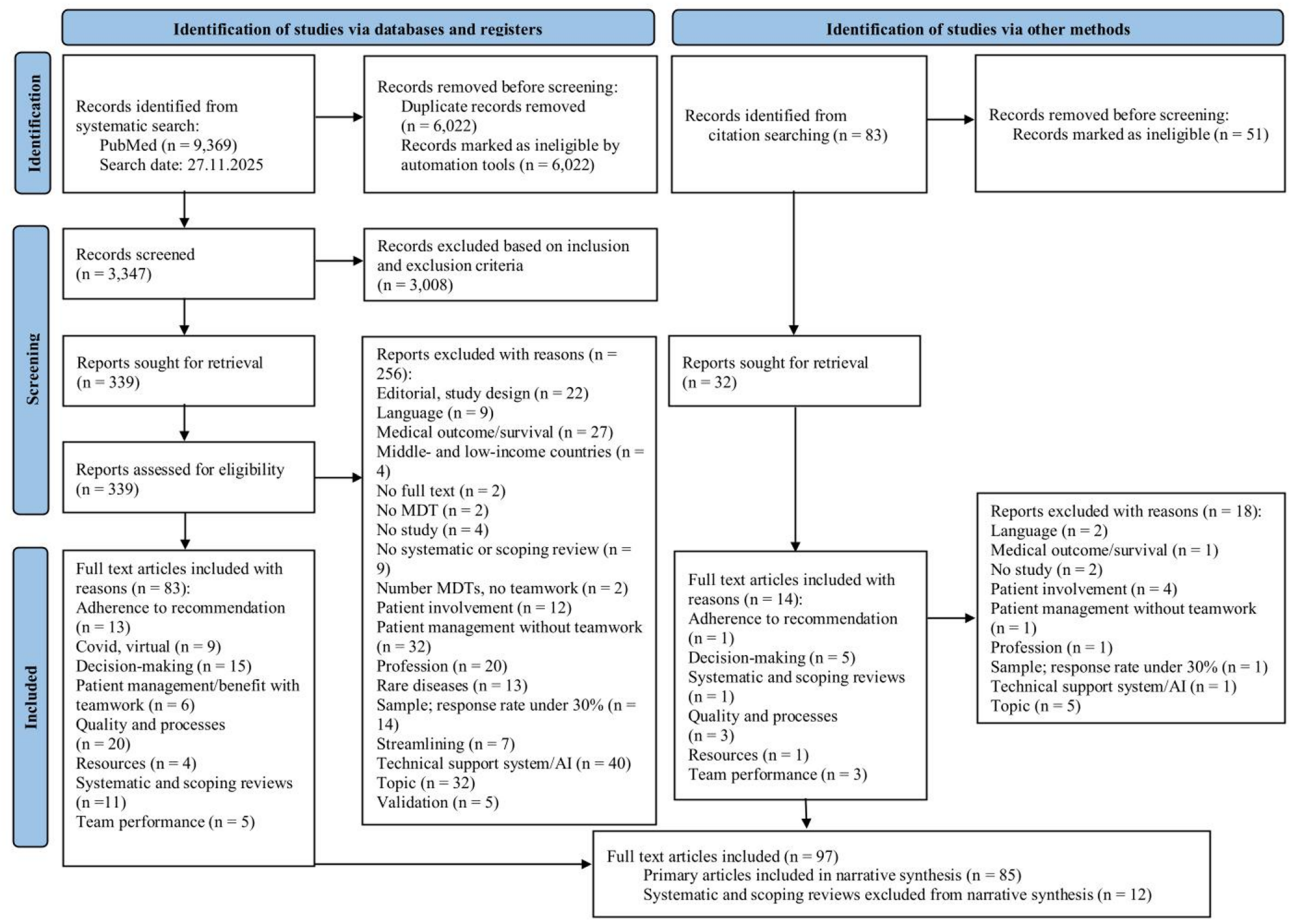
PRISMA flow diagram [22]

After deduplication, 3,430 records were screened at the abstract level. Abstract screening was performed by one reviewer and independently checked by a second reviewer using Rayyan and EndNote; discrepancies were resolved by consensus. A total of 371 articles were selected for full-text review. The study selection process is illustrated in the PRISMA (Preferred Reporting Items for Systematic Reviews and Meta-Analyses) 2020 flow diagram (Fig. 1).

Based on the full-text review, 97 studies met the inclusion criteria. Of these, 85 primary studies were included in the final narrative synthesis [3, 21], while 12 systematic or scoping reviews were excluded to avoid duplication of evidence (search date: 27 November 2025).

### Data extraction

Data extraction was conducted by one researcher (A.S.) and validated by other researchers (N.GW and A.K.). Owing to the heterogeneity of study designs, full quantitative data coding was not feasible. Instead, data were organised into predefined thematic categories. Following the literature review, factors for enhancing the quality of MDT processes were extracted. The included studies were summarised according to the previously defined research questions and categorised according to author, year, study design, objectives and setting, methods, results and conclusions. For a detailed overview of the study characteristics and included studies, see Tables S1–S7 (Additional file 2 – Supplement B).

### Quality appraisal

MMAT (Mixed Methods Appraisal Tool) was used to assess the methodological quality of the empirical studies, following the approach of Horlait et al. (2021) [3]. For each article, A.S. and A.K. assessed methodological quality independently, and discrepancies were resolved via consensus. The purpose of the quality appraisal was to identify whether any of the included studies warranted cautious interpretation (Additional file 3 – Supplement C). All the articles included were retained in the analysis, but some studies were labelled as being of low methodological quality, because of low non-response bias and low representativity of the target population. Overall, most findings may not be generalised to all MDTs or regions due to differences in team composition, resources or patient demographics and were therefore considered partially representative. In seven studies, representativity of the target population was given [23–29], whereas one study showed no representativity as it included only one MDT [30]. Although all the included studies report sample characteristics, a potential non-response bias remains a methodological limitation. Simply reporting the response rate does not allow for a conclusive assessment of whether, and to what extent, participants differ systematically from non-participants. In the context of MDTs, where diverse professional groups with varying time and institutional resources are involved, selective participation is likely. For example, professionals with a positive attitude towards tumour boards, or those from larger, better organised institutions, may be overrepresented. This potential bias could limit the generalisability of the findings and should be considered when interpreting the study results. As the quality appraisal indicated, the comparability of studies is hampered by different study designs, sample sizes (comparison between MDTs or analysis of case presentations), different tumour entities or settings. These characteristics provide background information and inclusion criteria but are not critical to answering the research questions and are therefore not explicitly described in the presentation of results.

## Results

The aim of this review was to analyse team processes and procedures in MDTs regarding so-called soft factors. These factors are mainly reflected in the results and conclusions of the included studies and are therefore difficult to compare across MDTs with differing interaction patterns, cultures, and team compositions. The results were grouped into the following key thematic areas relevant to effective teamwork in MDTs: Quality and processes, team performance, analysis and assessment of the decision-making process, adherence to recommendations, impact of MDT performance on patient management, implementation of virtual MDTs (facilitators and barriers) and resource requirements of MDTs.

### Data characteristics of included studies

A total of 97 articles met the inclusion criteria, of which 85 original research articles were included in the narrative synthesis. 12 systematic or scoping reviews were identified during screening. These were examined for contextual understanding and citation tracking but were not included in the narrative synthesis to avoid duplication of evidence [3, 21, 31–39]. Due to the heterogeneity of study designs, outcomes, and methodological approaches, a narrative synthesis of the evidence was conducted [3, 21].

Between 2011 and 2019, 36 articles were published, representing 42.4% of the overall sample. In contrast, 49 articles (57.6%) appeared during the shorter period from 2020 to 2025. Research from 2011 to 2019 primarily focused on the development of self-assessment tools for evaluating MDT performance and on identifying factors that contribute to effective MDT functioning. In recent years, the growing number of publications has emphasised the increasing scientific and clinical importance of MDTs. Particularly since COVID-19, research has focused on the effectiveness of virtual MDTs, using established knowledge of effective MDT structures, team processes and the adherence of recommendations.

The characteristics of the included studies are summarised in Table 3. Most studies were quantitative (*n* = 60), followed by qualitative (*n* = 13) and mixed-methods studies (*n* = 12). Studies were conducted across 16 countries, with the majority originating from the United Kingdom (*n* = 33) and the United States (*n* = 10). The most frequently assessed cancer types were gastrointestinal tumours (*n* = 37) and breast cancer (*n* = 34). Further methodological details and study characteristics are provided in Tables S1–S7 (Additional file 2 – Supplement B).

**Table 3:** Characteristics of included studies

Category	*n*
**A. Study design (** ***N*** ** = 85)**	
Quantitative	60
Qualitative	13
Mixed-methods	12
**B. Countries of origin (number of countries = 16)**	
United Kingdom	33
United States	10
Australia	8
Germany	8
The Netherlands	7
Sweden	4
France	2
Italy	2
Saudi Arabia	2
Spain	2
Switzerland	2
Austria	1
Belgium	1
Denmark and Norway	1
New Zealand	1
**C. Cancer types assessed (total mentions = 248)**	
Gastrointestinal tumours	37
Breast cancer	34
Thoracic tumours	26
Gynaecological tumours	24
Urological cancers	23
Head and neck tumours	22
Skin cancer	16
Liver and biliary tract tumours	15
Central nervous system tumours	14
Haematological malignancies	13
Soft tissue and bone sarcomas	13
Endocrine tumours	5
Genitourinary tumours	2
No specific entity	4

### Quality and processes

23 articles examining the quality and processes of MDTs were included [25, 40–61]. In analysing factors affecting the quality of MDTs, facilitators and barriers for an effective MDT were found. Most studies identified a heterogeneity in MDTs functioning regarding organisational structures and processes as well as team composition across entities, countries and hospital settings [41–43, 45, 51, 52, 56]. In an online survey conducted by Lamb et al. (2014) [46] to examine participants’ perspectives on existing MDT practices and to identify potential interventions to improve efficiency and productivity, 68% of respondents stated that participation in MDT meetings improves the effectiveness of patient care. Nevertheless, MDTs are time-consuming and their efficiency remains debated [23]. Addressing this, less complex cases could be discussed before the MDT and not presented to the MDT at all, and prioritisation according to case complexity, tumour entity or participant attendance could facilitate the implementation of MDTs [46]. Also several authors indicate that the quality of processes in MDTs could be improved through regular attendance by the core disciplines and the case manager [43, 44, 62], comprehensive and detailed documentation [60], structure of meetings [62], the role of team members and leadership [43, 52], predictable times for MDT preparation [51], a more structured way of working, prioritisation of complex cases [25], the use of checklists [52, 62] and accessibility and availability of patient-related information (clinical results, radiology, psychosocial status and comorbidities) [50, 51], which can be regarded as one of the decisive factors for a functioning MDT. In line with this, Gouliaev et al. (2024) demonstrated that the quality of patient-related information varies in different settings. Although they identified consistent and high-quality MDT clinical decision-making, patient-relevant factors such as comorbidities, preferences or psychosocial factors were not systematically considered. Findings showed high MDT-MODe scores (> 3) for medical history, radiology, pathology, and comorbidity, indicating comprehensive clinical and diagnostic data presentation. In contrast, significantly lower scores were recorded for psychosocial factors and the patient’s perspective, suggesting that these factors were not well represented in case discussions [48, 56].

The quality of discussions and the extent to which team members participated contributed to variations in recommendations. This suggests that active participation by different disciplines during MDTs, which is often influenced more by time constraints and workload than by conscious decisions, may limit the input of team members and ultimately influence treatment pathways. Khassan et al. (2023) found that at centres with a high caseload, resulting in little time for discussion, surgical team input was reduced which was associated with a lower rate of surgery for patients with ovarian cancer [44]. In contrast, enhancing team member engagement and leadership were shown to strengthen cancer care decisions [50]. Improvements should be made to each team’s specific context, focusing on adequate staffing, information accuracy, and improved workflow processes [60], but also streamlining of patients, auditing meeting decisions and prioritising complex cases which could be adopted for all entities [49].

Beyond team-level factors, organisational structures have been demonstrated to play a critical role in ensuring MDT effectiveness. Findings suggest major differences in the organisation of MDTs and the role of management should be improved [52]. In line with that, time pressure and inadequate organisational support were identified as key barriers to their optimal functioning [58] and changes in structures and procedures have been associated with an increase in perceived MDT quality [25, 50, 51, 63]. In this context, regarding preparation time and attendance Soukup et al. (2021) reported, that MDT meetings were not always multidisciplinary and clinical information was incomplete in a significant proportion of sessions, resulting in re-discussing the cases in question: In 8% of cases, all core disciplines were present, in 4% cases were presented inappropriately and in 8% recommendations were not communicated to the team [54]. Similar findings were reported by Zasada et al. (2023), who stated that incomplete patient information led to re-presentation in more than a third of cases. Cases of non-attendance or inappropriate presentation often required further discussion before a diagnosis could be confirmed [60]. Findings emphasise the need for more standardised processes and improved governance structures including clear leadership responsibilities, well-defined roles, pre-set agendas, sufficient preparation time, resource allocation and consistent attendance of core disciplines [42]. Furthermore, evidence suggests that electronic and standardised documentation systems had a positive influence on processes and outcomes by influencing communication and transparency during meetings and enables the MDT to be evaluated continuously [40, 42]. In addition, several studies described incomplete or inconsistent case documentation, resulting in repeated case discussions [54, 60]. These deficiencies seem to complicate the decision-making process and team performance and emphasise the importance of standardised documentation and information processes.

### Team performance

Eight articles regarding the team performance were included [64–71]. Another factor enabling MDTs to function effectively is team performance and the behaviour of the involved disciplines. For this reason, discussion and communication patterns have been included in MDT self-assessment instruments in the last 15 years [71]. Moreover tools for evaluating team performance have proven effective in improving team functioning [18, 68]. In this context, Lamb et al. (2013), among the first to consider team performance in MDTs, stated the development of team skills, a good relationship between MDT participants, organisational support, presentation of patient-centred information and recognising the central role of nursing as patient advocate as important factors for effective MDT functioning and for enabling patient-centred oncological care. Moreover, they underlined documentation of disagreements during discussions as necessary for transparent teamwork [71]. Additionally, Walraven et al. (2023) reported similar results to run efficient, competent and high-quality MDTs, deriving four key issues from 55 facilitators and 45 barriers such as organisational aspects (e.g., structure, time, preparation); responsibilities and requirements (e.g., chairperson, leadership, team composition); competences and team dynamics (e.g., communication, hierarchy, team climate) and meeting content (e.g., relevance of discussions, educational function). The authors recommended to include better IT use, formal training, and potential streamlining of cases to reduce time pressure [67]. In line with this, Soukup et al. (2020) reported a link between how complex a patient’s case is and how MDT members communicate. Results show that higher work pressure is linked to reduced communication and reduced positive interactions between team members and the more time spent on a case, the less communication and positive interactions were made. Team size and discipline were positively related to task-oriented communication and negative socio-emotional interactions. Therefore, the authors suggest that smaller, gender-balanced MDTs with the attendance of core disciplines and reduced time-workload pressure, time-on-task effects, and logistical issues are more conducive to enhance team performance in MDTs [65]. To underline these findings, Soukup et al. (2022) found administrative and process issues as the most frequent logistical challenges. Unavailable diagnostic results and inadequate patient details were also associated with reduced quality of decision-making [64].

As several studies identified similar key findings for team performance, a mixed-methods study was conducted by Soukup et al. (2023) to explore dynamics of group interaction and teamwork during MDT meetings. In the analysis of interactions and communication patterns, surgeons accounted for 47% speaking time; while nurse specialists and coordinators contributed the least (between 4% and 1%), with highly interactive discussions, each initiated sequence of interaction prompted more than one response on average, with surgeons and radiologists as most frequent responders. An overall increase in the second half of the meeting in verbal fragmentations of 52% such as incomplete sentences, pauses, pitch peaks, repetitions, vocalisation, interruption, chatter and laughter was observed, with some differences between MDTs (breast: 96%; colorectal 14%; gynaecologic: 18%). Overall, incomplete sentences and interruptions approximately doubled in frequency in the second half of the meeting, while chatter and laughter showed a 540% increase. Results emphasise the need to consider communication and interaction patterns, cognitive load in decision-making and hierarchical dynamics of MDTs [70]. Hitz et al. (2022) analysed team functioning across different tumour types and relevant information and presence of relevant cancer specialists, were consistent with no systematic differences in team working across different tumour types. Consistent with previous research, factors critical for effective decision-making and team performance were identified, showing minimal tumour-specific variation, emphasizing the universal importance of general structural elements such as leadership quality and team collaboration [69]. Other studies found different communication patterns in blood and bone marrow MDTs, where the quality of information was richer and more narrative-based including social, emotional, and caregiver concerns [66]. Soukup et al. (2020; 2023) observed similarities in communication patterns in breast/gynaecologic and skin MDTs, which focused on medical facts, and seemed declarative whereas communication in colorectal MDTs appeared more interrogative [65, 70].

### Analysis and assessment of the decision-making process

20 articles regarding the decision-making process were included [18, 62, 63, 72–88]. Across all studies analysed, the effectiveness of MDT decision-making depends not only on the quality and completeness of the information presented, but also on attendance, workload pressure [80] and preparation time, as well as communication styles and team performance [62, 75, 83, 86]. High-quality and comprehensive information facilitates recommendations, whereas incomplete or inconsistent data hinder the decision-making process [63, 74, 80, 87]. In line with this. Lamb et al. (2013) found in a prospective longitudinal study measuring the decision-making process (using MDT-MODe and MDT-QuIC) a positive correlation between the decision-making process, the information available and the quality of teamwork in the meetings. The availability of complete, patient-relevant data and the quality of teamwork lead to a steady improvement in the decision-making process and a reduction in the duration of meetings. These factors can be improved through simple interventions (including checklists, case structuring or feedbacks) [18, 73]. Soukup et al. (2016) obtained similar results in their observational studies. The authors found that the team’s ability to reach a recommendation is influenced by the quality of the information presented. Although psychosocial information is not presented regularly, its incorporation has been shown to enhance the team’s decision-making capacity. Conversely, contributions with information on comorbidities have been shown to slow the decision-making process, potentially signifying the complexity of the case and necessitating a more comprehensive discussion [81, 82]. In line with this, Wihl et al. (2021) demonstrated that during case presentations and discussions in MDTs, information on comorbidities was presented in only half of the cases, while non-medical information was presented in less than 10% of cases [63]. These findings highlight the need for standardised documentation, including the management of different opinions in the decision-making process [63, 72]. In an exploratory German observational study by Hahlweg et al. (2017), the MDT-MODe instrument was adapted to assess the quality of the information presented and team processes. Findings revealed a correlation between the quality of the information presented and the recommendations made, with time pressure as a significant factor in the decision-making process. In contrast to Soukup et al. (2016) [82], Hahlweg et al. (2017) suggested that missing information about the patients’ psychosocial profiles or preferred treatment options did not seem to significantly influence the final treatment decision, but presenting such information would likely have facilitated and streamlined the decision-making process within the MDT [87]. Nonetheless, the decision-making process remains predominantly guided by clinical information, with psychosocial or patient-related mostly underrepresented [63, 75, 77, 84, 87, 88]. The results found are consistent with other studies, including an Austrian adaptation of the MDT-MODe, and provide impetus for the reorganisation of MDTs [72, 74, 76]. In a prospective observational study analysing processes and interactions in team decision-making in MDTs, Soukup et al. 2020 demonstrated through the use of MDT-MODe and MeDiC, among other tools, that certain aspects of task-oriented communication (e.g., questioning, answering), external circumstances such as clinical or logistical complexity, and internal circumstances such as a larger team or a balanced gender distribution promote the decision-making process. Negative socio-emotional interactions, higher time and work pressure, the duration of the case discussion, or logistical complexity were identified as factors that hindered decision-making [80].

### Adherence to recommendations

14 articles were included [89–102]. In addition to the structures and processes necessary for an efficient decision-making process, compliance with recommendations or reasons for non-adherence must also be documented. Most recommendations (between 64% and 96%) are implemented in practice, highlighting the significant impact of MDTs on treatment planning and patient management [99–101]. In a retrospective comparative study by Hollunder et al. (2018), reasons for deviations from the multidisciplinary recommendation such as further examinations, tumour stage, patient preference, death, subsequent medical decision, insufficient documentation were cited [95]. Particularly latest studies from 2025 found patient preference as the most common reason for non-adherence [99–102]. Furthermore, Ernst et al. (2025) indicated, in line with findings by Vinod et al. (2021), that 58.8% of non-adherent recommendations were linked to a lack of documentation [98, 99]. Clinical status and additional information such as age, postmenopausal status, comorbidities or drug abuse were also found to be associated with changes in treatment plans [99, 100, 102]. In this context, Bortot et al. (2022) suggest more holistic, geriatric, and psychosocial assessments to ensure that decisions are concordant with patients’ real-world conditions [92]. Cao et al. (2023) further stated that the majority of non-adherence particularly regarding the choice between curative and palliative therapies in advanced stages of cancer [94]. It should be noted that Kandemir et al. (2025) also examined the impact on survival endpoints. Their results showed a positive effect of adherence on disease-free survival; a positive effect on overall survival was only observed in colorectal cancer [100, 101]. Overall, increased efficiency through consideration of patient preferences, improved presentation of patient-information, documentation and structuring of meetings could be achieved by using an quality assessment tool [96]. These findings underscore the need for routine evaluation of consistency between the MDT recommendation and the treatment implemented, which could serve as an important indicator of the effectiveness and quality of MDTs and patient outcomes [90, 98, 100, 101].

### Impact of MDT performance on patient management

Six studies regarding the impact of MDT performance on patient management were included [23, 26, 29, 103–105]. Although several studies have reported that team performance affects MDT functioning, only a few have investigated its impact on patient management [29, 104]. Findings of Mori et al. (2018) showed that 97% of participants perceive a moderate to significant impact of MDTs on patient care. After case discussion, diagnosis changed in 18–29% and treatment plans in 20–52% of cases, due to previously unconsidered options conducted by team members or to the promotion of adherence to guidelines. In line with this, Brauer et al. (2017) identified changes in the treatment plan in 25.1% of cases, mostly due to additional diagnostic investigations [23]. Similarly, El Saghir et al. (2015) reported changes to therapy for breast and colorectal cancer patients, in approximately 44–50% of cases, with adjustments to treatment or surgery occurring in 12–50% of cases. Notably, management changes occurred more frequently when cases were presented by physicians with less than 15 years of professional experience [103]. Another reason for adjusting the treatment plan was reported by Francisse et al. (2023), who found that treatment was modified in 72.2% of cases and diagnostic changes occurred in 17.6% of cases, primarily due to radiological re-evaluation, underlining the critical role of radiology in MDT decision-making processes [26]. Gandamihardja et al. (2019) indicated that a management decision or outcome was recorded in 92.2% of cases with surgical contributions dominating and nursing staff being significantly less involved, particularly regarding psychosocial and patient-centred aspects. These findings are consistent with other studies, regarding the communication patterns and decision-making process [44, 70, 71]. However, Petrella et al. (2021) observed only a modest impact on lung cancer, with MDT discussions leading to treatment changes in 10.6% of cases. Nevertheless, the authors suggest that the impact of MDTs on patient management is higher than their impact on survival rates [105].

### Implementation of virtual MDTs (facilitators and barriers)

Nine studies regarding the facilitators and barriers of virtual MDTs were included [24, 28, 30, 106–111]. Since the COVID-19 pandemic, MDTs faced additional challenges as meetings were transitioned from in-person to virtual formats. The virtual format led to a reassessment of their efficiency and effectiveness and the new demands on team members. Similar to in-person meetings, key factors for successful virtual meetings were identified in recent studies, including consistent leadership and organisation, active participation from all attendees, and adequate technological infrastructure [24, 28]. Virtual meetings were perceived as similarly effective [109] with positive effects on flexibility, improved attendance of core disciplines, timely diagnosis, multidisciplinarity [108, 109] and the remote participation of experts [106]. In line with this, Salami et al. (2025) reported a higher proportion of comprehensive recommendations in virtual MDTs (91.7% vs. 64.7%) and shorter evaluation times (23 vs. 39 days) [108]. Despite several positive aspects, virtual MDT meetings have been associated with multiple challenges, including reduced audibility, overlapping contributions, difficulties identifying speakers and limited opportunities for informal professional interaction [28, 111]. Mohamedbhai et al. (2021) reported that most clinicians perceived decision-making to be unchanged in virtual MDT meetings (70.1%), and 84.5% rated the available technology resources as satisfactory. In contrast, a majority of MDT members reported declines in engagement (43.9%), team working (69.1%), and educational value (47.7%) following the transition to virtual meetings [107]. Other difficulties arose from IT-related issues [110] and interruptions, which led to a delay in discussions [111]. Overall, virtual meetings face challenges like in-person meetings and may even require stricter structures, active participation, and positive team performance.

### Resource requirements of MDTs

Five studies regarding the resource requirements of MDTs were included [27, 112–115]. While several studies reported high resource requirements, only five studies have measured MDT-related costs over the past 14 years, resulting in limited available evidence. Only one scoping review has provided a comprehensive overview highlighting substantial heterogeneity and a lack of standardised reporting on costs and resource components with significant discrepancies required by tumour types and MDTs [33]. Attempts to assess resource expenditure were made [112, 114] evaluating the economic costs of MDTs based on the attendance of core disciplines and the associated preparation times, as well as the analysis of decision outcomes. Within the included studies, country-specific estimates illustrate the variability in resource requirements in MDTs. In Sweden, physicians need an average of 4.1 h per board for preparation, participation, and follow-up, with average costs per case discussion is €212 [112], while in the UK an initial patient presentation is up to £415 (€482) [114], with 84% of the total costs attributable to the time spent by physicians [112]. Ali et al. (2021) analysed the financial costs of MDTs based on meeting duration, salaries of core disciplines, and overhead costs. The mean total cost was £3,963.68, ranging widely from £946.12 to £9,353.94 across MDTs. The mean cost per patient discussed was £132.68, with a range from £31.67 to £313.10 [113]. In line with this, Mullan et al. (2014) evaluated initial patient presentations and the relationship between the time required per case, the number of discussants, and the type of case and it became clear that the longest discussions were held for patients in later stages of the disease. Fewer participants were involved in discussions about patients in earlier stages of the disease, and these were only discussed briefly to allow more time for more complex cases. This suggests that more complex cases also require increased resources [27]. Sassé et al. (2025) reported that MDTs for metastatic breast cancer generated an estimated weekly revenue of $571.18, while the cost of MDTs was estimated at $1,584.63 per week. This emphasises that the costs exceed the revenues generated, suggesting a substantial resource expenditure for the implementation of MDTs [115].

## Discussion

This systematic review emphasises that MDTs are widely established as a cornerstone of cancer care, playing a pivotal role in improving quality, coordination and evidence-based decision-making [24, 46, 69] and are associated with benefits for patient care and satisfaction [48]. Despite the availability of well-established performance indicators and management tools [18, 62, 72, 75], implementation gaps in routine practice often limit the effectiveness of MDTs and the performance of the team involved.

Aspects of teamwork within MDTs were examined in detail, revealing persistent areas for improvement. Across studies published between 2011 and 2025, fundamental challenges related to organisational structures, leadership and role clarity, attendance of core disciplines, decision-making processes, the quality of presented information, documentation, and adequate time for preparation and discussion have remained largely unchanged. These challenges were intensified largely by increasing demands on health professionals and the rise in people being diagnosed with cancer making the work of MDTs even more complex.

The findings of the systematic review indicate that, in recent years, research has shifted towards a more in-depth assessment of team performance and communication patterns using validated self-assessment tools. In addition, due to COVID-19, virtual boards have been introduced, offering more flexibility and the ability to bring in experts at short notice. However, this new format also brought new challenges to which MDTs must adapt. Studies primarily highlighted reduced social interaction as a disadvantage of virtual boards, emphasising the importance of interpersonal and non-verbal communication for team performance and mutual understanding within MDTs.

Our results further indicate that the effectiveness of MDT decision-making is influenced not only by the quality and completeness of the information presented, but also by attendance, workload pressure, preparation time, communication styles, and overall team performance. High-quality and comprehensive information facilitates recommendations, whereas incomplete or inconsistent data hinder the decision-making process. Studies show that variable attendance among core disciplines restricts the discussion of complete patient information. This frequently results in patients being postponed or in a recommendation based on incomplete information, which must be changed after the meeting and is no longer multidisciplinary or guideline-adherent. Both options are detrimental to patients may leading to delayed treatment or suboptimal decisions, and additional resources which could be avoided through clearly defined responsibilities and implementation processes [65, 72, 73, 83].

The level of evidence supporting the recommendation must also be evaluated. Shah et al. (2016) found that around 80% of recommendations are based on the highest level of evidence, which could have a significant impact on further treatment [79]. Although most MDT recommendations are implemented, deviations can occur due to patient preferences, death, tumour characteristics or inadequate documentation [89–91, 95, 97, 98]. These findings emphasise the importance of systematic documentation, comprehensive patient assessment and concise case presentation. Routine evaluation of the alignment between MDT recommendations and actual treatment could be a key indicator for MDT’s effectiveness [90, 98].

MDT meetings contribute moderately to patient management. However, the extent to which they lead to changes in diagnosis or treatment plans appears to depend on the quality of the case presentation, the structure of the meeting processes and team performance. Well-organised MDTs with effective team members seem to have a positive impact on patient management [23, 103, 105]. Although several studies address overall survival after patients were discussed at an MDT, they do not explicitly measure the extent to which team performance and the associated factors influence this outcome [116–121]. Therefore, future research should explicitly focus on the relationship between MDT performance indicators (e.g., leadership, case preparation, role clarity, communication quality) and patient management outcomes.

During the pandemic, virtual MDTs were an alternative to in-person meetings, and facilitated the attendance of experts from remote areas [106]. In this context, hybrid meetings have since been considered as a strategy to combine flexibility with the benefits of personal meetings [28]. However, virtual and hybrid formats face challenges, including IT-related issues, limited non-verbal communication, and pre-existing difficulties inherent to in-person meetings, while structural and resource constraints continue to limit their overall effectiveness.

Only a limited number of studies have investigated the resource requirements of MDTs, which showed substantial variation in costs across different MDTs, depending on discussion time per patient, case complexity and team composition. Although studies with a different focus also address the issue of resource expenditure, they do not analyse it in depth [104].

The included studies represent a wide range of methodological approaches, including quantitative, qualitative, and mixed-methods designs. This heterogeneity limited direct comparison across studies and restricted the possibility of quantitative synthesis. Additionally, comparisons between studies are challenging, as their results vary widely in professional groups, settings, or healthcare systems. Further research is needed in this area to enable the economic benefits of MDTs to be measured and presented transparently in future.

In summary, MDTs make a significant contribution to multidisciplinary, evidence-based, and patient-centred cancer care. However, their effectiveness depends largely on structural and organisational conditions, sufficient resources, strong leadership, team performance, and continuous evaluation [47, 69, 76]. In this context, prioritising complex cases is also recommended, with the aim of being able to discuss serious cases sufficiently. Findings regarding the mental exhaustion of MDT participants highlight the need to streamline meetings, ensuring that no patient have to be discussed as “number 70” [65, 122]. Future development should focus on systematic quality assessment, leadership and communication training and the consideration of psycho-social aspects to further improve MDT performance, decision-making and quality of care.

## Conclusion

The multidisciplinary approach is a key strategy for managing complex cancer care. Its successful implementation requires organisational and cultural changes as well as effective teamwork and strong organisational support. Although key performance indicators for MDT quality are well established, their implementation is frequently hindered by structural, organisational, and resource-related barriers, limiting MDT effectiveness in routine practice. Standardised tools, such as checklists or documentation systems may be applied across entities, but a “one-size fits all” model [25] seems ineffective, as different tumour types require tailored approaches. The development of team-related competencies, including shared mental models, structured debriefing methods, and regular team feedback, together with sustained organisational support, is essential for effective MDT functioning and the delivery of patient-centred oncological care. Future studies should focus on assessing MDTs within specific tumour types and on MDT team performance indicators (e.g. leadership, case preparation, role clarity, communication quality) to enhance comparability and to better understand their impact on patient management outcomes.

### Limitations

This systematic review has several limitations. The heterogeneity of study designs, outcome measures, and healthcare settings limited the comparability of findings and restricted the synthesis to a narrative approach [3, 21]. Moreover, the comparability of included studies was limited due to sample sizes, and methodological approaches, including surveys of MDT participants and observational analyses of case presentations. This heterogeneity restricted the direct synthesis of findings across studies. To address these challenges, methodological quality was appraised using the MMAT tool. While all included studies were retained in the narrative synthesis, some required cautious interpretation due to limitations such as low response rates or limited representativeness of the target population.

The review deliberately focused on team performance, processes, and other “soft factors” influencing MDT quality. As a result, studies primarily addressing clinical outcomes, survival rates, patient involvement in MDT discussions, streamlining strategies, or the use of artificial intelligence and digital decision-support tools were excluded, as these topics represent distinct research areas beyond the scope of this review.

In addition, the restriction to studies published in English and indexed in PubMed may have resulted in language or publication bias.

Despite these limitations, this review provides a comprehensive synthesis of current evidence on structural and process-related factors affecting MDT performance and quality in cancer care.

## Supplementary Information

Below is the link to the electronic supplementary material.

Supplementary Material 1: Supplement A - Full description on the executed search

Supplementary Material 2: Supplement B - Data extraction of all 85 included papers

Supplementary Material 3: Supplement C - Quality appraisal (MMAT)

Supplementary Material 4: Supplement D - Included and excluded articles with reason

## Data Availability

The datasets used and/or analysed during the current study are available from the corresponding author on reasonable request.

## Declarations

### Author's note

AI-based tools (DeepL and ChatGPT) were used for language refinement, translation, and style improvement. Output was rigorously reviewed and revised by A.S. No generative AI was used to create scientific content, results, interpretations, or conclusions.

## Abbreviations

ATLAS: A Tumour Leadership Assessment instrument
MDT: Multidisciplinary Team Meetings
MDT-FIT: Multidisciplinary Team - Feedback for Improving Teamwork
MDT-MODe: Multidisciplinary Tumour board - Metric of Decision Making
MDT-QuIC: Multidisciplinary Tumour board - Quality-Improvement Checklist
MeDiC: Measure of Case-Discussion Complexity
MMAT: Mixed Methods Appraisal Tool
PICO: Population, Intervention, Comparison, Outcome
PRISMA: Preferred Reporting Items for Systematic reviews and Meta-Analyses
TEAM: Team Evaluation and Assessment Measure
