# Supplementary material for: Tumour boards and their quality of structures, processes, and team performance in multidisciplinary cancer care: a systematic review
Source: BMC Health Serv Res. 2026 Mar 31;26:676. doi: 10.1186/s12913-026-14447-9 (PMC13162496; doi:10.1186/s12913-026-14447-9)
Supplement: Supplementary file 2 — Supplementary Material 2: Supplement B - Data extraction of all 85 included papers [file 12913_2026_14447_MOESM2_ESM.docx]

**Table S1. Structures and processes affecting the quality in MDTs**

| **Author; year; study design; country; setting** | **Objectives; sample** | **Methods** | **Results** | **Conclusion** |
| --- | --- | --- | --- | --- |
| Ali et al. (2023) [24]  Quantitative study  Skin cancer  United Kingdom | Identification of current practices in MDTs  Secondary analysis of 3,563 quantitative responses from two national Cancer Research UK surveys (2017) focusing on 282 responses from MDT members  2017 | Two survey questionnaires: Likert-scale data on MDT practices and preferences in comparison across tumour groups | Key findings: ready imaging/pathology, adequate discussion time, clear leadership, pre-set agendas; attendance tracking, checklists, and triage tools; need for risk stratification and discussion of complex cases | Tumour-specific guidance is necessary |
| Alsuhaibani et al. (2018) [40]  Retrospective observational study  Gastrointestinal cancer  Saudi-Arabia | Improvement of documentation and communication in the MDT by using four Plan-Do-Study-Act cycles to develop, implement and evaluate the intervention  One MDT  2015–July 2016 | Implementation of a documentation tool for MDT performance measurement | Electronic and standardised documentation improves communication within the MDT and enables evaluation | Positive influence on processes and outcomes through standardised documentation |
| Belda-Ferre et al. (2022) [41]  Cross-sectional descriptive study  Urological cancer  Spain | Evaluating the functioning of MDTs in six hospitals  Committee coordinators, urologists, oncologists, radiation oncologists, radiologists, pathologists, and oncology and urology residents  Study period not available | Literature review and development of surveys with MDT coordinators and members | Deficiencies in protocol updates, meeting agendas, audits, and scientific production  MDTs provide significant benefits to patient care and satisfaction | Several improvements aim to enhance MDT effectiveness and patient care: regular updates to clinical protocols; structured meeting agendas; audits; scientific production; management support including protected time for MDT meetings |
| Brims et al. (2022) [42]  Cross-sectional quantitative study  Lung cancer  Australia | Identification and description of infrastructure, composition, and care practices in MDTs  Assessment of variations in resources and organisational structures influencing patient outcomes and adherence to guidelines  79 responses of 109 institutions  January–July 2021 | Online survey | No institution reported full recommended attendance of core disciplines  Missing disciplines: lung cancer nurse specialists, MDT coordinators, nuclear medicine support, and thoracic surgery representation | Findings emphasise the need for improved standardisation, resource allocation (including regular attendance of core disciplines) and national data collection to enhance lung cancer care quality |
| Casadio et al. (2022) [43]  Cross-sectional observational study  Cholangiocarcinoma  European multicentre study  United Kingdom | Identification of the current practice, minimum standards and opportunities for improvement of MDTs  Members of the European Network for the Study of Cholangiocarcinoma (ENS-CCA) and other medical centres with expertise in biliary tract cancers  February–May 2021 | Online survey | Key elements for a well-functioning MDT: a dedicated coordinator and provision of minimal clinical information prior meetings; 73.06% supported discussion of all new cases and treatments; 46.15% reported implementation in practice; guidelines were used in 50% of cases | Variation in current MDT practices  Existing consensus on the importance of core specialties and structured processes  Minimum standards improve quality of care and consistency  Representation of certain specialists essential for effective MDT functioning |
| Gouliaev et al. (2024) [56]  Cross-sectional observational study  Lung cancer  Denmark and Norway | Comparison of MDT functioning and decision-making processes in Denmark and Norway  13 Danish and 18 Norwegian institutions; respiratory physicians, radiologists, oncologists, thoracic surgeons, and nurse specialists  Fall 2022 and January–February 2024 | Mixed-methods study design  Online national surveys: team composition, meeting structure, documentation, and quality assessments  MDT-MODe: observation and assessment of six MDT meetings | Similar settings and good-quality MDT meetings across Denmark and Norway  All meetings chaired by respiratory physicians including all specialties | MDTs in both countries support consistent and high-quality clinical decision-making  Incorporation of patient perspectives and psychosocial factors could be improved |
| Harris et al. (2014) [55]  Qualitative proof of concept study  Cancer types: Breast, Colorectal, Gynaecological, Head and Neck, Hepatobiliary, Lung, Sarcoma, Skin, Upper Gastrointestinal, Urology  United Kingdom | Development of a structured observational assessment tool for peers, exploring the views of MDT members and observers about feasibility  64 team members of 20 MDTs (32 consultant physicians, 14 nursing, 15 administrative, and 3 allied health professionals) and 19 peer observers | Brief, semi-structured telephone interviews with MDT leaders and up to three other MDT members  The tool was based on 'the characteristics of an effective MDT' of the National Cancer Action Team (2010) [123] | The tool was easy to use, with suggested refinements  Most MDT members (73%) described the observational assessment and feedback as useful, providing valuable feedback and implications for practice  Limitations: observer effect altering 'normal' behaviour; concerns about validity based on a single observed meeting | Initial evidence for peer observation and feedback as a valuable method for assessing and improving the quality of MDTs |
| Khassan et al. (2023) [44]  Multi-centre observational study  Advanced ovarian cancer  United Kingdom | Assessment of multidisciplinary team behaviour and influence on treatment decisions  17 MDT meetings across five cancer centres; 145 cases  May–June 2022 | Survey: GO-MDT-MODe^[[1]](#footnote-1)^: assessment of case discussion quality and clinician participation; correlation with surgical treatment data from national audit records | Significant differences in structure, discussion time, and clinician participation  Centres with more engaged disciplines showed more high-quality discussions and surgical rates | Impact of team behaviour on treatment pathways for advanced ovarian cancer  Quality of discussions and team involvement may contribute to different recommendations (e.g. involvement of surgery) |
| Kočo et al.( 2022) [51]  Qualitative interview study  Breast cancer  The Netherlands | Organisation of MDTs and identifying areas for improvement  Three hospitals; 24 core disciplines; surgeons, oncologists, radiologists, pathologists, nurses etc.  January 2019–February 2021 | Semi-structured interviews regarding potential improvements in breast cancer MDTs; current MDT organisation and member´s role; preparation routine; time management | Preparation; structure; data availability; meeting discipline highlighted as essential factors for efficient MDT improvement | MDTs are important for diagnosis, treatment planning, communication, and education  Results seem to be applicable to other cancer types as well |
| Lamb et al. (2012) [45]  National survey analysis  Cancer types: Breast, Colorectal, Lung, Gynaecological, Head and Neck, Upper Gastrointestinal, Urological, Haematological  United Kingdom | Testing the appropriateness of a standard model for MDTs for different entities  Secondary data analysis from a national UK survey (2009)  1,141 MDT members  Study period not available | Online survey  Key domains of MDT functioning: meeting structure, logistics, decision-making, and governance | Variability in meeting preparation and organisation; case selection and late additions; clinical decision-making (haematology and lung MDTs)  Haematology MDTs: differences in perceptions of MDT impact on timeliness, patient choice, and survival  Lung and colorectal MDTs: variability in logistics and palliative care involvement | Adaptation of effective MDT core principles to different tumour types  Potential benefits for clinical decision-making, preparation, and meeting structure from tailored approaches per entity  Tumour-specific adaptations are recommended to enhance MDT effectiveness |
| Lamb et al. (2014) [46]  Prospective cross-sectional study  Urological cancer  United Kingdom | Investigation of participants' perspectives on existing MDT practices and interventions to improve the efficiency and productivity of MDT meetings  173 MDT attendees at the British Uro-oncology Group (BUG) 2011 Annual Meeting and the Royal Society of Medicine (RSM) 2012 Meeting  2011–2012 | Online survey | 68% reported improvements in clinical decisions, planning, investigations, discussions, referrals, and record-keeping  Minor cases: treatment plan decided before MDT  Prioritisation based on case complexity, tumour type, or number of participants | MDT usefulness recognized, but opportunities for improvement |
| Ottevanger et al. (2013) [52]  Observational study  Cancer types: Brain, Gynaecological, Lung, Urological, Head and Neck, Gastrointestinal, Breast, Haematological, Sarcoma, Children’s Oncology, General Oncology  The Netherlands | Development of a guideline for effective MDTs  Literature review; expert interviews with 15 MDT managers; 18 MDT sessions in 7 hospitals  January 2010–April 2011 | Measurement of guideline-compliance | Key topics: organisation; participation; responsibilities and roles; meeting procedures; documentation and recommendations | Major differences in MDT organisation  Role of management should be improved  Better information on quality criteria for MDTs |
| Polomeni et al. (2023) [57]  National mixed-methods study  Haematological cancer  France | Exploration of haematology MDTs and participants’ perceptions to identify factors affecting decision-making and effectiveness  Quantitative study with 205 participants; qualitative study with 22 health professionals  Study period not available | Online survey and semi-directive phone interviews regarding decision-making, benefits and inconveniences | Unique characteristics of haematology  Barriers: lack of time; compulsory discussion of all cases including straightforward ones; poor team dynamics; weak institutional recognition  Benefits: shared responsibility; knowledge exchange; training; enhanced coordination; improved guideline adherence | Performance hindered by organisational and interpersonal issues; better preparation; prioritisation of complex cases; inclusion of psychosocial data; leadership training to improve decision-making |
| Rankin et al. (2018) [47]  Quantitative study  Cancer types: Urological, Colorectal, Breast, Haematology, Lung, Gastrointestinal / Upper GI, General Oncology, Radiotherapy / Palliative Care, Gynaecological, Head and Neck, Thyroid, Neurological, Liver, Pituitary and Pelvic Exenteration, Sarcoma  Australia | Investigation of team functions; role of MDTs and use of evidence; differences and similarities  37 MDTs; 40 surveys returned  January 2016 | 40-item online survey: organisation; resources and documentation; evidence and guidelines; patient involvement; strengths and weaknesses of MDTs | Decisions based on consensus (92%), guidelines (57%) or other evidence-based sources (33%)  Weaknesses: availability of data and reflection of evidence-based cases  Strengths: collaboration and discussion | Focus on joint treatment decisions (consensus); guidelines and evidence-based-medicine |
| Robinson et al. (2017) [53]  Qualitative study  Cancer types: Breast, Upper Gastrointestinal Tract, Lower Gastrointestinal Tract, Lung, Melanoma, Gynaecological, Metastatic Breast  Australia | Identification of facilitators and barriers to engaging MDTs in translational research and quality improvement  Observations of 43 MDT meetings and 18 interviews; two hospitals  2013 | Board observations and semi-structured interviews | Key factors: access to high-quality performance data; active team leaders; embedded implementation scientists and dedicated quality improvement meetings | Quality improvement and translational research potential; MDT progression from case-based decisions to population-level improvements |
| Ruiz-Casado et al. (2018) [58]  Observational Study  Cancer Types: Gynaecological, Urological, Dermatological  Spain | Analysis of strengths and potential for improvement of MDTs  49 health professionals; observations; qualitative evaluation of five focus groups with 31 MDT participants  May–June 2021 | Online questionnaire (11 items)  Topics in focus groups: organisational factors; structures; decision-making process; strengths and weaknesses | Highest rating for benefits of MDTs for patients; difficulties are lack of time and lack of support from the organisation | Perceived benefits of MDTs among doctors; lack of organisational support affecting MDT functioning, quality improvement, and implementation |
| Seretis et al. (2014) [48]  Prospective observational study  Colorectal cancer  United Kingdom | Quality assessment of MDT meetings  64 case presentations  Study period not available | Online survey: cMDT-MODe^[[2]](#footnote-2)^ | Little knowledge of patient-relevant factors (comorbidities, preferences or psychosocial factors) | Detailed patient information not directly influencing final MDT recommendations |
| Soukup et al. (2021) [54]  Cross-sectional prospective observational study  Cancer types: Breast, Colorectal and Gynaecological  United Kingdom | Development of a new method to evaluate the decision-making process in MDTs  30 weekly sessions in three MDTs over twelve weeks; 822 case presentations; 44 MDT participants; surgery; oncology; cancer nurses; radiology, pathology, MDT coordination  September 2015–July 2016 | Analysis of 24 cases (of 822) focusing on MDT adherence to decision-making stages and alignment of multidisciplinary contributions with clinical guidelines | MDT composition varied: five core disciplines present in 8% of cases; four in 38%; two (25%) to three (29%) disciplines in 54% of case presentations; surgery (83%) and oncology (8%) involved across all decision-making stages; 4% inappropriate case presentation; 8% failure to communicate recommendations to the team | MDT meetings not always multidisciplinary  More 50% of case discussions not guideline-adherent |
| Soukup et al. (2023) [59]  Mixed-methods observational study  Cancer types: Breast, Colorectal, Gynaecological, Head and Neck, Lung, Skin, Upper GI, Urological, Other  United Kingdom | Analysis of the perception of change since COVID-19 considering pre-existing challenges in MDTs  423 MDT members  September 2020–August 2021 | Qualitative and quantitative analysis: MDT meeting organisation and logistics; access to MDT meetings; case discussions at MDT meetings and patient participation | IT-related issues: slower meetings, longer lists and delays with improvement of IT infrastructure  Positive outcomes in meeting organisation and logistics and no significant changes in access to MDT meetings; case discussions at MDT meetings and patient participation | Preferred hybrid working but challenges regarding the IT-infrastructure exist |
| Taylor et al. (2012) [61]  Mixed-methods descriptive study  Bowel cancer  United Kingdom | Development and testing of quality criteria for an observational assessment of MDT performance based on health professionals’ views on effective MDT characteristics  Ten MDTs  Study period not available | MDT-OARS^[[3]](#footnote-3)^: 15 aspects of MDT working covering four domains observed: team factors (attendance, chairing, teamworking); infrastructure for meetings (facilities, equipment); meeting organisation and logistics; patient-centred clinical decision-making (patient-centredness, clarity of recommendations) | Observational assessment perceived as acceptable and feasible; quality diversity between teams | Feasibility of measuring complex aspects of team behaviour and activities (e.g. leadership, teamworking and decision-making); Tool to assess MDT performance |
| Warner et al. (2021) [49]  Secondary observational study  Cancer types: Urological, Breast, Colorectal, Lung  United Kingdom | Perception of urology MDT members on meeting improvements in comparison with other entities  429 urology health professionals; respondents from other disciplines: breast cancer (503); colorectal cancer (431) and lung cancer (405)  2017 | Two online surveys by Cancer Research UK | Potentials for improvement over all entities: time for meeting preparation; streamlining of patients; auditing meeting decisions and prioritising complex cases | Results for urology suggest only Discussion of complex cases; other patients should be treated according to standardised protocols |
| Wihl et al. (2021) [50]  Observational study  Cancer types: Brain, Soft Tissue Sarcomas, Hepatobiliary  Sweden | Analysis of information sharing and contributions to case discussions to define key performance indicators  Three MDTs; 349 case discussion; 32 meetings | MDT-MODe  Quality of case presentation and team members’ contributions to case discussions | Radiology information most often presented, followed by case history and pathology; patient-related information less frequently shared; Discussion of contributions predominantly by chair, surgeons, and oncologists; improvement of case presentations through strong leadership | Enhancing team engagement and leadership could strengthen MDT decision-making |
| Zasada et al. (2023) [60]  Mixed-methods observational study  Lung cancer  United Kingdom | Investigation of the efficiency of MDTs and identify barriers for improvement  Eight MDTs; 56 health professionals; 96 meetings  April–June 2021 | MDT-QuIC  Semi-structured interviews on members’ experiences with MDT organisation and functioning, including challenges related to staffing, information availability, and workflow processes | 21% of meetings with full participation; 37% of patient cases required re-discussion before diagnosis confirmation | Variability in team working models and issues (e.g. incomplete clinical information and workforce shortages)  Team-specific improvements: staffing; information accuracy; workflow processes |

**Table S2. Adherence to recommendations**

| **Author; year; study design; country; setting** | **Objectives; sample** | **Methods** | **Results** | **Conclusion** |
| --- | --- | --- | --- | --- |
| AlFarhan et al. (2018) [89]  Prospective cohort study  Gastrointestinal cancer  Saudi Arabia | Evaluating the consistency of recommendations with international guidelines; adherence of physicians involved in patient care; impact on the management of patients  104 case discussions  January–June 2016 | Data collected weekly: adherence to guidelines, and management changes | 97% of recommendations aligned with guidelines (87% implemented within three months)  Management plans changed in 36% of cases | MDTs improved adherence to clinical guidelines and influenced patient management decisions; high physician compliance with recommendations |
| Alkasbi et al. (2021) [90]  Retrospective descriptive study  Head and neck cancer  France | Assessment of compliance with recommendations and documentation of non-compliance  January–December 2018 | Comparison of MDT recommendation with current treatment; documentation of deviations from the recommendation | Recommendations were mostly implemented (91.6%), with deviations due to side effects, patient refusal, doctor's decision, or death | Need to evaluate matches between the recommendation in the MDT and the treatment received |
| Basta et al (2016) [91]  Observational study  Gastrointestinal cancer  The Netherlands | Evaluation of correct diagnoses recommended and implementation of decisions  74 MDT meetings; 691 case discussions  December 2012–March 2013 and September–December 2013 | Validation of diagnoses through pathology or clinical follow-up; Check of implementation through electronic medical records | 93.5% of diagnoses proven correct; 22% of referral diagnoses revised by MDT; 87.8% of diagnoses matched pathological results; 94.4% of MDT decisions adopted | Attendance of case manager significantly improved diagnostic accuracy  Implementation of Nearly all MDT decisions |
| Brink et al. (2024) [93]  Observational cohort study  Renal cell carcinoma  The Netherlands | Evaluation of shared decision making and adherence to MDT recommendations  2,651 patient cases in a national network over five years  2017–2022 | Data collection: patient and tumour characteristics; treatment recommendations; adherence to MDT advice; use of shared decision-making; clinical trial inclusion rates | 96% adherence to recommendations  Multiple treatment options enabling shared decision-making (30% of cases) | Dutch network MDT enables shared decision-making by offering diverse treatment options and trial access |
| Bortot et al. (2022) [92]  Retrospective observational cohort study  Breast cancer  Italy | Concordance between MDT treatment recommendations and final therapeutic choices  291 patient cases  January 2017–June 2018 | Extraction of data on demographics, pathology, and treatments  Uni- and multivariate logistic regression | 15.8% of recommendations not applied  Main reasons: clinical decision (87%); patient preference (13%) or common changes in therapy | Discordance associated with clinical status (e.g. age, postmenopausal status, comorbidities, drug abuse) and may lead to treatment changes; recommendation for holistic, geriatric, and psychosocial assessments to align decisions with patients’ real-world conditions |
| Cao et al. (2023) [94]  Retrospective cohort study  Hepatocellular carcinoma  United States | Analysis of adherence to recommendations and reasons for non-adherence  225 patients  2013–2016 and 1 February 2022 | Examination of all patient cases between February 2013 and February 2016; survival data were collected through 1 February, 2022 | Adherence to treatment recommendations in 85.3% of patients; non-adherence mainly in BCLC Stage A, especially for curative vs. palliative therapy choices | Adherence to expert recommendations in the treatment of liver cancer; areas for improvement to enhance patient outcomes |
| Ernst et al. (2025) [99]  Retrospective single centre observational study  Cancer types: Dermatological, Hereditary, Gastroenterological, Breast, Gynaecological, Neuroendocrine and Thyroid, Thoracic, Head and Neck, Sarcomas, Musculoskeletal, Neuro-oncological, Urological  Germany | Assessment of adherence to MDT recommendations and factors hindering full implementation; evaluation of barriers (documentation, follow-up, patient preferences)  7,152 patients in ten MDTs; 13,050 recommendations  2014–2016 | Retrospective review of electronic medical records: total implementation; partial implementation; deviance; not assessable  Subcategories: patient wish; doctor decision/modified results; missing documentation; missing follow-up; patient death; other | Key gaps: incomplete documentation; missing follow-up, insufficient integration of patient preferences  Overall implementation 84.2%; total implementation 68.1%; partial implementation 16.1%; deviance 8.6%; not assessable 7.3% | High adherence to tumour board decisions  Potential improvements via digital tools, structured reporting, and patient-centred processes  Procedural improvements may enhance overall effectiveness of multidisciplinary cancer care |
| Gennheimer et al. (2025) [102]  Retrospective quantitative observational study  Multiple myeloma  Switzerland | Adherence to MDT recommendations assessed for diagnostic procedures, therapies, and clinical trial enrolment  218 patients  2023 | Retrospective review of electronic medical records | Implementation of 86% of recommendations (84% were followed with complete adherence and 2% incompletely) with diagnostics or therapy clinically justified  Main reasons for non-adherence: patient decision; lack of cost coverage and relevant changes in the clinical scenario, including patient’s death  In total 36% of recommendations included clinical trial enrolment | High adherence to MDT recommendations for diagnostics and therapy; only a third of clinical trial recommendations were implemented, mostly due to patient decisions |
| Hollunder et al. (2018) [95]  Retrospective comparative study  Cancer types: Neurological, Head and Neck, Sarcoma  Germany | Benefits of MDTs and increase in efficiency  Three MDTs  June 2014–December 2016 | Analysis of 2,450 case reports  Survey of compliance with recommendations and reasons for non-compliance | Most recommendations were implemented.  Reasons for non-implementation: patient request; death; medical decision or not evident from documentation | Increased efficiency through consideration of patient preferences, improved presentation of patient-information, documentation and structuring of meetings |
| Kandemir et al. (2025) [100]  Retrospective observational study  Breast cancer  Germany | Assessment of adherence to recommendation and reasons for non-adherence; evaluation of disease-free survival in relation to adherence  1,563 patients in two breast cancer centres  2014–2018 | Retrospective review of medical records of implementation of all MDT recommendations; investigation of reasons for non-adherence | Overall adherence 89.6%; patient preference as main reason for non-adherence (75.3%); chemotherapy rejection most common (36.9%) of non-adherent cases | High adherence rate (89.6%) to MDT recommendations; patient preference as key factor in non-adherence (75.3%) |
| Kandemir et al. (2025) [124]  Retrospective observational study  Colorectal cancer  Germany | Impact of adherence to recommendations on survival  458 patient cases  2014–2018 with a follow up until 12 December 2023 | Retrospective review of medical records of the implementation of all MDT recommendations; investigation of reasons for non-adherence | Overall adherence 88.6%; patient preference as main reason for non-adherence (44.2% of non-adherent patients) | Importance of adherence to MDT recommendations with a strong association with disease-free-survival and overall survival |
| Krause et al. (2023) [96]  Observational study  Gastrointestinal cancer  Germany | Understanding the consistency of adherence to guidelines and recommendations and implementation in clinical practice  732 case discussions  January–December 2020 | Therapy recommendations assessed for their adherence to guidelines and recommendations for each patient case; causes of deviation explored | Treatment recommendations fully implemented in 64% of cases  21% deviations mainly due to physician differences, patient requests, comorbidities, organisational issues, therapy complications, patient death, or tumour factors | Consideration of patient characteristics in depth, assessment of guideline deviations and adherence to recommendations by an objective quality assessment tool could become a quality criterion for MDTs |
| Rajan et al. (2013) [97]  Observational study  Breast cancer  United Kingdom | Evaluation of decision-making  3,230 MDT decisions relating to 705 patients  July 2009–June 2011 | Comparison of MDT decisions with subsequent patient management | 91.5% concordant decisions and 4.5% discordant. 82.2% of discordant decisions justifiable (41.8% patient choice, 37.0% new information and 3.4% due to MDT error) | High implementation of MDT decisions; management alteration most often due to patient choice or additional information after the meeting |
| Vinod et. al. (2021) [98]  Audit of oncology records  Cancer types: Lung, Breast, Upper Gastrointestinal, Gynaecological, Colorectal, Genitourinary, Skin, Head and Neck, Neurological  Australia | Evaluation of MDT recommendations translated into clinical practice  835 case discussions; nine MDTs  February–July 2017 | Information collected: patient and tumour factors and recommendations  Up to one-year reasons discordant with recommendations identified | Full adherence between 67.9% in the genitourinary MDT and 90.4% in the neuro-oncology MDT  Reasons for non-adherence: patient or guardian decisions; 16.7% showed a lack of documentation of recommendations | Differences in implementing MDT recommendations into clinical practice by tumour site  Routine evaluation of implementation as a quality indicator for MDTs |

**Table S3. Team performance**

| **Author; year; study design; country; setting** | **Objectives; Sample** | **Methods** | **Results** | **Conclusion** |
| --- | --- | --- | --- | --- |
| Evans et al. (2019) [68]  Mixed-methods study  Cancer types: Colorectal, Gynaecological, Head and Neck, Hepatocellular, Lymphoma, Melanoma, Neurological, Sarcoma, Upper Gastrointestinal, Urological, Lung Cancer  Australia | Assessment of multidisciplinary team performance and engagement of teams in continuous improvement  180 health professionals of 19 MDTs; twelve MDTs continued to full study  2017–2018 | Quantitative and qualitative data analysis  43-item survey before implementation and annual self-assessment matrix thereafter to score MDT performance across 20 domains | Wide variation in MDT maturity; positive responses increased between 2017 and 2018  Strengths: participation; collaboration; discussion quality  Barriers: lack of administrative support; IT issues; time constraints | Improvement tools to assess team performance for team-level improvement and broader management oversight |
| Hitz et al. (2022) [69]  Mixed-methods study  Cancer types: Gastrointestinal, Thoracic, Central Nervous System, Urological, Haematological, Non-Disease Specific Board, Breast Pre-operative, Breast Post-operative, Gynaecological, Head and Neck, Soft Tissue and Bone, Dermatological  Switzerland | Exploration of differences in multidisciplinary team meetings by tumour type and identification of organisational, leadership, and team factors influencing team performance  13 health professionals (qualitative) and 181 MDT members (quantitative); 40 medical oncologists, 79 surgeons, ten radio-oncologists, nine gastroenterologists, four haematologists, three neurologists, two dermatologists, nine radiologists, and ten pathologists  Study period not available | MDT-MODe and ATLAS (153 cases)  Interviews and observations  Assessment: organisational structure; leadership; teamwork: decision-making; perceived value/motivation | Challenges in MDT functioning: relevant information and presence of relevant cancer specialists consistent across tumour types | Factors critical for effective decision-making and team performance, regardless of the tumour focus  No systematic differences in teamworking across different tumour types; minimal tumour-specific variations emphasising the importance of general structural elements, like leadership and team collaboration |
| Lamb et al. (2013) [8]  Observational study  Cancer types: Breast, Colorectal, Lung, Gynaecological, Head and Neck, Upper Gastrointestinal, Urological, Haematological  United Kingdom | Investigation of MDT participants’ views on contributions to the session; consideration of patients’ perspective; management of differences of opinion during the session  Analysis of 1,636 MDT participants; physicians, nurses and healthcare staff, MDT coordination and other non-medical professions  January–March 2009 | Online survey  Analysis of six open questions on three main topics: (1) effective teamwork in meetings; (2) effectiveness of the recommendation (documentation of different opinions; main reasons for non-compliance with the recommendation); (3) patient-centredness in the decision-making process (patient advocacy during the meeting; communication with patients after the meeting) | Key issues: (1) importance of team skills, support from the organisation, good relationship between MDT participants; (2) documentation of disagreements, presentation of patient-centred information of importance; (3) central role of nursing as patient advocate | Development of team skills and support from the organisation for an effective MDT and patient-centred oncological care  Documentation of disagreements in decision-making as an important factor |
| Soukup et al. (2023) [70]  Cross-sectional observational study  Cancer types: Breast, Colorectal, Gynaecological  United Kingdom | Exploration of dynamics of group interaction and teamwork in MDT meetings  Three MDTs; 822 case discussions in 30 meetings; 44 health professionals  September 2015–July 2016 | Video recorded meetings for three months; combination of qualitative (conversation analysis) and quantitative (frequency counts) methods | Key findings: surgeons led with 47% speaking time; nurse specialists and coordinators spoke least (4% and 1%)  Disfluencies rose 45% in the second half, suggesting fatigue or complexity  Highly interactive discussions: each initiation prompted more than one response on average; breast/gynaecologic MDTs more declarative; colorectal more interrogative; higher-ranked professionals dominated discourse | Results emphasize the importance of teamwork in MDTs and to consider interaction patterns, cognitive load/fatigue in decision-making, hierarchies and patient perspectives in the planning of MDTs |
| Soukup et al. (2022) [64]  Secondary analysis of a cross-sectional observational study  Cancer types: Breast, Colorectal, Gynaecological  United Kingdom | Identification of different types of logistical issues and impact on team processes  Three MDTs; 822 case discussions in 30 meetings; 44 health professionals  September 2015–July 2016 | Observational instruments to measure decision-making, communication and case-complexity including logistical challenges: Metrics of Observational Decision-making, MDT-MODe, Bales’ IPA^[[4]](#footnote-4)^ | Administrative and process issues as most common issues (30%): significantly more often than attendance (16%) and equipment issues (5%) | Administrative and process issues most frequent logistical challenges  Reduced quality of decision-making due to unavailable diagnostic results and inadequate patient details |
| Soukup et al. (2020) [65]  Prospective cross-sectional observational study  Cancer types: Breast, Colorectal, Gynaecological  United Kingdom | Relationship between MDT's interaction processes/communication during a task and internal factors (team composition) and external factors (workload and time pressure)  30 weekly sessions in three MDTs over twelve weeks; 822 case presentations; 44 MDT participants; surgery; oncology; cancer nurses; radiology, pathology, MDT coordination  September 2015–July 2016 | Two validated instruments to assess individual case presentations:  MeDiC (measurement of case complexity according to clinical and logistical complexity)  Bales IPA (measurement of the frequency of task-oriented and socio-emotional communication)  Measurement of team size, gender distribution, presence of different disciplines, relationship between time and work pressure, time required per case | Association between patient case complexity and communication:  Higher work pressure linked to reduced communication and fewer positive interactions; increased time spent per case associated with reduced communication and positive interactions; negative interactions associated with time spent per case; team size and disciplinary diversity positively related to task-oriented communication and negative socio-emotional interactions | Enhanced MDT performance associated with smaller, gender-balanced teams, core discipline attendance, and reduced workload, time-on-task effects, and logistical issues |
| Tullis et al. (2022) [66]  Descriptive qualitative observational study  Cancer types: Breast, Skin, Blood and Bone Marrow  United States | Description of communication patterns in MDTs and extent to which non-biomedical issues are addressed  Three MDTs; 460 patient cases over six weeks  Study period not available | Real-time observation; field notes; focus on meeting structure, physical layout, communication patterns and content | Similarities in communication patterns in breast and skin MDTs: focus on medical facts; social issues discussed only in new cases  Different communication patterns in blood & bone marrow MDTs: quality of information and more narrative-based discussions; including social, emotional, and caregiver concerns | MDTs vary in communication style across entities; non-biomedical issues are often underrepresented |
| Walraven et al. (2023) [67]  Qualitative interview study  Cancer type not provided  The Netherlands | Identification of facilitators and barriers to run an efficient, competent and high-quality MDT  35 interviews with 16 medical specialists and 19 residents  May 2018–May 2019 | Individual semi-structured telephone interviews analysed with content analysis | Four key themes were identified (55 facilitators and 45 barriers): organisational aspects (e.g. structure, time, preparation); responsibilities and requirements (e.g. chairperson, leadership, team composition); competences and team dynamics (e.g. communication, hierarchy, team climate); meeting content (e.g., relevance of discussions, educational function) | Organisation, preparation, communication, and team dynamics are essential for a high-quality MDT  Recommendations include better IT use, formal training, and possible case streamlining to reduce time pressure |

**Table S4. Analysis and assessment of the decision-making process**

| **Author; year; study design; country; setting** | **Objectives; Sample** | **Methods** | **Results** | **Conclusion** |
| --- | --- | --- | --- | --- |
| Askelin et al. (2023) [84]  Qualitative descriptive study  Urological cancer  Australia | Exploration of clinical decision-making and the role of patient engagement  Five MDT members; oncologists, radiologists, nurses  Study period not available | Semi-structured interviews  Cognitive continuum theoretical model of clinical decision-making for analysis | Key findings: barriers and facilitators (attendance issues, late/incomplete referrals); virtual format improved attendance but reduced discussion quality; lack of person-centred decision-making (limited consideration of quality of life or psychosocial factors) | Uro-oncology MDTs focus on biomedical information, with limited attention to holistic, person-centred care; clinical decision-making often without structured clinical auditing |
| Dew et al. (2015) [85]  Qualitative observational study  Cancer types: Breast, Lung, Upper Gastrointestinal, Colorectal  New Zealand | Investigation of the decision-making process in MDTs  Ten MDTs with 106 case discussions  Study period not available | Description of processes and activities in the MDT; conversation analysis and membership categorisation analysis | Categorisation of cases is based on cancer type: different dimensions of authority, values, strategies for justifying reactions; chairperson’s important role in the decision-making process | Improvement of outcomes and decision-making through consideration of MDT processes, values, and authority structures |
| Hahlweg et al. (2017) [125]  Cross-sectional observational study  Cancer types: Dermatological, Gastrointestinal, Gynecological, Head and Neck, Liver and Biliary Tract, Lymphoma and Myeloma, Neurological, Non-Entity-Specific Oncological, Non-Entity-Specific Surgical, Thorax and Urological  Germany | Documentation of the decision-making process and the type of recommendation (none, single, multiple); identification of factors that influence the decision  Eleven MDTs; 249 case presentations in 29 sessions  September–October 2014 | Adaptation of MDT-MODe to evaluate the quality of presented information and team processes | Recommendation was made in 64% of cases, none in 25% and several alternatives in 10%  No recommendations associated with discussion duration, quality of medical history, radiological information, or MDT specialisation | Perceived variation in information quality; lack of patient-centredness in MDT recommendations; association between information quality and recommendations; time pressure as a significant factor in the decision-making process |
| Jalil et al. (2013) [62]  Qualitative study  Cancer types: Urological, Gastrointestinal  United Kingdom | Analysis of the decision-making process and compliance with recommendation  Twenty-two MDT-participants from three hospitals  February 2012 | Semi-structured expert interviews  Evaluation of the decision-making process from the expert perspective: barriers and practical implementation | Video conferencing negatively impacts decision-making (technical issues, teamwork)  Decision-making barriers: incomplete patient information; lack of key disciplines; time pressure  Recommendations adherence influenced by consideration of patient-relevant factors during the MDT | Improvement strategies: better case preparation, effective team leadership, involvement of anaesthetic to set or rule out surgery dates  Controversial opinions on patient participation in the MDT |
| Koo et al. (2025) [88]  Quantitative prospective observational study  Cancer types: Breast, Lung, Colorectal, Gastrointestinal, Upper Gastrointestinal, Genitourinary, Gynaecology, Skin, Head and Neck, Central Nervous System, Myeloma, Lymphoma  Australia | Evaluation of MDT decision-making quality using a validated assessment tool and potential for improvement  498 patients in 14 MDTs; three cancer centres  July 2022–June 2023 | MDT-MODe  Variation of decision-making quality between cancer types; identification of domains that require improvement | Medical information (radiology, pathology) presented excellently; psychosocial factors, comorbidities, and patient preferences poorly addressed compared to medical factors; cancer specialist nurses scored lowest despite attending 77% of meetings; treatment recommendations reached in 99.4% of cases | High standards of medical information presentation but variable team contribution  Targeted interventions to improve decision-making quality, balance specialty contributions, and enhance patient-centred care in MDTs |
| Lamb et al. (2013) [73]  Longitudinal study  Urological cancer  United Kingdom | Evaluation of the quality of the decision-making process and teamwork after introduction of different measures  1,421 case presentations in 36 MDTs; surgery, oncology, specialists, study nurses; radiology; pathology; non-medical MDT coordinator  December 2009–April 2011 | Assessment of teamwork with MDT-QuIC and MDT-MODe  Multicomponent intervention design; before-after measurement over 16 months with different points in time after different interventions | Team training, introduction of the checklist, and case structuring led to a continuous improvement in the decision-making process and teamwork | Teamwork quality positively linked to recommendations at initial patient presentation; cumulative interventions improved the quality of teamwork and the possibility of a recommendation at first patient presentation |
| Lamb et al. (2011) [72]  Qualitative study  Cancer types not provided  United Kingdom | Investigation of attitudes and experiences of MDT participants; identification of factors that promote and hinder effective teamwork  19 MDT participants; surgery, oncology, nursing, administration  October 2009–April 2010 | Quantitative and qualitative analysis of semi-structured interviews  Survey on the topics: attendance, presentation of information, case discussion, management/leadership, decision-making process, potential for improvement | Non-participation associated with insufficient time in daily work  Improvement potentials: scheduled MDT participation; structured workflows, case selection | Results consistent with previous studies: participants have a positive attitude towards the MDT, but there is still room for improvement |
| Luijten et al. (2022) [86]  Qualitative study  Esophagogastric cancer  The Netherlands | Exploration of facilitators and barriers for clinical decision-making during MDTs  16 MDTs; 30 interviews with health professionals in eight hospitals; seven focus groups with MDT members  Study period not available | Observations and semi-structured interviews  Thematic content analysis | Team dynamics and individual clinician characteristics (ambition and innovation) significantly influence the decision-making process  Key components: communication quality; leadership; psychological safety; team composition; mutual critique and dominance of personalities | Communication styles and relationships might impact the effectiveness of clinical decision-making; essential for addressing the variations in treatment practices across hospitals and enhancing team performance |
| Lumenta et al. (2019) [74]  Feasibility study  Observational study  Cancer types not provided  Austria | Testing of the adapted MDT-MODe  244 case presentations in 27 sessions  Study period not available | Adaptation of the MDT-MODe and German translation  Key aspects of team performance: communication style; team rules, culture | 56% fast-track cases; 44% complex cases; 93% recommendations made; broad agreement between observers regarding items | Quality of decision-making process depends on quality of presented information, team performance and infrastructure (organisation and logistics) |
| Marrara et al. (2023) [75]  Pilot study  Thoracic cancer  United States | Assessment of the applicability of MDT-MODe  44 case discussions over six weeks; 11 radiologists and pathologists, 63 clinicians  Study period not available | MDT-MODe  Audio recordings | Key areas for improvement: comorbidities and patient perspectives; increased pathologist and radiologist input; information quality; management of case volume | MDT-MODe developed for urology is adaptable across cancer centres and thoracic MDTs |
| Rosell et al (2018) [76]  Cross-sectional study  Cancer types: Gastrointestinal and Hepatobiliary, Breast, Malignant Melanoma, Urological, Gynaecological, Lung, Head and Neck, Central Nervous System, Sarcomas, Endocrine Tumours  Sweden | Investigation of the attitude of health professionals towards the function of an MDT including perceived advantages and disadvantages of a session.  244 health professionals from 50 MDTs  Study period not available | Online survey  Quantitative assessment of structure and function of MDTs according to usefulness of the meeting and difficulty in making a recommendation | Benefits: patient management support; inclusion in clinical trials; more accurate treatment recommendations; multidisciplinary assessment; adherence to guidelines  Difficulties: incomplete histologic results | Health professionals report some benefits of MDTs, but also areas for improvement (access to complete information, clear allocation of roles between different disciplines) |
| Scott et al. (2020) [77]  Cross-sectional observational study  Gynaecological cancer  United Kingdom | Investigation of decision-making in advanced ovarian cancer in gynaecological MDTs  Four MDTs in six hospitals; 41 case discussions; gynaecology, breast medicine, oncology, cancer nurses, radiology, pathology, etc.  March 2019 | GO-MDT-MODe^^[[5]](#footnote-5)^^  Case information and contributions from seven disciplines were rated on a five-point Likert scale | 41 recommendations were made for patients with advanced ovarian cancer | Decision-making influenced by four factors: medical history; tumour markers; imaging; radiology contribution; patient-relevant factors underrepresented |
| Shah et al. (2016) [79]  Observational study  Head and neck cancer  United States | Evaluation of the integration of guidelines into the decision-making process  One MDT; 176 case presentations; surgery, oncology, diagnostic neuroradiology; speech therapy and others  March 2010–October 2021 | Extent to which guidelines are integrated into the decision-making process and further treatment | Evidence-based recommendation was made for 98.3% of patients; 78% of the recommendations corresponded to the highest available evidence-based guideline; 87% of patients received evidence-based further treatment | Approximately 80% of patients receive a recommendation based on the highest level of evidence |
| Shah et al. (2014) [78]  Observational study  Colorectal cancer  United Kingdom | Performance evaluation of colorectal cancer MDTs  267 case presentations in 11 sessions; one weekly MDT; surgery (chair), radiology, oncology, pathology, cancer nurse; administration, trainees  March–August 2012 | cMDT-MODe  Evaluation of the decision-making process regarding the quality of information (medical history, comorbidities, psychosocial factors, patient perspective, radiologic and histologic findings) and participants' contributions to the discussion | Duration of 76 minutes; approximately 16 participants, average of 24 cases (3 minutes per case)  Above average: medical history and information from radiology and pathology (biomedical information)  Below average: patient perspective and psychosocial information | Below-average rating of patient information is due to incomplete information |
| Soukup et al. (2019) [83]  Observational study  Breast cancer  United Kingdom | Investigating the effectiveness (in team audits) of interventions (feedback rounds) to improve quality in MDTs with high workload and longer meetings  one MDT with 15 MDT participants; 1,335 case presentations  2013–2015 | MDT-MODe  Determining the occurrence and effects of fatigue in the decision-making process on team performance; effects of a short session break against fatigue in the decision-making process | Before intervention, decision-making process in meetings showed noticeable fatigue  After intervention, the quality of information and discussions improved | Long meetings and a high number of case presentations cause MDT participants to become tired during the decision-making process, which reduces the quality of the decision-making process; by introducing breaks in the middle of the session, fatigue can be counteracted |
| Soukup et al. (2020) [80]  Cross-sectional observational study  Cancer types: Breast, Colorectal, Gynaecological  United Kingdom | Analysis of processes and interactions of a group decision in MDTs  Three MDTs over three months; 44 MDT participants; 822 case presentations over 30 sessions; surgery, radiology, oncology, pathology, cancer nurse; administrator  September 2015–July 2016 | Three validated instruments: MODe; MeDiC; Bales IPA | Socio-cognitive predictors influence the decision-making process | Barriers: socio-emotional reactions; with each decision, participants' contributions decreased (largest decrease in the second half)  Facilitators: question wording, answers, team size, gender balance |
| Soukup et al. (2016) [81]  Quantitative observational study  Cancer types: Breast, Lung, Bowel, Urological  United Kingdom | Evaluation of the decision-making process and its impact on the team's ability to make decisions  Quality assessment of 1,045 case reviews in four MDTs; chair and coordination, surgery, oncology, radiology, pathology; oncology nursing, thoracic specialist in lung board  2010–2014 | MDT-MODe  Quality of information presented and contribution to the discussion of core disciplines  Assessment of treatment decision outcome after (yes/no) | Ability of the team to come to a recommendation depends on the information presented: psychosocial information increased the ability to decide, while information on comorbidities made it more difficult | Influence of all disciplines on the decision-making process is significant and supports the multidisciplinary approach; driven by clinical information  Contributions from nursing and comorbidities reduce the likelihood of a recommendation (increase in case complexity); psychosocial information is underrepresented |
| Soukup et al. (2016) [82]  Cross-sectional observational study  Cancer types: Breast, Lung, Bowel, Urological  United Kingdom | Investigation of underlying factors of the decision-making process  Four MDTs; 52 MDT participants; 1,045 case discussions in ten consecutive sessions  2010–2014 | MDT-MODe | Key factors: transparent clinical contributions; pathology; radiology, session management; complete patient information | Factors identified in the decision-making process relate to all core disciplines as well as complete patient information |
| Taylor et al. (2021) [18]  Mixed-methods evaluation  Breast cancer  United Kingdom | Assessment of MDT-FIT implementation in breast MDTs  Ten MDTs; 36 interviews; 275 members, including medical, nursing, and administrative staff  Study period not available | Data collection: process and systems logs; interviews  Implementation involved three stages: (1) setup; (2) assessment (which included self-reports and independent observational assessments); (3) team feedback discussions to agree on actions for improvement | Acceptability, appropriateness, and feasibility of the MDT-FIT implementation | It is feasible to implement a co-designed team improvement program across a large integrated care system |
| Wihl et al. (2021) [63]  Observational study  Cancer types: Neurological; Soft Tissue Sarcoma, Liver  Sweden | Mapping of medical and non-medical patient information during case presentations and discussions in MDTs  Three MDTs; 30 MDT sessions; 336 case presentations; surgery, oncology, neurology, radiology, pathology, cancer nurses  April–October 2019 | Collection of information about medical and non-medical information and patient-related characteristics (qualitative and quantitative)  Classification according to subject areas | Information presented in case presentations: 48.2% on physical status; 8.9% on mental status; 48.5% on comorbidities  Non-medical information: family information; occupation; country of origin presented in 3.6–7.7% of cases; patient preferences were presented in 4.2% of cases | Information on comorbidities presented in 50% of cases; non-medical information presented in less than 10% of cases |

**Table S5.** **Impact of MDT performance on patient management**

| **Author; year; study design; country; setting** | **Objectives; sample** | **Methods** | **Results** | **Conclusion** |
| --- | --- | --- | --- | --- |
| Brauer et al. (2017) [23]  Observational study  Cancer types: Pancreas, Liver  United States | Investigation of the decision-making process and resource expenditure of MDTs  Data collection over 12 months; one MDT; 470 case presentations; surgery, oncology, interventional gastroenterology, radiology, pathology, other specialist disciplines  September 2014–August 2015 | Prospective data collection of the decision-making process, including a record of the pre-conference plan  Research questions: change in treatment plan because of discussions; resources for holding MDTs; survival; guideline-compliant recommendations | In 25.1% of cases, treatment plan changed (further diagnostic investigations) | Influence of MDTs on treatment plans with 100% adherence to guidelines; MDTs take a lot of time, possibly without outcome |
| El Saghir et al. (2015) [103]  Quantitative cross-sectional study  Cancer types: Breast, Gastrointestinal  International | Evaluation of global practice, efficiency, and impact of MDTs on cancer management  501 responses; international survey with ASCO members practicing outside the United States  October 2013 | Quantitative survey: attendance; frequency; disciplines involved; changes in treatment plans; perceived benefits; suggestions for improvement | Changes in treatment plans for approximately 44–50% of breast and colorectal cancer patients, with treatment or surgical adjustments in 12–50%  Changes in management more often when cases presented by less experienced physicians (<15 years) | MDTs contribute positively to patient management and medical education; improved efficiency through better organisation, case selection or leadership |
| Francisse et al. (2023) [26]  Retrospective observational study  Pancreatic cancer  Belgium | Impact of MDT discussion on management of diagnosis of focal pancreatic lesions  Review of patient files discussed at MDT meetings  January 2020–December 2021 | Data collection on management and diagnosis modifications, criteria leading to changes, and timing | Management modifications in 72.2% of cases, predominantly driven by radiological review; diagnosis altered in 17.6%, more often in cystic lesions (35.7%) than solid lesions (7.7%) | MDT discussions significantly impact management and diagnostic accuracy, mainly through imaging review |
| Gandamihardja et al. (2019) [104]  Prospective observational study  Breast cancer  United Kingdom | Evaluation of team working in MDTs  Ten MDT meetings; 346 case discussions  Study period not available | MDT-MODe  Quality of presented information and contribution from team members in real-time | Management decisions made in 99% of cases  Surgeons contributed most, patient information largely of a biomedical content  Approximately 42 patients were discussed per meeting, over three hours with an average of 3min and 20s per patient | Differences in contribution levels among team members; lower engagement of nurses; lower psychosocial and patient-centred information |
| Mori et al. (2018) [29]  Cross-sectional study  Skin cancer  United States | Analysis of the structure, function and effect of the MDT  38 MDT managers; dermatology, surgery, oncology  2017 | Online survey | Moderate to significant impact of MDTs om patient management; improvement of communication between disciplines | General consistency in structure of MDTs, though fluctuations in function |
| Petrella et al. (2021) [105]  Retrospective observational study  Lung cancer  Italy | Influence of the MDT on clinical treatment pathway; extent to which outpatient diagnosis changes after presentation in the MDT  1,000 patient cases; one MDT; oncology; radiology; interventional pulmonology; thoracic surgery; radiation oncology; reference nursing; assistant physicians; pathology  2019 | Influence of the MDT divided into confirmation of the outpatient hypothesis and change in diagnosis  Implementation: definition of a clear treatment pathway/recommendation for patients who have not yet received a diagnosis; further diagnostics required | Impact on lung cancer patient management in 10.6% of cases | Impact of MDTs on patient management is higher than their impact on survival rates |

**Table S6. Implementation of virtual boards (facilitators and barriers)**

| **Author; year; study design; country; setting** | **Objectives; sample** | **Methods** | **Results** | **Conclusion** |
| --- | --- | --- | --- | --- |
| Ali et al. (2023) [24]  Cross-sectional study  Skin cancer  United Kingdom | Effectiveness of virtual skin MDTs (data security, confidentiality, decision-making, efficiency, and organisation)  Experience of virtual meetings (teamwork, training, and engagement)  68 respondents of 36 MDTs  Study period not available | Quantitative questionnaire with free text responses  Topics: communication, chairing and decision-making; team working and engagement; training; clinical trials recruitment; audit; technology and security | MDT work stayed the same during the transition to virtual meetings, but teamwork and training declined; adequate technology, infrastructure and security | Managing MDTs can be challenging due to treatment inconsistency, costs, and opportunity costs; virtual meetings may allow remote participation; challenges include technology reliability and reduced interaction |
| Dharmarajan et al. (2020) [30]  Feasibility study  Head and neck cancer  United States | Implementation of a virtual MDT and assessment of implementation process (plan-do-study-act)  3 Three virtual MDT sessions; 19 MDT members  Study period not available | Advantages and disadvantages of virtual MDTs | Documentation system structured and goal-oriented; MDT case database as central resource for reviewing patient pathways, treatment plans, outcomes, and adherence to guidelines; implications for analysing patient data | Design and implementation of virtual MDTs is feasible in a academic medical network |
| Groothuizen et al. (2023) [110]  Mixed methods study  Lung cancer  United Kingdom | Members' perception of virtual lung cancer meetings (IT issues and distractions)  Eight MDTs; 73 MDT members participated quantitative and 41 qualitative; respiratory physicians, surgeons, oncologists, radiologists, pathologists, palliative care professionals, nurses and coordinators  April–August 2021 | Semi-structured online interviews; online survey  Real­time observations of virtual MDTs | Significant differences between teams in terms of IT functionality  Benefits: increased flexibility; reduced travel time; easier real-time access to patient information | Ensuring functioning IT could facilitate optimal decision-making |
| Hirth et al. (2023) [106]  Monocentric retrospective study  Musculoskeletal cancer  Germany | Comparison of in-person and virtual MDTs  25 in-person and 27 virtual meetings  September 2019–February 2020 and May 2020–October 2020 | Analysis of first-time case discussions by review of charts | Positive effects on timely diagnosis and multidisciplinarity; improvement of decision-making | Virtual MDTs were a valid alternative to in-person meetings during the pandemic  Attendance of experts from remote areas could be facilitated |
| Mohamedbai et al. (2021) [107]  Quantitative observational study  Head and neck cancer  United Kingdom | Effectiveness of virtual MDT meetings; areas for improvement  97 responses; 27 MDTs; coordinators/administrative staff dieticians, nurses, oncologists, pathologists, radiologists, speech and language therapists and surgeons  December 2020 and February 2021 | Online questionnaire | Most clinicians (70.1%) reported decision-making unchanged with virtual MDT meetings; 84.5% found technology resources satisfactory; majority perceived engagement (43.9%), team working (69.1%), and training (47.7%) worse since moving to remote meetings | Virtual MDT meetings can enable efficient practice and decision making; Improvements are needed in engagement, training, and team working |
| Perlmutter et al. (2022) [28]  Quantitative study  Cancer types: Colorectal, Breast, Liver  United States | Description of benefits and challenges of virtual MDTs during COVID-19  253 respondents; 25 MDT meetings  September–October 2020 | Quality improvement survey: strengths and weaknesses of in‐person and virtual meetings | Key factors: consistent leadership and organisation; active participation from all attendees; improvements to IT infrastructure | Numerous challenges with virtual MDTs (e.g.) quality of conversation and social aspects to network with colleagues  Hybrid meetings are considered for improvement strategies |
| Salami et al. (2015) [108]  Retrospective cohort study  Hepatobiliary cancer  United States | Evaluation of the impact of a regional virtual MDT on patient care  116 patients included: 48 discussed in a virtual MDT (41%), and 68 discussed in an in-personal MDT; consultation  2009–2013 | Comparison of 116 patients referred via virtual MDTs vs. non-virtual MDTs  Evaluation of MDT quality, timeliness, and travel burden | Higher comprehensive recommendations in virtual MDT group (91.7% vs. 64.7%)  Faster evaluation (23 vs. 39 days) | Virtual MDTs are more convenient, facilitating timely recommendations and circumventing travel burdens |
| Schäfer et al. (2021) [109]  Quantitative cross-sectional study  Neurological cancer  Germany | Evaluation of virtual MDT implementation during COVID-19  65 responses from neurological centres; one representative per centre invited to the survey  March 2021 | 24 items online survey: structure, use and changes during the pandemic | 95% of centres had neurological MDTs (mostly established >3 years); 68% increased virtualisation due to COVID-19; 23% fully virtual | Virtual MDTs were feasible, accepted, and enhanced access, flexibility, and networking: potential for broader neuro-oncology care coverage |
| van Huizen et al. (2021) [111]  Mixed-methods study  Head and neck cancer  The Netherlands | Evaluation of virtual MDT number of recommendations made and the qualitative benefits)  336 patient cases; interviews with six health professionals  September 2016–March 2017 | Quantitative study to assess recommendation registration  Semi-structured interviews to explore the added value of virtual meetings | Recommendations were made in only 2% of the cases (alternate treatment plans or changes in diagnosis)  Discussions for complex cases provide new perspectives | Discussions helped maintain alignment in medical viewpoints; benefit of videoconference is minimal  Requirement for routine patient discussions may not enhance treatment quality |

**Table S7. Resource requirements of MDTs**

| **Author; year; study design; country; setting** | **Objectives; sample** | **Methods** | **Results** | **Conclusion** |
| --- | --- | --- | --- | --- |
| Alexandersson et al. (2018) [112]  Observational study  Cancer types: Breast and Malignant Melanoma, Upper Gastrointestinal (Including Oesophageal, Gastric, Hepatobiliary and Pancreatic), Lung, Colorectal, Urologic (Prostate, Renal Cell and Urothelial), Head and Neck, Gynaecologic, Sarcoma, Endocrine Tumours, Central Nervous System Tumours, Penile  Sweden | Evaluation of MDT costs  50 MDTs; 206 health professionals  February–July 2016 | Participation and survey  Collection of structural data (e.g. attendance, number of cases presented) | Doctors spent an average of 4.1 hours per meeting preparing, attending and follow-up; average cost per case discussion was €212, average cost per board was €2,675 | Major differences in resources required; 84% of total costs can be attributed to time spent by health professionals |
| Ali et al. (2021) [113]  Cross-sectional study  Skin cancer  United Kingdom | Evaluation of the composition, quoracy and cost of MDTs to assess the functionality and financial impact  58 health professionals (89% response rate)  July 2019 | Data collection: attendance frequency and relevant disciplines from attendance registers  Costs based on running time, core disciplines salaries derived from national pay scales, and overhead values | 15 MDTs (26%) were quorate by membership, while 40 MDTs (69%) were quorate by meeting frequency  The mean total cost was £3;963.68, varying from £946.12 to £9;353.94; mean cost per patient discussed was £132.68, with costs ranging from £31.67 to £313.10  Quoracy given if at least one dermatologist, one surgeon, one clinical oncologist, one medical oncologist, one histopathologist, one imaging specialist, one skin nurse specialist, and one MDT coordinator attended | Significant variability in costs across different teams |
| De Leso et al. (2013) [114]  Observational study  Cancer types: Breast, Urological, Gynecological, Renal, Melanoma, Lung, Head and Neck/Thyroid, Sarcoma, Lymphoma, Colorectal, Upper GI and Hepatobiliary Tract  United Kingdom | Evaluation of decision outcome and financial costs  Cost analysis; 551 case presentations; 14 MDTs; 52 sessions; supplement: 81 case presentations from ten other boards  June 2010 | Data extraction from documentation  Cost analysis by attendance at 14 MDTs and by preparation time including active participation in the board (in four larger MDTs) | Costs per month for one MDT between £2,192 and £10,050; total costs for all 14 MDTs: £80,850; costs for each initial appointment £415  Costs for four MDTs between £14,430 and £38,327 per month | MDTs are costly; nevertheless, there is a need to improve MDT efficiency without losing associated considerable benefits |
| Mullan et al. (2014) [27]  Observational study  Head and neck cancer  United Kingdom | Evaluation of initial presentations (time required per case, number of participants and type of case)  105 case presentations in ten sessions  January–February and August–September 2011 | Measurement of time required per case, number of participants and time taken to discuss patient-relevant data | Longer discussions in later-staged patients; earlier-stage patients discussed briefly to have time for more complex cases | Many patients discussed only briefly; suggestions for fast-track approaches |
| Sassé et al. (2025) [115]  Prospective observational study  Metastatic breast cancer  Australia | Impact of MDTs on management plans for patients (implementation rate of these plans, and associated costs and benefits)  Consecutive patients in MDTs  October 2023–July 2024 | Recorded management plans before and after meeting  Plans categorised into no change, low impact, or high impact (high impact defined as major treatment changes or trial referrals)  Follow-up data collected four months post-MDT to assess planned implementation | 65 (57.0%) MDT presentations resulted in changes to the management plan, with 32 (28.1%) classified as high impact  Presentations with follow-up data, 65 (84.4%) were fully implemented, and 39 out of 42 high impact recommendations were implemented (92.6%)  The MDT earned $571.18 per week with estimated costs of $1,584.63 | Metastatic breast cancer MDTs lead to significant changes in patient management, with a high implementation rate of changes |

1. GO-MDT-MODe: Gynaecological Oncology Multi-Disciplinary Team Metric of Decision-making [↑](#footnote-ref-1)
2. cMDT-MODe: Colorectal Multidisciplinary Team Metric for Observation of Decision-Making [↑](#footnote-ref-2)
3. MDT-OARS: Observational Assessment Rating Scale [↑](#footnote-ref-3)
4. Bales’ IPA: Bales’ Interaction Process Analysis [↑](#footnote-ref-4)
5. GO-MDT-MODe: Gynaecological Oncology Multi-Disciplinary Team Metric of Decision making [↑](#footnote-ref-5)
