# Supplementary material for: Tumour boards and their quality of structures, processes, and team performance in multidisciplinary cancer care: a systematic review
Source: BMC Health Serv Res. 2026 Mar 31;26:676. doi: 10.1186/s12913-026-14447-9 (PMC13162496; doi:10.1186/s12913-026-14447-9)
Supplement: Supplementary file 4 — Supplementary Material 4: Supplement D - Included and excluded articles with reason [file 12913_2026_14447_MOESM4_ESM.docx]

**Included with reason - citation search**

| **References** | **Inclusion criteria** |
| --- | --- |
| Dew K, Stubbe M, Signal L, Stairmand J, Dennett E, Koea J, et al. Cancer care decision making in multidisciplinary meetings. Qualitative Health Research. 2015;25:397–407. https://doi.org/10.1177/1049732314553010. | **Decision-making** |
| Edney LC, Gray J, Karnon J. A scoping review of the economics of multidisciplinary teams in oncology care. Journal of Cancer Policy. 2020;26:100257. https://doi.org/10.1016/j.jcpo.2020.100257. | **Systematic and scoping review** |
| Evans L, Donovan B, Liu Y, Shaw T, Harnett P. A tool to improve the performance of multidisciplinary teams in cancer care. BMJ Open Quality. 2019;8:e000435. https://doi.org/10.1136/bmjoq-2018-000435. | **Team performance** |
| Lamb BW, Green JSA, Benn J, Brown KF, Vincent CA, Sevdalis N. Improving decision making in multidisciplinary tumor boards: Prospective longitudinal evaluation of a multicomponent intervention for 1,421 patients. Journal of the American College of Surgeons. 2013;217:412–20. https://doi.org/10.1016/j.jamcollsurg.2013.04.035. | **Decision-making** |
| Lamb BW, Sevdalis N, Arora S, Pinto A, Vincent C, Green JSA. Teamwork and team decision-making at multidisciplinary cancer conferences: Barriers, facilitators, and opportunities for improvement. World Journal of Surgery. 2011;35:1970–6. https://doi.org/10.1007/s00268-011-1152-1. | **Decision-making** |
| Lamb BW, Taylor C, Lamb JN, Strickland SL, Vincent C, Green JSA, et al. Facilitators and barriers to teamworking and patient centeredness in multidisciplinary cancer teams: Findings of a national study. Annals of Surgical Oncology. 2013;20:1408–16. https://doi.org/10.1245/s10434-012-2676-9. | **Team performance** |
| Mullan BJ, Brown JS, Lowe D, Rogers SN, Shaw RJ. Analysis of time taken to discuss new patients with head and neck cancer in multidisciplinary team meetings. The British Journal of Oral & Maxillofacial Surgery. 2014;52:128–33. https://doi.org/10.1016/j.bjoms.2013.10.001. | **Resource requirements** |
| Ottevanger N, Hilbink M, Weenk M, Janssen R, Vrijmoeth T, Vries A, et al. Oncologic multidisciplinary team meetings: Evaluation of quality criteria. Journal of Evaluation in Clinical Practice. 2013;19:1035–43. https://doi.org/10.1111/jep.12022. | **Quality and processes** |
| Rajan S, Foreman J, Wallis MG, Caldas C, Britton P. Multidisciplinary decisions in breast cancer: Does the patient receive what the team has recommended? British Journal of Cancer. 2013;108:2442–7. https://doi.org/10.1038/bjc.2013.267. | **Adherence to recommendations** |
| Rankin NM, Lai M, Miller D, Beale P, Spigelman A, Prest G, et al. Cancer multidisciplinary team meetings in practice: Results from a multi-institutional quantitative survey and implications for policy change. Asia-Pacific Journal of Clinical Oncology. 2018;14:74–83. https://doi.org/10.1111/ajco.12765. | **Quality and processes** |
| Robinson TE, Janssen A, Harnett P, Museth KE, Provan PJ, Hills DJ, et al. Embedding continuous quality improvement processes in multidisciplinary teams in cancer care: Exploring the boundaries between quality and implementation science. Australian Health Review. 2017;41:291–6. https://doi.org/10.1071/AH16052. | **Quality and processes** |
| Shah BA, Qureshi MM, Jalisi S, Grillone G, Salama A, Cooley T, et al. Analysis of decision making at a multidisciplinary head and neck tumor board incorporating evidence-based National Cancer Comprehensive Network (NCCN) guidelines. Practical Radiation Oncology. 2016;6:248–54. https://doi.org/10.1016/j.prro.2015.11.006. | **Decision-making** |
| Shah S, Arora S, Atkin G, Glynne-Jones R, Mathur P, Darzi A, et al. Decision-making in colorectal cancer tumor board meetings: Results of a prospective observational assessment. Surgical Endoscopy. 2014;28:2783–8. https://doi.org/10.1007/s00464-014-3545-3. | **Decision-making** |
| Soukup T, Lamb BW, Shah NJ, Morbi A, Bali A, Asher V, et al. Relationships between communication, time pressure, workload, task complexity, logistical issues and group composition in transdisciplinary teams: A prospective observational study across 822 cancer cases. Frontiers in Communication. 2020;5:583294. https://doi.org/10.3389/fcomm.2020.583294. | **Team performance** |

**Excluded with reason – citation search**

| **References** | **Exclusion criteria** |
| --- | --- |
| Cancer Research UK. Meeting patients’ needs: Improving the effectiveness of multidisciplinary team meetings in cancer services. 2020. | **No study** |
| Devitt B, Philip J, Madhu S, McLachlan S-A. Understanding patients’ attitudes toward cancer multidisciplinary meetings: A mixed methods study. Journal of Oncology Practice. 2020;16:1–7. | **Patient involvement** |
| Diekmann A, Heuser C, Schellenberger B, Bohmeier B, Holmberg C, Ansmann L, et al. Patient participation in multidisciplinary tumor conferences: Providers’ perceptions of patients’ need satisfaction and emotional experiences. Psycho-Oncology. 2020;29:1263–71. https://doi.org/10.1002/pon.5413. | **Patient involvement** |
| Engelhardt M, Selder R, Pandurevic M, Möller M, Ihorst G, Waldschmidt J, et al. Multidisziplinäre Tumorboards: Fakten und Zufriedenheitsanalyse eines unverzichtbaren Instruments von Tumorzentren. Deutsche medizinische Wochenschrift. 2017;142:e51–60. https://doi.org/10.1055/s-0043-100054. | **Patient involvement** |
| Epstein RM, Street RL. The values and value of patient-centered care. Annals of Family Medicine. 2011;9:100–3. https://doi.org/10.1370/afm.1239. | **Topic** |
| Fahim C, McConnell MM, Wright FC, Sonnadara RR, Simunovic M. Use of the KT-MCC strategy to improve the quality of decision making for multidisciplinary cancer conferences: A pilot study. BMC Health Services Research. 2020;20:579. https://doi.org/10.1186/s12913-020-05143-3. | **Technical support system/AI** |
| Heuser C, Diekmann A, Schellenberger B, Bohmeier B, Kuhn W, Karbach U, et al. Patient participation in multidisciplinary tumor conferences from the providers’ perspective: Is it feasible in routine cancer care? Journal of Multidisciplinary Healthcare. 2020;13:1729–39. https://doi.org/10.2147/JMDH.S283166. | **Patient involvement** |
| Homayounfar K, Lordick F, Ghadimi M. Qualitätssicherung: Multidisziplinäre Tumorboards – trotz Problemen unverzichtbar. Deutsches Ärzteblatt. 2014;111:A 998-A 1001. | **Language** |
| Homayounfar K, Mey D, Boos M, Gaedcke J, Ghadimi M. Kommunikation im Tumorboard. Forum. 2015;30:214–7. https://doi.org/10.1007/s12312-015-1301-9. | **Language** |
| Johnson CE, Slavova-Azmanova N, Saunders C. Development of a peer-review framework for cancer multidisciplinary meetings. Internal Medicine Journal. 2017;47:529–35. https://doi.org/10.1111/imj.13374. | **Topic** |
| Lamb BW, Payne H, Vincent C, Sevdalis N, Green JSA. The role of oncologists in multidisciplinary cancer teams in the UK: An untapped resource for team leadership? Journal of Evaluation in Clinical Practice. 2011;17:1200–6. https://doi.org/10.1111/j.1365-2753.2010.01507.x. | **Profession** |
| Lewis CM, Nurgalieva Z, Sturgis EM, Lai SY, Weber RS. Improving patient outcomes through multidisciplinary treatment planning conference. Head & Neck. 2016;38:E1820–5. https://doi.org/10.1002/hed.24325. | **Topic** |
| Linford G, Egan R, Coderre-Ball A, Dalgarno N, Stone CJL, Robinson A, et al. Patient and physician perceptions of lung cancer care in a multidisciplinary clinic model. Current Oncology. 2020;27:e9–19. https://doi.org/10.3747/co.27.5499. | **Sample** |
| Meguid C, Ryan CE, Edil BH, Schulick RD, Gajdos C, Boniface MM, et al. Establishing a framework for building multidisciplinary programs. Journal of Multidisciplinary Healthcare. 2015;8:519–26. https://doi.org/10.2147/JMDH.S96415. | **Topic** |
| National Cancer Action Team. The characteristics of an effective multidisciplinary team (MDT). 2010. | **No study** |
| Osarogiagbon RU. Overcoming the implementation gap in multidisciplinary oncology care programs. Journal of Oncology Practice. 2016;12:888–91. | **Topic** |
| Strebel RT, Sulser T, Schmid H-P, Gillessen S, Fehr M, Huber U, et al. Multidisciplinary care in patients with prostate cancer: Room for improvement. Supportive Care in Cancer. 2013;21:2327–33. https://doi.org/10.1007/s00520-013-1791-x. | **Medical outcome** |
| Ung KA, Campbell BA, Duplan D, Ball D, David S. Impact of the lung oncology multidisciplinary team meetings on the management of patients with cancer. Asia-Pacific Journal of Clinical Oncology. 2016;12:e298–304. https://doi.org/10.1111/ajco.12192. | **Patient management without teamwork** |

**Included with reason – systematic search**

| **References** | **Inclusion criteria** |
| --- | --- |
| Alexandersson N, Rosell L, Wihl J, Ohlsson B, Steen Carlsson K, Nilbert M. Determinants of variable resource use for multidisciplinary team meetings in cancer care. Acta Oncologica. 2018;57:675–80. https://doi.org/10.1080/0284186X.2017.1400682. | **Resource requirements** |
| AlFarhan HA, Algwaiz GF, Alzahrani HA, Alsuhaibani RS, Alolayan A, Abdelhafiz N, et al. Impact of GI tumor board on patient management and adherence to guidelines. Journal of Global Oncology. 2018;4:1–8. https://doi.org/10.1200/JGO.17.00164. | **Adherence to recommendations** |
| Ali SR, Dobbs TD, Hutchings HA, Whitaker IS. Composition, quoracy and cost of specialist skin cancer multidisciplinary team meetings in the United Kingdom. Journal of Plastic, Reconstructive & Aesthetic Surgery. 2021;74:3335–40. https://doi.org/10.1016/j.bjps.2021.05.019. | **Resource requirements** |
| Ali SR, Dobbs TD, Jovic M, Hutchings HA, Whitaker IS. Improving the effectiveness of multidisciplinary team meetings on skin cancer: Analysis of the National Cancer Research UK survey responses. Journal of Plastic, Reconstructive & Aesthetic Surgery. 2023;82:141–51. https://doi.org/10.1016/j.bjps.2023.01.002. | **Quality and processes** |
| Ali SR, Dobbs TD, Mohamedbhai H, Whitaker S, Hutchings HA, Whitaker IS. Evaluating remote skin cancer multidisciplinary team meetings in the United Kingdom post-COVID-19. Journal of Plastic, Reconstructive & Aesthetic Surgery. 2023;84:250–7. https://doi.org/10.1016/j.bjps.2023.04.052. | **Virtual MDTs** |
| Alkasbi J, Mortuaire G, Rysman B, Nicot R, Chevalier D, Mouawad F. Match between therapeutic proposal in multidisciplinary tumor board and actual treatment in head and neck cancer. European Annals of Otorhinolaryngology, Head and Neck Diseases. 2021;138:247–52. https://doi.org/10.1016/j.anorl.2020.11.008. | **Adherence to recommendations** |
| Alsuhaibani RS, Alzahrani H, Algwaiz G, Alfarhan H, Alolayan A, Abdelhafiz N, et al. Enhancing the performance of gastrointestinal tumour board by improving documentation. BMJ Open Quality. 2018;7:e000168. https://doi.org/10.1136/bmjoq-2017-000168. | **Quality and processes** |
| Askelin B, Hind A, Paterson C. Exploring clinical decision-making among the uro-oncology multidisciplinary team: A qualitative study. Seminars in Oncology Nursing. 2023;39:151447. https://doi.org/10.1016/j.soncn.2023.151447. | **Decision-making** |
| Askelin B, Hind A, Paterson C. Exploring the impact of uro-oncology multidisciplinary team meetings on patient outcomes: A systematic review. European Journal of Oncology Nursing. 2021;54:102032. https://doi.org/10.1016/j.ejon.2021.102032. | **Systematic and scoping review** |
| Basta YL, Baur OL, van Dieren S, Klinkenbijl JHG, Fockens P, Tytgat KMAJ. Is there a benefit of multidisciplinary cancer team meetings for patients with gastrointestinal malignancies? Annals of Surgical Oncology. 2016;23:2430–7. https://doi.org/10.1245/s10434-016-5178-3. | **Adherence to recommendations** |
| Belda-Ferre M, Garcia-Segui A, Pacheco-Bru JJ, Valencia-Guadalajara VJ, Verdú-Verdú LP, Sánchez-Cano E, et al. Multicenter study on multidisciplinary committees in advanced prostate cancer. Actas Urologicas Espanolas. 2022;46:106–13. https://doi.org/10.1016/j.acuroe.2021.09.004. | **Quality and processes** |
| Berardi R, Morgese F, Rinaldi S, Torniai M, Mentrasti G, Scortichini L, et al. Benefits and limitations of a multidisciplinary approach in cancer patient management. Cancer Management and Research. 2020;12:9363–74. https://doi.org/10.2147/CMAR.S220976. | **Systematic and scoping review** |
| Bortot L, Targato G, Noto C, Giavarra M, Palmero L, Zara D, et al. Multidisciplinary team meeting proposal and final therapeutic choice in early breast cancer: Is there an agreement? Frontiers in Oncology. 2022;12:885992. https://doi.org/10.3389/fonc.2022.885992. | **Adherence to recommendations** |
| Brauer DG, Strand MS, Sanford DE, Kushnir VM, Lim K-H, Mullady DK, et al. Utility of a multidisciplinary tumor board in the management of pancreatic and upper gastrointestinal diseases: An observational study. HPB. 2017;19:133–9. https://doi.org/10.1016/j.hpb.2016.11.002. | **Patient management with teamwork** |
| Brims FJH, Kumarasamy C, Nash J, Leong TL, Stone E, Marshall HM. Hospital-based multidisciplinary lung cancer care in Australia: A survey of the landscape in 2021. BMJ Open Respiratory Research. 2022;9:e001157. https://doi.org/10.1136/bmjresp-2021-001157. | **Quality and processes** |
| Brink van den L, Ruiter AEC, Lagerveld BW, Graafland NM, Bex A, Beerlage HP, et al. The impact of a multidisciplinary tumor board (MTB) on treatment decision making for patients with renal cell carcinoma (RCC): 5-year data analysis. Clinical Genitourinary Cancer. 2024;22:610-617.e1. https://doi.org/10.1016/j.clgc.2024.01.021. | **Adherence to recommendations** |
| Brown GTF, Bekker HL, Young AL. Quality and efficacy of multidisciplinary team (MDT) quality assessment tools and discussion checklists: A systematic review. BMC Cancer. 2022;22:286. https://doi.org/10.1186/s12885-022-09369-8. | **Systematic and scoping review** |
| Cao Y, Mezzacappa C, Jaffe A, Strazzabosco M, Taddei TH. Adherence to tumor board recommendations in the treatment of patients with hepatocellular carcinoma. Journal of Multidisciplinary Healthcare. 2023;16:1531–40. https://doi.org/10.2147/JMDH.S407908. | **Adherence to recommendations** |
| Casadio M, Cardinale V, Klümpen H-J, Morement H, Lacasta A, Koerkamp BG, et al. Setup of multidisciplinary team discussions for patients with cholangiocarcinoma: Current practice and recommendations from the European Network for the Study of Cholangiocarcinoma (ENS-CCA). ESMO Open. 2022;7:100377. https://doi.org/10.1016/j.esmoop.2021.100377. | **Quality and processes** |
| De Leso PB, Coward JI, Letsa I, Schick U, Nandhabalan M, Frentzas S, et al. A study of the decision outcomes and financial costs of multidisciplinary team meetings (MDMs) in oncology. British Journal of Cancer. 2013;109:2295–300. https://doi.org/10.1038/bjc.2013.586. | **Resource requirements** |
| Dharmarajan H, Anderson JL, Kim S, Sridharan S, Duvvuri U, Ferris RL, et al. Transition to a virtual multidisciplinary tumor board during the COVID-19 pandemic: University of Pittsburgh experience. Head & Neck. 2020;42:1310–6. https://doi.org/10.1002/hed.26195. | **Virtual MDTs** |
| El Saghir NS, Charara RN, Kreidieh FY, Eaton V, Litvin K, Farhat RA, et al. Global practice and efficiency of multidisciplinary tumor boards: Results of an american society of clinical oncology international survey. Journal of Global Oncology. 2015;1:57–64. https://doi.org/10.1200/JGO.2015.000158. | **Patient management with teamwork** |
| Ernst J, Alfter K, Mustea A, Faridi A, Glowka T, Herrlinger U, et al. Improved implementation of tumor board decisions: A retrospective single center observational study in Germany. In Vivo. 2025;39:3503–15. https://doi.org/10.21873/invivo.14148. | **Adherence to recommendations** |
| Fehervari M, Hamrang-Yousefi S, Fadel MG, Mills SC, Warren OJ, Tekkis PP, et al. A systematic review of colorectal multidisciplinary team meetings: An international comparison. BJS Open. 2021;5:zrab044. https://doi.org/10.1093/bjsopen/zrab044. | **Systematic and scoping review** |
| Francisse S, Gkolfakis P, Viesca MFY, Mans L, Demols A, Pezzullo M, et al. The impact of a multidisciplinary team approach on the management of focal pancreatic lesions: A single tertiary center experience. Annals of Gastroenterology. 2023;36:580–7. https://doi.org/10.20524/aog.2023.0827. | **Patient management with teamwork** |
| Gandamihardja TAK, Soukup T, McInerney S, Green JSA, Sevdalis N. Analysing breast cancer multidisciplinary patient management: A prospective observational evaluation of team clinical decision-making. World Journal of Surgery. 2019;43:559–66. https://doi.org/10.1007/s00268-018-4815-3. | **Patient management with teamwork** |
| Gennheimer V, Akhoundova D, Hoffmann M, Jeker B, Banz Y, Bacher U, et al. Adherence to multidisciplinary tumor board recommendations in patients with multiple myeloma. Cancers. 2025;17:1297. https://doi.org/10.3390/cancers17081297. | **Adherence to recommendations** |
| Gouliaev A, Berg J, Bibi R, Arshad A, Leira HO, Neumann K, et al. Multi-disciplinary team meetings for lung cancer in Norway and Denmark: Results from national surveys and observations with MDT-MODe. Acta Oncologica. 2024;63:678–84. https://doi.org/10.2340/1651-226x.2024.40777. | **Quality and processes** |
| Groothuizen JE, Aroyewun E, Zasada M, Harris J, Hewish M, Taylor C. Virtually the same? Examining the impact of the COVID-19 related shift to virtual lung cancer multidisciplinary team meetings in the UK National Health Service: A mixed methods study. BMJ Open. 2023;13:e065494. https://doi.org/10.1136/bmjopen-2022-065494. | **Virtual MDTs** |
| Hahlweg P, Didi S, Kriston L, Härter M, Nestoriuc Y, Scholl I. Process quality of decision-making in multidisciplinary cancer team meetings: A structured observational study. BMC Cancer. 2017;17:772. https://doi.org/10.1186/s12885-017-3768-5. | **Decision-making** |
| Harris J, Green JS, Sevdalis N, Taylor C. Using peer observers to assess the quality of cancer multidisciplinary team meetings: A qualitative proof of concept study. Journal of Multidisciplinary Healthcare. 2014;7:355–63. https://doi.org/10.2147/JMDH.S65160. | **Quality and processes** |
| Hirth V, Schopow N, Pfränger J, Roschke E, Heyde C-E, Osterhoff G. Virtual versus in-person multidisciplinary musculoskeletal tumor conferences in times of COVID-19. Digital Health. 2023;9:1–5. https://doi.org/10.1177/20552076231179045. | **Virtual MDTs** |
| Hitz F, Ribi K, Grote G, Kolbe M, Schmitz C, Lamb BW, et al. Team functioning across different tumour types: Insights from a Swiss cancer center using qualitative and quantitative methods. Cancer Reports. 2022;5:e1541. https://doi.org/10.1002/cnr2.1541. | **Team performance** |
| Hollunder S, Herrlinger U, Zipfel M, Schmolders J, Janzen V, Thiesler T, et al. Cross-sectional increase of adherence to multidisciplinary tumor board decisions. BMC Cancer. 2018;18:936. https://doi.org/10.1186/s12885-018-4841-4. | **Adherence to recommendations** |
| Horlait M, Baes S, Regge M, Leys M. Understanding the complexity, underlying processes, and influencing factors for optimal multidisciplinary teamwork in hospital-based cancer teams: A systematic integrative review. Cancer Nursing. 2021;44:E476–92. https://doi.org/10.1097/NCC.0000000000000923. | **Systematic and scoping review** |
| Jalil R, Ahmed M, Green JSA, Sevdalis N. Factors that can make an impact on decision-making and decision implementation in cancer multidisciplinary teams: An interview study of the provider perspective. International Journal of Surgery. 2013;11:389–94. https://doi.org/10.1016/j.ijsu.2013.02.026. | **Decision-making** |
| Kandemir EA, Adam R, Roeper J, Ansmann L, Hülper P, Malik E, et al. Adherence to multidisciplinary tumor board (MTB) recommendations in patients with breast cancer: The results from two cancer centers in Germany. Clinical Breast Cancer. 2025;26:13–21. https://doi.org/10.1016/j.clbc.2025.10.010. | **Adherence to recommendations** |
| Kandemir EA, Roeper J, Zimmermann H, Ansmann L, Hülper P, Bockhorn M, et al. Adherence to multidisciplinary tumor board recommendations and its association with survival: A retrospective observational study of colorectal cancer patients. J Gastrointest Canc. 2025;56:124. https://doi.org/10.1007/s12029-025-01246-4. | **Adherence to recommendations** |
| Khassan T, Smitten E, Wood N, Fotopoulou C, Morrison J, MacDonald M, et al. MDT practice determines treatment pathway for patients with advanced ovarian cancer: A multi-centre observational study. European Journal of Surgical Oncology. 2023;49:1504–10. https://doi.org/10.1016/j.ejso.2023.03.210. | **Quality and processes** |
| Kočo L, Siebers CCN, Schlooz M, Meeuwis C, Oldenburg HSA, Prokop M, et al. Mapping current organizational structure and improvement points of breast cancer multidisciplinary team meetings - An interview study. Journal of Multidisciplinary Healthcare. 2022;15:2421–30. https://doi.org/10.2147/JMDH.S380293. | **Quality and processes** |
| Koo Y, Shafiq J, Yanga J, Avery S, Vinod SK. Quality of decision-making at oncology multidisciplinary team meetings: A structured observational study. Clinical Oncology. 2025;47:103942. https://doi.org/10.1016/j.clon.2025.103942. | **Decision-making** |
| Krause A, Stocker G, Gockel I, Seehofer D, Hoffmeister A, Bläker H, et al. Guideline adherence and implementation of tumor board therapy recommendations for patients with gastrointestinal cancer. Journal of Cancer Research and Clinical Oncology. 2023;149:1231–40. https://doi.org/10.1007/s00432-022-03991-6. | **Adherence to recommendations** |
| Lamb BW, Brown KF, Nagpal K, Vincent C, Green JSA, Sevdalis N. Quality of care management decisions by multidisciplinary cancer teams: A systematic review. Annals of Surgical Oncology. 2011;18:2116–25. https://doi.org/10.1245/s10434-011-1675-6. | **Systematic and scoping review** |
| Lamb BW, Jalil RT, Sevdalis N, Vincent C, Green JSA. Strategies to improve the efficiency and utility of multidisciplinary team meetings in urology cancer care: A survey study. BMC Health Services Research. 2014;14:377. https://doi.org/10.1186/1472-6963-14-377. | **Quality and processes** |
| Lamb BW, Sevdalis N, Taylor C, Vincent C, Green JSA. Multidisciplinary team working across different tumour types: Analysis of a national survey. Annals of Oncology. 2012;23:1293–300. https://doi.org/10.1093/annonc/mdr453. | **Quality and processes** |
| Luijten JCHBM, Westerman MJ, Nieuwenhuijzen GAP, Walraven JEW, Sosef MN, Beerepoot LV, et al. Team dynamics and clinician’s experience influence decision-making during Upper-GI multidisciplinary team meetings: A multiple case study. Frontiers in Oncology. 2022;12:1003506. https://doi.org/10.3389/fonc.2022.1003506. | **Decision-making** |
| Lumenta DB, Sendlhofer G, Pregartner G, Hart M, Tiefenbacher P, Kamolz LP, et al. Quality of teamwork in multidisciplinary cancer team meetings: A feasibility study. PloS ONE. 2019;14:e0212556. https://doi.org/10.1371/journal.pone.0212556. | **Decision-making** |
| Marrara B, Ibekwe O, Masika M, Attwood K, Gaudioso C, Nwogu C. Using an adapted tumor board evaluation tool for quality assessment of a thoracic multidisciplinary cancer conference: A pilot study. JCO Clinical Cancer Informatics. 2023;7:e2300017. https://doi.org/10.1200/CCI.23.00017. | **Decision-making** |
| Mohamedbhai H, Fernando S, Ubhi H, Chana S, Visavadia B. Advent of the virtual multidisciplinary team meeting: Do remote meetings work? The British Journal of Oral & Maxillofacial Surgery. 2021;59:1248–52. https://doi.org/10.1016/j.bjoms.2021.05.015. | **Virtual MDTs** |
| Mori S, Navarrete-Dechent C, Petukhova TA, Lee EH, Rossi AM, Postow MA, et al. Tumor board conferences for multidisciplinary skin cancer management: A survey of US cancer centers. Journal of the National Comprehensive Cancer Network. 2018;16:1209–15. https://doi.org/10.6004/jnccn.2018.7044. | **Patient management with teamwork** |
| Onifade A, Quaife SL, Holden D, Chung D, Birchall M, Peake MD, et al. Understanding the effectiveness and quality of virtual cancer multidisciplinary team meetings (MDTMs): A systematic scoping review. BMC Health Services Research. 2024;24:1481. https://doi.org/10.1186/s12913-024-11984-z. | **Systematic and scoping review** |
| Perlmutter B, Said SA-D, Hossain MS, Simon R, Joyce D, Walsh RM, et al. Lessons learned and keys to success: Provider experiences during the implementation of virtual oncology tumor boards in the era of COVID-19. Journal of Surgical Oncology. 2022;125:570–6. https://doi.org/10.1002/jso.26784. | **Virtual MDTs** |
| Petrella F, Radice D, Guarize J, Piperno G, Rampinelli C, Marinis F, et al. The impact of multidisciplinary team meetings on patient management in oncologic thoracic surgery: A single-center experience. Cancers. 2021;13:228. https://doi.org/10.3390/cancers13020228. | **Patient management with teamwork** |
| Pillay B, Wootten AC, Crowe H, Corcoran N, Tran B, Bowden P, et al. The impact of multidisciplinary team meetings on patient assessment, management and outcomes in oncology settings: A systematic review of the literature. Cancer Treatment Reviews. 2016;42:56–72. https://doi.org/10.1016/j.ctrv.2015.11.007. | **Systematic and scoping review** |
| Polomeni A, Bordessoule D, Malak S. Multidisciplinary team meetings in Hematology: A national mixed-methods study. BMC Cancer. 2023;23:950. https://doi.org/10.1186/s12885-023-11431-y. | **Quality and processes** |
| Prades J, Remue E, van Hoof E, Borras JM. Is it worth reorganising cancer services on the basis of multidisciplinary teams (MDTs)? A systematic review of the objectives and organisation of MDTs and their impact on patient outcomes. Health Policy. 2015;119:464–74. https://doi.org/10.1016/j.healthpol.2014.09.006. | **Systematic and scoping review** |
| Rehman AM, Naeem U, Rani A, Banatwala UESS, Salman A, Abdullah Khalid M, et al. How well does the virtual format of oncology multidisciplinary team meetings work? An assessment of participants’ perspectives and limitations: A scoping review. PloS ONE. 2023;18:e0294635. https://doi.org/10.1371/journal.pone.0294635. | **Systematic and scoping review** |
| Rosell L, Alexandersson N, Hagberg O, Nilbert M. Benefits, barriers and opinions on multidisciplinary team meetings: A survey in Swedish cancer care. BMC Health Services Research. 2018;18:249. https://doi.org/10.1186/s12913-018-2990-4. | **Decision-making** |
| Ruiz-Casado A, Ortega Solano MJ, Verdugo AS, Boado HC. Threats for sustainability of multidisciplinary working: Attitudes and perceptions of cancer care providers. Journal of Cancer Research and Therapeutics. 2018;14:1291–7. https://doi.org/10.4103/0973-1482.187293. | **Quality and processes** |
| Salami AC, Barden GM, Castillo DL, Hanna M, Petersen NJ, Davila JA, et al. Establishment of a regional virtual tumor board program to improve the process of care for patients with hepatocellular carcinoma. Journal of Oncology Practice. 2015;11:e66-74. https://doi.org/10.1200/JOP.2014.000679. | **Virtual MDTs** |
| Sassé B, Shaya S, Nimmo J, Cao K, Day D, Evans K, et al. Evaluating the impact of a tertiary multidisciplinary meeting in metastatic breast cancer: A prospective study. The Breast. 2025;79:103861. https://doi.org/10.1016/j.breast.2024.103861. | **Resource requirements** |
| Schäfer N, Bumes E, Eberle F, Fox V, Gessler F, Giordano FA, et al. Implementation, relevance, and virtual adaptation of neuro-oncological tumor boards during the COVID-19 pandemic: A nationwide provider survey. Journal of Neuro-Oncology. 2021;153:479–85. https://doi.org/10.1007/s11060-021-03784-w. | **Virtual MDTs** |
| Scott R, Hawarden A, Russell B, Edmondson RJ. Decision-making in gynaecological oncology multidisciplinary team meetings: A cross-sectional, observational study of ovarian cancer cases. Oncology Research and Treatment. 2020;43:70–7. https://doi.org/10.1159/000504260. | **Decision-making** |
| Seretis C, Mankotia R, Goonetilleke K, Rawstorne, Edward. Quality assessment of decision–making in colorectal cancer multidisciplinary meetings. JBUON. 2014;19:913–6. | **Quality and processes** |
| Soukup T, Gandamihardja TAK, McInerney S, Green JSA, Sevdalis N. Do multidisciplinary cancer care teams suffer decision-making fatigue: An observational, longitudinal team improvement study. BMJ Open. 2019;9:e027303. https://doi.org/10.1136/bmjopen-2018-027303. | **Decision-making** |
| Soukup T, Lamb BW, Green JSA, Sevdalis N, Murtagh G. Analysis of communication styles underpinning clinical decision-making in cancer multidisciplinary team meetings. Frontiers in Psychology. 2023;14:1105235. https://doi.org/10.3389/fpsyg.2023.1105235. | **Team performance** |
| Soukup T, Lamb BW, Morbi A, Shah NJ, Bali A, Asher V, et al. A multicentre cross-sectional observational study of cancer multidisciplinary teams: Analysis of team decision making. Cancer Medicine. 2020;9:7083–99. https://doi.org/10.1002/cam4.3366. | **Decision-making** |
| Soukup T, Lamb BW, Morbi A, Shah NJ, Bali A, Asher V, et al. Cancer multidisciplinary team meetings: Impact of logistical challenges on communication and decision-making. BJS Open. 2022;6:zrac093. https://doi.org/10.1093/bjsopen/zrac093. | **Team performance** |
| Soukup T, Lamb BW, Sarkar S, Arora S, Shah S, Darzi A, et al. Predictors of treatment decisions in multidisciplinary oncology meetings: A quantitative observational study. Annals of Surgical Oncology. 2016;23:4410–7. https://doi.org/10.1245/s10434-016-5347-4. | **Decision-making** |
| Soukup T, Murtagh G, Lamb BW, Green JSA, Sevdalis N. Degrees of multidisciplinarity underpinning care planning for patients with cancer in weekly multidisciplinary team meetings: Conversation analysis. Journal of Multidisciplinary Healthcare. 2021;14:411–24. https://doi.org/10.2147/JMDH.S270394. | **Quality and processes** |
| Soukup T, Petrides KV, Lamb BW, Sarkar S, Arora S, Shah S, et al. The anatomy of clinical decision-making in multidisciplinary cancer meetings: A cross-sectional observational study of teams in a natural context. Medicine. 2016;95:e3885. https://doi.org/10.1097/MD.0000000000003885. | **Decision-making** |
| Soukup T, Winters D, Chua K-C, Rowland P, Moneke J, Skolarus TA, et al. Evaluation of changes to work patterns in multidisciplinary cancer team meetings due to the COVID-19 pandemic: A national mixed-method survey study. Cancer Medicine. 2023;12:8729–41. https://doi.org/10.1002/cam4.5608. | **Quality and processes** |
| Taylor C, Atkins L, Richardson A, Tarrant R, Ramirez A-J. Measuring the quality of MDT working: An observational approach. BMC Cancer. 2012;12:202. https://doi.org/10.1186/1471-2407-12-202. | **Quality and processes** |
| Taylor C, Harris J, Stenner K, Sevdalis N, Green SAJ. A multi-method evaluation of the implementation of a cancer teamwork assessment and feedback improvement programme (MDT-FIT) across a large integrated cancer system. Cancer Medicine. 2021;10:1240–52. https://doi.org/10.1002/cam4.3719. | **Decision-making** |
| Tullis JA. Tumor talk: A descriptive study of communication about tumor board meetings. Qualitative Research in Medicine & Healthcare. 2022;6:10717. https://doi.org/10.4081/qrmh.2022.10717. | **Team performance** |
| van Huizen LS, Dijkstra PU, Hemmer PHJ, van Etten B, Buis CI, Olsder L, et al. Reorganizing the multidisciplinary team meetings in a tertiary centre for gastro-intestinal oncology adds value to the internal and regional care pathways. A mixed method evaluation. International Journal of Integrated Care. 2021;21:1–15. https://doi.org/10.5334/ijic.5526. | **Virtual MDTs** |
| Vinod SK, Wellege NT, Kim S, Duggan KJ, Ibrahim M, Shafiq J. Translation of oncology multidisciplinary team meeting (MDM) recommendations into clinical practice. BMC Health Services Research. 2021;21:461. https://doi.org/10.1186/s12913-021-06511-3. | **Adherence to recommendations** |
| Walraven JEW, van der Hel OL, van der Hoeven JJM, Lemmens VEPP, Verhoeven RHA, Desar IME. Factors influencing the quality and functioning of oncological multidisciplinary team meetings: Results of a systematic review. BMC Health Services Research. 2022;22:829. https://doi.org/10.1186/s12913-022-08112-0. | **Systematic and scoping review** |
| Walraven JEW, Verhoeven RHA, van der Meulen R, van der Hoeven JJM, Lemmens VEPP, Hesselink G, et al. Facilitators and barriers to conducting an efficient, competent and high-quality oncological multidisciplinary team meeting. BMJ Open Quality. 2023;12:e002130. https://doi.org/10.1136/bmjoq-2022-002130. | **Team performance** |
| Warner R, Hoinville L, Pottle E, Taylor C, Green J. Refocusing cancer multidisciplinary team meetings in the United Kingdom: Comparing urology with other specialties. Annals of the Royal College of Surgeons of England. 2021;103:10–7. https://doi.org/10.1308/rcsann.2020.0212. | **Quality and processes** |
| Wihl J, Rosell L, Carlsson T, Kinhult S, Lindell G, Nilbert M. Medical and nonmedical information during multidisciplinary team meetings in cancer care. Current Oncology. 2021;28:1008–16. https://doi.org/10.3390/curroncol28010098. | **Decision-making** |
| Wihl J, Rosell L, Frederiksen K, Kinhult S, Lindell G, Nilbert M. Contributions to multidisciplinary team meetings in cancer care: Predictors of complete case information and comprehensive case discussions. Journal of Multidisciplinary Healthcare. 2021;14:2445–52. https://doi.org/10.2147/JMDH.S309162. | **Quality and processes** |
| Zasada M, Harris J, Groothuizen J, Aroyewun E, Mendis J, Taylor C, et al. Investigating the efficiency of lung multi-disciplinary team meetings-A mixed methods study of eight lung multi-disciplinary teams. Cancer Medicine. 2023;12:9999–10007. https://doi.org/10.1002/cam4.5730. | **Quality and processes** |

**Excluded with reason – systematic search**

| **References** | **Exclusion criteria** |
| --- | --- |
| Aggarwal G, Roy MK. Mult+B3:C249idisciplinary team meetings for optimal management of cancer patients: A must? Indian Journal of Cancer. 2014;51:495. https://doi.org/10.4103/0019-509X.175337. | **Editorial, study design** |
| Abdullayev N, Kottlors J, Habibov H, Yilmaz F, Zimmer C, Hokamp NG, et al. European guideline informed RAG-based GPT-4 decision support tool in tumor board meetings for breast cancer treatment. European Journal of Surgical Oncology. 2025;51:110384. https://doi.org/10.1016/j.ejso.2025.110384. | **Technical support system/AI** |
| Abuelgasim KA, Jazieh AR. Quality measures for multidisciplinary tumor boards and their role in improving cancer care. Global Journal on Quality and Safety in Healthcare. 2024;7:28–33. https://doi.org/10.36401/JQSH-23-22. | **No systematic review and scoping review** |
| Aghamaliyev U, Karimbayli J, Giessen-Jung C, Ilmer M, Unger K, Andrade D, et al. ChatGPT’s gastrointestinal tumor board tango: A limping dance partner? European Journal of Cancer. 2024;205:114100. https://doi.org/10.1016/j.ejca.2024.114100. | **Technical support system/AI** |
| Alami K, Willemse E, Quiriny M, Lipski S, Laurent C, Donquier V, et al. Evaluation of ChatGPT-4’s performance in therapeutic decision-making during multidisciplinary oncology meetings for head and neck squamous cell carcinoma. Cureus. 2024;16:e68808. https://doi.org/10.7759/cureus.68808. | **Technical support system/AI** |
| Alfieri S, Brunelli C, Borreani C, Capri G, Angi M, Bianchi GV, et al. Characterizing different multidisciplinary team models implemented within one comprehensive cancer center. Journal of Multidisciplinary Healthcare. 2023;16:1845–55. https://doi.org/10.2147/JMDH.S402348. | **Topic** |
| Algwaiz G, Salam Y, Bustami R, Ferwana M, Jazieh AR. Do multidisciplinary tumor board discussions correlate with increase in 5-Year survival? A meta-analysis study. Global Journal on Quality and Safety in Healthcare. 2021;4:3–10. https://doi.org/10.36401/JQSH-20-23. | **Patient management without teamwork** |
| Al-Hammouri T, Almeida-Magana R, Soukup T, Lamb BW. Implementation of streamlining measures in selecting and prioritising complex cases for the cancer multidisciplinary team meeting: A mini review of the recent developments. Frontiers in Health Services. 2024;4:1340320. https://doi.org/10.3389/frhs.2024.1340320. | **Streamlining** |
| Alkhathlan A, Alfaiz R, Almusallam G, Arabi E, Alkaiyat M, Jazieh AR. Improving coordination of lung cancer care at a tertiary healthcare center in Saudi Arabia. Saudi Medical Journal. 2022;43:313–6. https://doi.org/10.15537/smj.2022.43.3.20210750. | **Patient management without teamwork** |
| Alterio D, Preda L, Volpe S, Giannitto C, Riva G, Kamga Pounou FA, et al. Impact of a dedicated radiologist as a member of the head and neck tumour board: A single-institution experience. ACTA Otorhinolaryngologica Italica. 2020;40:26–32. https://doi.org/10.14639/0392-100X-N0326. | **Profession** |
| Amin NB, Bridgham KM, Brown JP, Moyer KF, Taylor RJ, Wolf JS, et al. Regionalization of head and neck oncology tumor boards: Perspectives of collaborating physicians. OTO Open. 2023;7:e18. https://doi.org/10.1002/oto2.18. | **Sample** |
| Ammo T, Guillaume VGJ, Hofmann UK, Ulmer NM, Buenting N, Laenger F, et al. Evaluating ChatGPT-4o as a decision support tool in multidisciplinary sarcoma tumor boards: Heterogeneous performance across various specialties. Frontiers in Oncology. 2025;14:1526288. https://doi.org/10.3389/fonc.2024.1526288. | **Technical support system/AI** |
| Andrew TW, Hamnett N, Roy I, Garioch J, Nobes J, Moncrieff MD. Machine-learning algorithm to predict multidisciplinary team treatment recommendations in the management of basal cell carcinoma. British Journal of Cancer. 2022;126:562–8. https://doi.org/10.1038/s41416-021-01506-7. | **Technical support system/AI** |
| Angel M, Demiray M, Dişel U, Passos J. The value of virtual molecular tumor boards for informed clinical decision-making. Oncologist. 2024;29:554–9. https://doi.org/10.1093/oncolo/oyae077. | **Rare diseases** |
| Ballatore Z, Bozzi F, Cardea S, Savino FD, Migliore A, Tarantino V, et al. Molecular tumour board (MTB): From standard therapy to precision medicine. Journal of Clinical Medicine. 2023;12:6666. https://doi.org/10.3390/jcm12206666. | **Rare diseases** |
| Basendowah M, Awlia AM, Alamoudi HA, Ali Kanawi HM, Saleem A, Malibary N, et al. Impact of optional multidisciplinary tumor board meeting on the mortality of patients with gastrointestinal cancer: A retrospective observational study. Cancer Reports. 2021;4:e1373. https://doi.org/10.1002/cnr2.1373. | **Patient management without teamwork** |
| Basta YL, Bolle S, Fockens P, Tytgat KMAJ. The value of multidisciplinary team meetings for patients with gastrointestinal malignancies: A systematic review. Annals of Surgical Oncology. 2017;24:2669–78. https://doi.org/10.1245/s10434-017-5833-3. | **Patient management without teamwork** |
| Bednarski BK, Taggart M, Chang GJ. MDT-How it is important in rectal cancer. Abdominal Radiology. 2023;48:2807–13. https://doi.org/10.1007/s00261-023-03977-z. | **No study** |
| Behel V, Noronha V, Choughule A, Shetty O, Chandrani P, Kapoor A Bondili, Suresh Kumar, et al. Impact of molecular tumor board on the clinical management of patients with cancer. JCO Global Oncology. 2022;8:e2200030. | **Rare diseases** |
| Bellardita L, Donegani S, Spatuzzi AL, Valdagni R. Multidisciplinary versus one-on-one setting: A qualitative study of clinicians’ perceptions of their relationship with patients with prostate cancer. Journal of Oncology Practice. 2011;7:e1–5. https://doi.org/10.1200/JOP.2010.000020. | **No MDT** |
| Berger Y, Buseck A, Imtiaz S, Horn C, Khajoueinejad N, Macfie R, et al. Actual and perceived gender differences in virtual tumor board participation. Surgery Open Science. 2023;16:28–32. https://doi.org/10.1016/j.sopen.2023.09.004. | **Topic** |
| Bhutiani N, Peacock O, Uppal A, You YN, Bednarski BK, Skibber JM, et al. The current multidisciplinary management of rectal cancer. Annals of Gastroenterological Surgery. 2024;8:394–400. https://doi.org/10.1002/ags3.12777. | **Medical outcome** |
| Boisen AS, Balslev, Natasja Espeløv. Multidisciplinære team i kræftbehandlingen. Ugeskr Læger. 2016;178:V10150792. | **Language** |
| Bonanno N, Cioni D, Caruso D, Cyran CC, Dinkel J, Fournier L, et al. Attitudes and perceptions of radiologists towards online (virtual) oncologic multidisciplinary team meetings during the COVID-19 pandemic - A survey of the European Society of Oncologic Imaging (ESOI). European Radiology. 2023;33:1194–204. https://doi.org/10.1007/s00330-022-09083-w. | **Profession** |
| Boniface MM, Wani SB, Schefter TE, Koo PJ, Meguid C, Leong S, et al. Multidisciplinary management for esophageal and gastric cancer. Cancer Management and Research. 2016;8:39–44. https://doi.org/10.2147/CMAR.S101169. | **Topic** |
| Booth ME, Jones CM, Helbrow J, Mansoor W, Peters CJ, Petty RD, et al. The UK national oesophagogastric multidisciplinary team meeting: An initiative from the UK & Ireland oesophagogastric group. Clinical Oncology. 2023;35:417–20. https://doi.org/10.1016/j.clon.2023.03.017. | **Editorial, study design** |
| Boxer MM, Duggan KJ, Descallar J, Vinod SK. Do patients discussed at a lung cancer multidisciplinary team meeting receive guideline-recommended treatment? Asia-Pacific Journal of Clinical Oncology. 2016;12:52–60. https://doi.org/10.1111/ajco.12421. | **Topic** |
| Boxer MM, Vinod SK, Shafiq J, Duggan KJ. Do multidisciplinary team meetings make a difference in the management of lung cancer? Cancer. 2011;117:5112–20. https://doi.org/10.1002/cncr.26149. | **Patient management without teamwork** |
| Bräuner KB, Mashkoor M, Gögenur M, Lin V, Oppermann C, Gögenur I. The association between conducting a multidisciplinary team conference and short- and long-term outcomes after colorectal cancer surgery: A national register study. Ann Surg Oncol. 2025;32:9491–501. https://doi.org/10.1245/s10434-025-18353-y. | **Medical outcome** |
| Brne K, Abraham K, Arnoldus M, Romijnders K, Uršič Bensa Š, Trappenburg J. Digital tumour board solution enhances case preparation time and reduces postponements: an implementer report. BMJ Health Care Inform. 2025;32:e101332. https://doi.org/10.1136/bmjhci-2024-101332. | **Technical support system/AI** |
| Brown PJ, Rossington H, Taylor J, Lambregts DMJ, Morris EJA, West NP, et al. Radiologist and multidisciplinary team clinician opinions on the quality of MRI rectal cancer staging reports: How are we doing? Clinical Radiology. 2019;74:637–42. https://doi.org/10.1016/j.crad.2019.04.015. | **Topic** |
| Brugel M, Carlier C, Essner C, Debreuve-Theresette A, Beck M-F, Merrouche Y, et al. Dramatic changes in oncology care pathways during the COVID-19 pandemic: The french ONCOCARE-COV study. The Oncologist. 2021;26:e338–41. https://doi.org/10.1002/onco.13578. | **Topic** |
| Campbell BA, Ball D, Mornex F. Multidisciplinary lung cancer meetings: Improving the practice of radiation oncology and facing future challenges. Respirology. 2015;20:192–8. https://doi.org/10.1111/resp.12459. | **Profession** |
| Castro G, Souza FH, Lima J, Bernardi LP, Teixeira CHA, Prado GF. Does multidisciplinary team management improve clinical outcomes in NSCLC? A systematic review with meta-analysis. JTO Clinical and Research Reports. 2023;4:100580. https://doi.org/10.1016/j.jtocrr.2023.100580. | **Medical outcome** |
| Cathcart P, Smith S, Clayton G. Strengths and limitations of video-conference multidisciplinary management of breast disease during the COVID-19 pandemic. The British Journal of Surgery. 2021;108:e20–1. https://doi.org/10.1093/bjs/znaa046. | **Editorial, study design** |
| Chae BJ, Bae JS, Song BJ, Jung SS. Multidisciplinary team approach in breast cancer: A nationwide survey in Korea. Journal of the Korean Surgical Society. 2012;82:340–6. https://doi.org/10.4174/jkss.2012.82.6.340. | **Sample** |
| Chambers AJ, Enoch JF, Wong J, Spigelman A. When teams disagree: Investigating the incidence and causes of dissent occurring in cancer multidisciplinary team meetings. Asia-Pacific Journal of Clinical Oncology. 2024;20:234–9. https://doi.org/10.1111/ajco.13919. | **Topic** |
| Charara RN, Kreidieh FY, Farhat RA, Al-Feghali KA, Khoury KE, Haydar A, et al. Practice and impact of multidisciplinary tumor boards on patient management: A prospective study. Journal of Global Oncology. 2017;3:242–9. https://doi.org/10.1200/JGO.2016.004960. | **Middle- and low-income countries** |
| Charo LM, Eskander RN, Sicklick J, Kim KH, Lim HJ, Okamura R, et al. Real-world data from a molecular tumor board: Improved outcomes in breast and gynecologic cancers patients with precision medicine. JCO Precision Oncology. 2022;6:e2000508. | **Rare diseases** |
| Choi DT, Sada YH, Sansgiry S, Kaplan DE, Taddei TH, Aguilar JK, et al. Using telemedicine to facilitate patient communication and treatment decision-making following multidisciplinary tumor board review for patients with hepatocellular carcinoma. Journal of Gastrointestinal Cancer. 2023;54:623–31. https://doi.org/10.1007/s12029-022-00844-w. | **Topic** |
| Choi HH, Filice RW. Streamlining radiologist workflow for multidisciplinary conferences: A web-based system to represent radiology. Journal of Digital Imaging. 2020;33:602–6. https://doi.org/10.1007/s10278-019-00317-w. | **Technical support system/AI** |
| Christ SM, Heesen P, Muehlematter UJ, Pohl K, William Thiel G, Willmann J, et al. Recognition of and treatment recommendations for oligometastatic disease in multidisciplinary tumor boards. Clinical and Translational Radiation Oncology. 2023;38:123–9. https://doi.org/10.1016/j.ctro.2022.11.008. | **Medical outcome** |
| Creemers SG, van Santvoort B, van den Berkmortel FWPJ, Kiemeney LA, van Oort IM, Aben KKH, et al. Role of multidisciplinary team meetings in implementation of chemohormonal therapy in metastatic prostate cancer in daily practice. Prostate Cancer and Prostatic Diseases. 2023;26:133–41. https://doi.org/10.1038/s41391-022-00556-z. | **No full text** |
| Croke JM, El-Sayed S. Multidisciplinary management of cancer patients: Chasing a shadow or real value? An overview of the literature. Current Oncology. 2012;19:e232–8. https://doi.org/10.3747/co.19.944. | **Patient management without teamwork** |
| Currie GP, Kennedy A-M, Chetty M. COVID-19 and the multidisciplinary team meeting: “Should old acquaintance be forgot?” Journal of the Royal College of Physicians of Edinburgh. 2021;51:327–9. https://doi.org/10.4997/JRCPE.2021.402. | **Editorial, study design** |
| Daubisse-Marliac L, Biboulet M, Delpierre C, Rivera P, Bauvin É, Grosclaude P. Exhaustivité et qualité des réunions de concertation pluridisciplinaire: l’exemple du cancer du sein dans le département du Tarn. Bulletin du Cancer. 2012;99:815–26. https://doi.org/10.1684/bdc.2012.1622. | **Language** |
| de Luca E, Sena B. Searching for a professional identity: A qualitative study of the oncology nurses role in a multidisciplinary breast-unit team. Acta Biomedica. 2021;92:e2021506. https://doi.org/10.23750/abm.v92iS2.11643. | **Profession** |
| Dermine S, Barret M, Prieux C, Ribière S, Leblanc S, Dhooge M, et al. Impact of a dedicated multidisciplinary meeting on the management of superficial cancers of the digestive tract. Endoscopy International Open. 2018;6:E1470–6. https://doi.org/10.1055/a-0658-1350. | **Patient management without teamwork** |
| Di Pilla A, Cozzolino MR, Mannocci A, Carini E, Spina F, Castrini F, et al. The impact of tumor boards on breast cancer care: Evidence from a systematic literature review and meta-analysis. International Journal of Environmental Research and Public Health. 2022;19:14990. https://doi.org/10.3390/ijerph192214990. | **Patient management without teamwork** |
| Dickhoff C, Dahele M. The multidisciplinary lung cancer team meeting: Increasing evidence that it should be considered a medical intervention in its own right. Journal of Thoracic Disease. 2019;11 Suppl 3:S311–4. https://doi.org/10.21037/jtd.2019.01.14. | **Editorial, study design** |
| Dogan I, Bartin MK, Sonmez E, Seyran E, Bozkurt HA, Yuksek M, et al. Chat GPT performance in multi-disciplinary boards—Should AI be a member of cancer boards? Healthcare. 2025;13:2254. https://doi.org/10.3390/healthcare13182254. | **Technical support system/AI** |
| Du C-Z, Li J, Cai Y, Sun Y-S, Xue W-C, Gu J. Effect of multidisciplinary team treatment on outcomes of patients with gastrointestinal malignancy. World Journal of Gastroenterology. 2011;17:2013–8. https://doi.org/10.3748/wjg.v17.i15.2013. | **Patient management without teamwork** |
| Ebben KCWJ, Hendriks MP, Markus L, Kos M, de Hingh IHJT, Oddens JR, et al. Using guideline-based clinical decision support in oncological multidisciplinary team meetings: A prospective, multicenter concordance study. International Journal for Quality in Health Care. 2022;34:1–12. https://doi.org/10.1093/intqhc/mzac007. | **Technical support system/AI** |
| Eichler M, Andreou D, Golcher H, Hentschel L, Richter S, Hohenberger P, et al. Utilization of interdisciplinary tumor boards for sarcoma care in Germany: Results from the PROSa study. Oncology Research and Treatment. 2021;44:301–12. https://doi.org/10.1159/000516262. | **Rare diseases** |
| El Saghir NS, El-Asmar N, Hajj C, Eid T, Khatib S, Bounedjar A, et al. Survey of utilization of multidisciplinary management tumor boards in Arab countries. Breast. 2011;20:S70–4. https://doi.org/10.1016/j.breast.2011.01.011. | **Middle- and low-income countries** |
| El-Shabrawi K, Burkhardt V, Becker C. Impact of a multidisciplinary head and neck tumor board on treatment and survival in laryngeal carcinoma. Current Oncology. 2023;30:10085–99. https://doi.org/10.3390/curroncol30120733. | **Patient management without teamwork** |
| Engelhardt M, Ihorst G, Schumacher M, Rassner M, Gengenbach L, Möller M, et al. Multidisciplinary tumor boards and their analyses: The yin and yang of outcome measures. BMC Cancer. 2021;21:173. https://doi.org/10.1186/s12885-021-07878-6. | **Medical outcome** |
| Engstrand J, Kartalis N, Strömberg C, Broberg M, Stillström A, Lekberg T, et al. The impact of a hepatobiliary multidisciplinary team assessment in patients with colorectal cancer liver metastases: A population-based study. The Oncologist. 2017;22:1067–74. https://doi.org/10.1634/theoncologist.2017-0028. | **Patient management without teamwork** |
| Erdat EC, Yalçıner M, Örüncü MB, Ürün Y, Şenler FÇ. Assessing the accuracy of the GPT-4 model in multidisciplinary tumor board decision prediction. Clin Transl Oncol. 2025;27:3793–802. https://doi.org/10.1007/s12094-025-03905-1. | **Technical support system/AI** |
| Esteso F, Tissera NS, O’Connor JM, Luca R, Huertas E, Sánchez Loria F, et al. Implementation of a virtual multicenter gastrointestinal tumor board to reduce cancer disparities in Argentina. World Journal of Clinical Oncology. 2022;13:423–8. https://doi.org/10.5306/wjco.v13.i6.423. | **Topic** |
| Fassler C, Yalamanchi P, Aweeda M, Rezk J, Murphy B, Lockney NA, et al. Visual pathology reports for improved collaboration at multidisciplinary head and neck tumor board. Head Neck. 2025;47:452–62. https://doi.org/10.1002/hed.27926. | **Profession** |
| Fenton ME, Wade SA, Pirrili BN, Balogh ZJ, Rowe CW, Bendinelli C. Variability in thyroid cancer multidisciplinary team meeting recommendations is not explained by standard variables: Outcomes of a single centre review. Journal of Clinical Medicine. 2021;10. https://doi.org/10.3390/jcm10184150. | **Medical outcome** |
| Ferro A, Cristofolini P, Garcia-Etienne CA, Caffo O, Pellegrini M, Fantò C, et al. Learning from organisational changes in the management of breast cancer patients during the COVID-19 pandemic: Preparing for a second wave at a breast unit in northern Italy. The International Journal of Health Planning and Management. 2021;36:1030–7. https://doi.org/10.1002/hpm.3181. | **Topic** |
| Foster TJ, Bouchard-Fortier A, Olivotto IA, Quan ML. Effect of multidisciplinary case conferences on physician decision making: Breast diagnostic rounds. Cureus. 2016;8:e895. https://doi.org/10.7759/cureus.895. | **Patient management without teamwork** |
| Fradgley EA, Booth K, Paul C, Zdenkowski N, Rankin NM. Facilitating high quality cancer care: A qualitative study of Australian chairpersons’ perspectives on multidisciplinary team meetings. Journal of Multidisciplinary Healthcare. 2021;14:3429–39. https://doi.org/10.2147/JMDH.S332972. | **Profession** |
| Freytag M, Herrlinger U, Hauser S, Bauernfeind FG, Gonzalez-Carmona MA, Landsberg J, et al. Higher number of multidisciplinary tumor board meetings per case leads to improved clinical outcome. BMC Cancer. 2020;20:355. https://doi.org/10.1186/s12885-020-06809-1. | **Number of MDTs, no teamwork** |
| Gallard C, Dinulescu M, Droitcourt C, Boussemart L, Adamski H, Rousseau C, et al. Cancers cutanés de la face: avis comparés de réunions de concertation pluridisciplinaire françaises. Annales de Dermatologie et de Vénéréologie. 2020;147:179–87. https://doi.org/10.1016/j.annder.2019.09.614. | **Language** |
| Garcia D, Spruill LS, Irshad A, Wood J, Kepecs D, Klauber-DeMore N. The value of a second opinion for breast cancer patients referred to a National Cancer Institute (NCI)-Designated cancer center with a multidisciplinary breast tumor board. Annals of Surgical Oncology. 2018;25:2953–7. https://doi.org/10.1245/s10434-018-6599-y. | **Patient management without teamwork** |
| Gaudino S, Giordano C, Magnani F, Cottonaro S, Infante A, Sabatino G, et al. Neuro-oncology multidisciplinary tumor board: The point of view of the neuroradiologist. Journal of Personalized Medicine. 2022;12:135. https://doi.org/10.3390/jpm12020135. | **Profession** |
| Gebbia V, Guarini A, Piazza D, Bertani A, Spada M, Verderame F, et al. Virtual multidisciplinary tumor boards: A narrative review focused on lung cancer. Pulmonary Therapy. 2021;7:295–308. https://doi.org/10.1007/s41030-021-00163-8. | **Rare diseases** |
| Geerts PAF, van der Weijden T, Savelberg W, Altan M, Chisari G, Launert DR, et al. The next step toward patient-centeredness in multidisciplinary cancer team meetings: An interview study with professionals. Journal of Multidisciplinary Healthcare. 2021;14:1311–24. https://doi.org/10.2147/JMDH.S286044. | **Patient involvement** |
| Gherman A, Andrei D, Popoiu CM, Stoicescu ER, Levai MC, Stoian II, et al. Multidisciplinary telemedicine in healthcare during and after the COVID-19 pandemic: A narrative review. Life. 2025;15:783. https://doi.org/10.3390/life15050783. | **No systematic review and scoping review** |
| Grosclaude P, Azria D, Guimbaud R, Thibault S, Daubisse-Marliac L, Cartron G, et al. Impact du SARS-CoV2 sur la structuration de la prise en charge du cancer: exemple de la tenue des RCP de cancérologie en Occitanie. Bulletin du Cancer. 2020;107:730–7. https://doi.org/10.1016/j.bulcan.2020.05.001. | **Language** |
| Guillem P, Bolla M, Courby S, Descotes J-L, Laramas M, Moro-Sibilot D. Évaluation des réunions de concertation pluridisciplinaire en cancérologie: quelles priorités pour quelles améliorations? Bulletin du Cancer. 2011;98:989–98. https://doi.org/10.1684/bdc.2011.1428. | **Language** |
| Guirado M, Sanchez-Hernandez A, Pijuan L, Teixido C, Gómez-Caamaño A, Cilleruelo-Ramos Á. Quality indicators and excellence requirements for a multidisciplinary lung cancer tumor board by the Spanish Lung Cancer Group. Clinical & Translational Oncology. 2022;24:446–59. https://doi.org/10.1007/s12094-021-02712-8. | **No systematic review and scoping review** |
| Güler SA, Cantürk NZ. Multidisciplinary breast cancer teams and proposed standards. Turkish Journal of Surgery. 2015;31:39–41. https://doi.org/10.5152/UCD.2014.2724. | **Topic** |
| Guy J-B, Benna M, Xia Y, Daguenet E, Ben Mrad M, Jmour O, et al. Quality insurance in head and neck cancer multidisciplinary team meetings: A watchful eye on real-life experience. Oral Oncology. 2019;91:35–8. https://doi.org/10.1016/j.oraloncology.2019.02.020. | **Medical outcome** |
| Habermann TM, Khurana A, Lentz R, Schmitz JJ, Bormann AG, Young JR, et al. Analysis and impact of a multidisciplinary lymphoma virtual tumor board. Leukemia & Lymphoma. 2020;61:3351–9. https://doi.org/10.1080/10428194.2020.1817432. | **Patient management without teamwork** |
| Haddad P, Mir M-R, Jamali M, Abdirad A. Gastrointestinal tumor board: An evolving experience in tehran cancer institute. Acta Medica Iranica. 2013;51:271–3. | **Middle- and low-income countries** |
| Hahlweg P, Hoffmann J, Härter M, Frosch DL, Elwyn G, Scholl I. In absentia: An exploratory study of how patients are considered in multidisciplinary cancer team meetings. PloS One. 2015;10:e0139921. https://doi.org/10.1371/journal.pone.0139921. | **Patient involvement** |
| Hamilton DW, Heaven B, Thomson R, Wilson J, Exley C. How do patients make decisions in the context of a multidisciplinary team: An ethnographic study of four head and neck cancer centres in the north of England. BMJ Open. 2022;12:e061654. https://doi.org/10.1136/bmjopen-2022-061654. | **Patient involvement** |
| Hamilton DW, Heaven B, Thomson RG, Wilson JA, Exley C. Multidisciplinary team decision-making in cancer and the absent patient: A qualitative study. BMJ Open. 2016;6:e012559. https://doi.org/10.1136/bmjopen-2016-012559. | **Patient involvement** |
| Hammer RD, Fowler D, Sheets LR, Siadimas A, Guo C, Prime MS. Digital tumor board solutions have significant impact on case preparation. JCO Clinical Cancer Informatics. 2020;4:757–68. | **Technical support system/AI** |
| Harris J, Taylor C, Sevdalis N, Jalil R, Green JSA. Development and testing of the cancer multidisciplinary team meeting observational tool (MDT-MOT). International Journal for Quality in Health Care. 2016;28:332–8. https://doi.org/10.1093/intqhc/mzw030. | **Validation** |
| Harzstark AL, Altschuler A, Amsden LB, Alavi M, Liu L, Presti JC, et al. Implementation of a multidisciplinary expert testicular cancer tumor board across a large integrated healthcare delivery system via early case ascertainment. JCO Clinical Cancer Informatics. 2021;5:187–93. | **Topic** |
| He C. Multidisciplinary team meetings: Barriers to implementation in cancer care. Oncology (Williston Park). 2024;38:339–44. https://doi.org/10.46883/2024.25921026. | **No systematic review and scoping review** |
| Heinke MY, Vinod SK. A review on the impact of lung cancer multidisciplinary care on patient outcomes. Translational Lung Cancer Research. 2020;9:1639–53. https://doi.org/10.21037/tlcr.2019.11.03. | **Patient management without teamwork** |
| Hendrickx J-J, Mennega T, Uppelschoten JM, Leemans CR. Changes in multidisciplinary team decisions in a high volume head and neck oncological center following those made in its preferred partner. Frontiers in Oncology. 2023;13:1205224. https://doi.org/10.3389/fonc.2023.1205224. | **Patient management without teamwork** |
| Hendriks MP, Jager A, Ebben KCWJ, van Til JA, Siesling S. Clinical decision support systems for multidisciplinary team decision-making in patients with solid cancer: Composition of an implementation model based on a scoping review. Critical Reviews in Oncology/Hematology. 2024;195:104267. https://doi.org/10.1016/j.critrevonc.2024.104267. | **Technical support system/AI** |
| Hendriks MP, Verbeek XAAM, van Manen JG, van der Heijden SE, Go SHL, Gooiker GA, et al. Clinical decision trees support systematic evaluation of multidisciplinary team recommendations. Breast Cancer Research and Treatment. 2020;183:355–63. https://doi.org/10.1007/s10549-020-05769-1. | **Technical support system/AI** |
| Henriksen DP, Ennis ZN, Panou V, Hangaard J, Jensen PB, Johansson SL, et al. Physician-led in-hospital multidisciplinary team conferences with multiple medical specialities present - A scoping review. Journal of Multimorbidity and Comorbidity. 2022;12:1–10. https://doi.org/10.1177/26335565221141745. | **Topic** |
| Heraudet L, Domblides C, Daste A, Gross-Goupil M, Ravaud A. Adaptation of multidisciplinary meeting decisions in a medical oncology department during the COVID epidemic in a less affected region of France: A prospective analysis from Bordeaux university hospital. European Journal of Cancer. 2020;135:98–100. https://doi.org/10.1016/j.ejca.2020.04.039. | **Medical outcome** |
| Hodroj K, Pellegrin D, Menard C, Bachelot T, Durand T, Toussaint P, et al. A digital solution for an advanced breast tumor board: Pilot application cocreation and implementation study. JMIR Cancer. 2023;9:e39072. https://doi.org/10.2196/39072. | **Technical support system/AI** |
| Hoeijmakers F, Heineman DJ, Daniels JM, Beck N, Tollenaar RAEM, Wouters MWJM, et al. Variation between multidisciplinary tumor boards in clinical staging and treatment recommendations for patients with locally advanced non-small cell lung cancer. Chest. 2020;158:2675–87. https://doi.org/10.1016/j.chest.2020.07.054. | **Medical outcome** |
| Hoinville L, Taylor C, Zasada M, Warner R, Pottle E, Green JSA. Improving the effectiveness of cancer multidisciplinary team meetings: Analysis of a national survey of MDT members’ opinions about streamlining patient discussions. BMJ Open Quality. 2019;8:e000631. https://doi.org/10.1136/bmjoq-2019-000631. | **Streamlining** |
| Holmes A, Kelly BD, Perera M, Eapen RS, Bolton DM, Lawrentschuk N. A systematic scoping review of multidisciplinary cancer team and decision-making in the management of men with advanced prostate cancer. World Journal of Urology. 2021;39:297–306. https://doi.org/10.1007/s00345-020-03265-1. | **Sample** |
| Honein-AbouHaidar GN, Stuart-McEwan T, Waddell T, Salvarrey A, Smylie J, Dobrow MJ, et al. How do organisational characteristics influence teamwork and service delivery in lung cancer diagnostic assessment programmes? A mixed-methods study. BMJ Open. 2017;7:e013965. | **Sample** |
| Horlait M, Baes S, Dhaene S, van Belle S, Leys M. How multidisciplinary are multidisciplinary team meetings in cancer care? An observational study in oncology departments in Flanders, Belgium. Journal of Multidisciplinary Healthcare. 2019;12:159–67. https://doi.org/10.2147/JMDH.S196660. | **Topic** |
| Horlait M, Regge M, Baes S, Eeckloo K, Leys M. Exploring non-physician care professionals’ roles in cancer multidisciplinary team meetings: A qualitative study. PloS One. 2022;17:e0263611. https://doi.org/10.1371/journal.pone.0263611. | **Profession** |
| Horlait M, van Belle S, Leys M. Input of psychosocial information during multidisciplinary team meetings at medical oncology departments: Protocol for an observational study. JMIR Research Protocols. 2018;7:e64. https://doi.org/10.2196/resprot.9239. | **Patient involvement** |
| Huo Yung Kai S, Delpierre C, Gaudin C, Goddard J, Daubisse-Marliac L, Soulié M, et al. Exhaustivité et qualité des réunions de concertation pluridisciplinaire: l’exemple des cancers prostatiques en Midi-Pyrénées. Progrès en Urologie. 2011;21:879–86. https://doi.org/10.1016/j.purol.2011.04.007. | **Language** |
| Ichikawa M, Nemoto K, Miwa M, Ohta I, Nomiya T, Yamakawa M, et al. Status of radiotherapy in a multidisciplinary cancer board. Journal of Radiation Research. 2014;55:305–8. https://doi.org/10.1093/jrr/rrt104. | **Profession** |
| Ioannidis A, Konstantinidis M, Apostolakis S, Koutserimpas C, Machairas N, Konstantinidis KM. Impact of multidisciplinary tumor boards on patients with rectal cancer. Molecular and Clinical Oncology. 2018;9:135–7. https://doi.org/10.3892/mco.2018.1658. | **Patient management without teamwork** |
| Jalil R, Lamb B, Russ S, Green JSA. The cancer multidisciplinary team from the co-ordinators perspective: Results from a national survey in the UK. BMC Health Services Research. 2012;12:457. | **Profession** |
| Jalil R, Soukup T, Akhter W, Sevdalis N, Green JSA. Quality of leadership in multidisciplinary cancer tumor boards: Development and evaluation of a leadership assessment instrument (ATLAS). World Journal of Urology. 2018;36:1031–8. https://doi.org/10.1007/s00345-018-2255-1. | **Validation** |
| Janssen A, Donnelly C, Kay J, Thiem P, Saavedra A, Pathmanathan N, et al. Developing an intranet-based lymphedema dashboard for breast cancer multidisciplinary teams: Design research study. Journal of Medical Internet Research. 2020;22:e13188. https://doi.org/10.2196/13188. | **Technical support system/AI** |
| Janssen A, Robinson T, Brunner M, Harnett P, Museth KE, Shaw T. Multidisciplinary teams and ICT: A qualitative study exploring the use of technology and its impact on multidisciplinary team meetings. BMC Health Services Research. 2018;18:444. https://doi.org/10.1186/s12913-018-3242-3. | **Technical support system/AI** |
| Keating NL, Landrum MB, Lamont EB, Bozeman SR, Shulman LN, McNeil BJ. Tumor boards and the quality of cancer care. J Natl Cancer Inst. 2013;105:113–21. https://doi.org/10.1093/jnci/djs502. | **Number of MDTs, no teamwork** |
| Kehl KL, Landrum MB, Kahn KL, Gray SW, Chen AB, Keating NL. Tumor board participation among physicians caring for patients with lung or colorectal cancer. Journal of Oncology Practice. 2015;11:e267–78. | **Patient management without teamwork** |
| Kesson EM, Allardice GM, George WD, Burns HJG, Morrison DS. Effects of multidisciplinary team working on breast cancer survival: Retrospective, comparative, interventional cohort study of 13 722 women. BMJ Clinical Research. 2012;344:e2718. https://doi.org/10.1136/bmj.e2718. | **Topic** |
| Khumalo AC, Kane BT. Perspectives on record-keeping practices in MDT meetings and meeting record utility. International Journal of Medical Informatics. 2022;161:104711. https://doi.org/10.1016/j.ijmedinf.2022.104711. | **Technical support system/AI** |
| Kim M-S, Park H-Y, Kho B-G, Park C-K, Oh I-J, Kim Y-C, et al. Artificial intelligence and lung cancer treatment decision: Agreement with recommendation of multidisciplinary tumor board. Translational Lung Cancer Research. 2020;9:507–14. https://doi.org/10.21037/tlcr.2020.04.11. | **Technical support system/AI** |
| Kim VS, Carrozzi A, Papadopoulos E, Tejero I, Thiruparanathan T, Perlis N, et al. Exploring the language used to describe older patients at multidisciplinary cancer conferences. Cancers. 2024;16:1477. https://doi.org/10.3390/cancers16081477. | **Sample** |
| Klarenbeek SE, Schuurbiers-Siebers OCJ, van den Heuvel MM, Prokop M, Tummers M. Barriers and facilitators for implementation of a computerized clinical decision support system in lung cancer multidisciplinary team meetings - A qualitative assessment. Biology. 2020;10:9. https://doi.org/10.3390/biology10010009. | **Technical support system/AI** |
| Knötgen G. Onkologische Pflege im Tumorboard: Rolle der Pflege in der multidisziplinären onkologischen Versorgung. Der Onkologe. 2020;26:991–7. https://doi.org/10.1007/s00120-020-01412-5. | **Profession** |
| Kočo L, Siebers CCN, Schlooz M, Meeuwis C, Oldenburg HSA, Prokop M, et al. The facilitators and barriers of the implementation of a clinical decision support system for breast cancer multidisciplinary team meetings - An interview study. Cancers. 2024;16:401. https://doi.org/10.3390/cancers16020401. | **Technical support system/AI** |
| Kočo L, Weekenstroo HHA, Lambregts DMJ, Sedelaar JPM, Prokop M, Fütterer JJ, et al. The effects of multidisciplinary team meetings on clinical practice for colorectal, lung, prostate and breast cancer: A systematic review. Cancers. 2021;13:4159. https://doi.org/10.3390/cancers13164159. | **Medical outcome** |
| Kreidieh F, Tfayli A. Impact of thoracic multidisciplinary tumor boards on the management of patients with cancer: A retrospective study at the American university of Beirut medical center. Molecular and Clinical Oncology. 2023;18:6. https://doi.org/10.3892/mco.2022.2602. | **Middle- and low-income countries** |
| Lamb BW, Miah S, Skolarus TA, Stewart GD, Green JSA, Sevdalis N, et al. Development and validation of a short version of the metric for the observation of decision-making in multidisciplinary tumor boards: MODe-Lite. Annals of Surgical Oncology. 2021;28:7577–88. https://doi.org/10.1245/s10434-021-09989-7. | **Validation** |
| Lamb BW, Wong HWL, Vincent C, Green JSA, Sevdalis N. Teamwork and team performance in multidisciplinary cancer teams: Development and evaluation of an observational assessment tool. BMJ Quality & Safety. 2011;20:849–56. https://doi.org/10.1136/bmjqs.2010.048660. | **Validation** |
| Lambert LK, Havaei F, Beck SM, Ma A, Larmet J, Kaur J, et al. An early evaluation of team consistency and scope optimization in team-based cancer care. BMC Cancer. 2025;25:371. https://doi.org/10.1186/s12885-025-13644-9. | **No MDT** |
| Lawaczeck L, Rüdiger A, Hennenlotter J, Hammes J, Spingler V, Walz S, et al. Impact of interdisciplinary tumor boards (ITB) and personalized treatment on survival outcomes in metastatic castration-resistant prostate cancer. Journal of Cancer Research and Clinical Oncology. 2025;151:101. https://doi.org/10.1007/s00432-025-06135-8. | **Medical outcome** |
| Layfield DM, Flashman KG, Benitez Majano S, Senapati A, Ball C, Conti JA, et al. Changing patterns of multidisciplinary team treatment, early mortality, and survival in colorectal cancer. BJS Open. 2022;6:zrac098. https://doi.org/10.1093/bjsopen/zrac098. | **Medical outcome** |
| Le Bian ZA, Costi R, Bruderer A, Hervé C, Smadja C. Multidisciplinary team meeting in digestive oncology: When opinions differ. Clinical and Translational Science. 2014;7:319–23. https://doi.org/10.1111/cts.12164. | **Topic** |
| Lee B, Kim K, Choi JY, Suh DH, No JH, Lee H-Y, et al. Efficacy of the multidisciplinary tumor board conference in gynecologic oncology: A prospective study. Medicine. 2017;96:e8089. https://doi.org/10.1097/MD.0000000000008089. | **Patient management without teamwork** |
| Leonhardt CS, Lanzenberger L, Puehringer R, Klaiber U, Hauser I, Strobel O, et al. Evidence-based cancer care: Assessing guideline adherence of multidisciplinary tumor board recommendations for breast and colorectal cancer in a non-academic medical center. Journal of Cancer Research and Clinical Oncology. 2024;151:4. https://doi.org/10.1007/s00432-024-06049-x. | **Topic** |
| Levin G, Gotlieb W, Ramirez P, Meyer R, Brezinov Y. ChatGPT in a gynaecologic oncology multidisciplinary team tumour board: A feasibility study. BJOG. 2025;132:99–101. https://doi.org/10.1111/1471-0528.17929. | **Technical support system/AI** |
| Licitra L, Keilholz U, Tahara M, Lin J-C, Chomette P, Ceruse P, et al. Evaluation of the benefit and use of multidisciplinary teams in the treatment of head and neck cancer. Oral Oncology. 2016;59:73–9. https://doi.org/10.1016/j.oraloncology.2016.06.002. | **Topic** |
| Lim RS, Kielar AZ, El-Maraghi RH, Fraser MA, Nessim C, Thipphavong S. Multidisciplinary retroperitoneal and pelvic soft-tissue sarcoma case conferences: The added value that radiologists can provide. Current Oncology. 2017;24:e171–5. https://doi.org/10.3747/co.24.3478. | **Profession** |
| Lindblad M, Jestin C, Johansson J, Edholm D, Linder G. Multidisciplinary team meetings improve survival in patients with esophageal cancer. Dis Esophagus. 2024;37. https://doi.org/10.1093/dote/doae061. | **Medical outcome** |
| List H, Kristensen DB, Graumann O. “The highest decision-making level” - Multidisciplinary team meetings as boundary spaces. Social Science & Medicine. 2025;371:117886. https://doi.org/10.1016/j.socscimed.2025.117886. | **Topic** |
| Liu A, Vicenzi P, Sharma I, Orr K, Teller C, Koentz M, et al. Molecular tumor boards: The next step towards precision therapy in cancer care. Hematology Reports. 2023;15:244–55. https://doi.org/10.3390/hematolrep15020025. | **Rare diseases** |
| Liu JC, Kaplon A, Blackman E, Miyamoto C, Savior D, Ragin C. The impact of the multidisciplinary tumor board on head and neck cancer outcomes. Laryngoscope. 2020;130:946–50. https://doi.org/10.1002/lary.28066. | **Patient management without teamwork** |
| Lohberger Timsit B, Deroux A, Bouillet L, Colombe B, Lugosi M. Évaluation des réunions de concertation pluridisciplinaire de médecine interne au CHU Grenoble Alpes. La Revue de Médecine Interne. 2021;42:452–8. https://doi.org/10.1016/j.revmed.2020.11.013. | **Language** |
| Lu J, Jiang Y, Qian M, Lv L, Ying X. The improved effects of a multidisciplinary team on the survival of breast cancer patients: Experiences from China. International Journal of Environmental Research and Public Health. 2019;17:277. https://doi.org/10.3390/ijerph17010277. | **Medical outcome** |
| Luca S, Fiori C, Tucci M, Poggio M, Allis S, Bollito E, et al. Prostate cancer management at an Italian tertiary referral center: Does multidisciplinary team meeting influence diagnostic and therapeutic decision-making process? A snapshot of the everyday clinical practice. Minerva Urologica e Nefrologica. 2019;71:576–82. https://doi.org/10.23736/S0393-2249.19.03231-4. | **Patient management without teamwork** |
| Lucarini A, Garbarino GM, Orlandi P, Garofalo E, Bragaglia L, Laracca GG, et al. From “Cure” to “Care”: The role of the MultiDisciplinary team on colorectal cancer patients’ satisfaction and oncological outcomes. Journal of Multidisciplinary Healthcare. 2022;15:1415–26. https://doi.org/10.2147/JMDH.S362550. | **Patient involvement** |
| Luijten JCHBM, Haagsman VC, Luyer MDP, Vissers PAJ, Nederend J, Huysentruyt C, et al. Implementation of a regional video multidisciplinary team meeting is associated with an improved prognosis for patients with oesophageal cancer a mixed methods approach. European Journal of Surgical Oncology. 2021;47:3088–96. https://doi.org/10.1016/j.ejso.2021.04.020. | **Medical outcome** |
| Luijten JCHBM, Vissers PAJ, Brom L, de Bièvre M, Buijsen J, Rozema T, et al. Clinical variation in the organization of clinical pathways in esophagogastric cancer, a mixed method multiple case study. BMC Health Services Research. 2022;22:527. https://doi.org/10.1186/s12913-022-07845-2. | **Medical outcome** |
| Luijten JCHBM, Vissers PAJ, Geerts J, Lemmens VEP, van Hillegersberg R, Beerepoot L, et al. Hospital practice variation in the proportion of patients with esophagogastric cancer discussed during an expert multidisciplinary team meeting. European Journal of Surgical Oncology. 2023;49:106880. https://doi.org/10.1016/j.ejso.2023.03.216. | **Topic** |
| Lukac S, Dayan D, Fink V, Leinert E, Hartkopf A, Veselinovic K, et al. Evaluating ChatGPT as an adjunct for the multidisciplinary tumor board decision-making in primary breast cancer cases. Archives of Gynecology and Obstetrics. 2023;308:1831–44. https://doi.org/10.1007/s00404-023-07130-5. | **Technical support system/AI** |
| Luu TT. Cancer patient management: Role of multidisciplinary teams. BMJ Supportive & Palliative Care. 2022;12:201–6. https://doi.org/10.1136/bmjspcare-2021-003039. | **No full text** |
| Ma H, Li H, Xu T, Gao Y, Liu S, Wang W, et al. Multidisciplinary team quality improves the survival outcomes of locally advanced rectal cancer patients: A post hoc analysis of the STELLAR trial. Radiotherapy and Oncology. 2024;200:110524. https://doi.org/10.1016/j.radonc.2024.110524. | **Patient management without teamwork** |
| Macchia G, Ferrandina G, Patarnello S, Autorino R, Masciocchi C, Pisapia V, et al. Multidisciplinary tumor board smart virtual assistant in locally advanced cervical cancer: A proof of concept. Frontiers in Oncology. 2021;11:797454. https://doi.org/10.3389/fonc.2021.797454. | **Technical support system/AI** |
| Maeng CH, Ahn HK, Oh SY, Lim S, Kim B-S, Kim DY. Practice patterns of multidisciplinary team meetings in Korean cancer care and patient satisfaction with this approach. The Korean Journal of Internal Medicine. 2020;35:205–14. https://doi.org/10.3904/kjim.2019.189. | **Patient involvement** |
| Mano MS, Çitaku FT, Barach P. Implementing multidisciplinary tumor boards in oncology: A narrative review. Future Oncology. 2022;18:375–84. https://doi.org/10.2217/fon-2021-0471. | **Topic** |
| Marshall CL, Petersen NJ, Naik AD, Vander Velde N, Artinyan A, Albo D, et al. Implementation of a regional virtual tumor board: A prospective study evaluating feasibility and provider acceptance. Telemedicine Journal and e-Health. 2014;20:705–11. https://doi.org/10.1089/tmj.2013.0320. | **Sample** |
| Mäurer I, Drescher R, Hammersen J, Dieckmann N, Gremme Y, Sturm M-J, et al. Development and implementation of a student tumor board as a teaching format for medical students. Journal of Cancer Research and Clinical Oncology. 2023;149:16087–96. https://doi.org/10.1007/s00432-023-05336-3. | **Sample** |
| Mazouni C, Deneuve J, Arnedos M, Prenois F, Saghatchian M, André F, et al. Decision-making from multidisciplinary team meetings to the bedside: Factors influencing the recruitment of breast cancer patients into clinical trials. Breast. 2014;23:170–4. https://doi.org/10.1016/j.breast.2013.12.008. | **Patient involvement** |
| Mehta SN, Shenvi EC, Blair SL, Caudle A, Lowenstein LM, Kelly KJ. Leveraging the multidisciplinary tumor board for dissemination of evidence-based recommendations on the staging and treatment of gastric cancer: A pilot study. Annals of Surgical Oncology. 2023;30:1120–9. https://doi.org/10.1245/s10434-022-12628-4. | **Medical outcome** |
| Merker L, Conroy S, El-Wakeel H, Laurence N. Streamlining the multi-disciplinary team meeting: The introduction of robust pre-preparation methods and its effect on the length of case discussions. Journal of Multidisciplinary Healthcare. 2023;16:613–22. https://doi.org/10.2147/JMDH.S387174. | **Streamlining** |
| Milana F, Famularo S, Luberto A, Rimassa L, Scorsetti M, Comito T, et al. Multidisciplinary tumor board in the management of patients with colorectal liver metastases: A single-center review of 847 patients. Cancers. 2022;14:3952. https://doi.org/10.3390/cancers14163952. | **Patient management without teamwork** |
| Mnajjed L, Krempl G. Increased efficiency of tumor board with introduction of the consensus slate. Ear, Nose & Throat Journal. 2025;:1–6. | **Technical support system/AI** |
| Morabito A, Mercadante E, Muto P, Manzo A, Palumbo G, Sforza V, et al. Improving the quality of patient care in lung cancer: Key factors for successful multidisciplinary team working. Exploration of Targeted Anti-tumor Therapy. 2024;5:260–77. https://doi.org/10.37349/etat.2024.00217. | **Sample** |
| Munro A, Brown M, Niblock P, Steele R, Carey F. Do multidisciplinary team (MDT) processes influence survival in patients with colorectal cancer? A population-based experience. BMC Cancer. 2015;15:686. https://doi.org/10.1186/s12885-015-1683-1. | **Medical outcome** |
| Munro AJ, Swartzman S. What is a virtual multidisciplinary team (vMDT)? British Journal of Cancer. 2013;108:2433–41. https://doi.org/10.1038/bjc.2013.231. | **No study** |
| Naeem A, Jacob W. Evaluating the benefits and challenges of using patient preferences as a tool for clinical decision making in oncology multidisciplinary team meetings within the national health service: A qualitative study. Oncology Research and Treatment. 2025;:1–7. https://doi.org/10.1159/000543741. | **Patient involvement** |
| Nobori A, Jumniensuk C, Chen X, Enzmann D, Dry S, Nelson S, et al. Electronic health record-integrated tumor board application to save preparation time and reduce errors. JCO Clinical Cancer Informatics. 2022;6:e2100142. | **Technical support system/AI** |
| Orlowski C, Lai J, Vereker M, Antill Y, Richardson G, White M, et al. Impact of multidisciplinary team meetings on the management of patients with breast cancer in a large private healthcare facility. Asia-Pacific Journal of Clinical Oncology. 2024;20:285–91. https://doi.org/10.1111/ajco.13947. | **Patient management without teamwork** |
| Ouattassi N, Azdad A, Debaghi H, Zarghili A, El Amine El Alami MN. “OCTOPUS”: An intelligent tool for assisted multidisciplinary ORL oncology meetings - Preliminary study. OTO Open. 2023;7:e64. https://doi.org/10.1002/oto2.64. | **Technical support system/AI** |
| Palve JS, Ylitalo LK, Luukkaala TH, Jernman JM, Korhonen NJ. A second expert pathology review of cutaneous melanoma in multidisciplinary meetings: Impact on treatment decisions. Surgical Oncology. 2019;30:72–5. https://doi.org/10.1016/j.suronc.2019.05.024. | **Profession** |
| Pang K, Guo X, Liu T, Wang L, Chen R, Zhang Z, et al. The role of a multidisciplinary team in the diagnosis and treatment of bone and soft tissue sarcomas: A single-center experience. Journal of Personalized Medicine. 2022;12:2079. https://doi.org/10.3390/jpm12122079. | **Rare diseases** |
| Pantelimon I, Stancu AM, Coniac S, Ionescu AI, Atasiei DI, Georgescu DE, et al. Local control of advanced breast cancer-debate in multidisciplinary tumor board. Journal of Clinical Medicine. 2025;14:510. https://doi.org/10.3390/jcm14020510. | **Medical outcome** |
| Park Y-E, Chae H. The fidelity of artificial intelligence to multidisciplinary tumor board recommendations for patients with gastric cancer: A retrospective study. Journal of Gastrointestinal Cancer. 2024;55:365–72. https://doi.org/10.1007/s12029-023-00967-8. | **Technical support system/AI** |
| Pasetto S, Gatenby RA, Enderling H. Bayesian framework to augment tumor board decision making. JCO Clinical Cancer Informatics. 2021;5:508–17. | **Technical support system/AI** |
| Patel A, Franko ER, Fleshman JW. Utilizing the multidisciplinary team for planning and monitoring care and quality improvement. Clinics in Colon and Rectal Surgery. 2015;28:12–20. https://doi.org/10.1055/s-0035-1545065. | **Topic** |
| Patkar V, Acosta D, Davidson T, Jones A, Fox J, Keshtgar M. Cancer multidisciplinary team meetings: Evidence, challenges, and the role of clinical decision support technology. International Journal of Breast Cancer. 2011;2011:831605. https://doi.org/10.4061/2011/831605. | **Technical support system/AI** |
| Patkar V, Acosta D, Davidson T, Jones A, Fox J, Keshtgar M. Using computerised decision support to improve compliance of cancer multidisciplinary meetings with evidence - Based guidance. BMJ Open. 2012;2:e000439. https://doi.org/10.1136/bmjopen-2011-000439. | **Technical support system/AI** |
| Pershad AR, Graetz D, Le MA, Forrest H, Gonzalez-Guzman M, Friedrich P. Multidisciplinary care meeting practices across diverse international settings. Cancer Medicine. 2024;13:e70136. https://doi.org/10.1002/cam4.70136. | **Sample** |
| Pluyter JR, Jacobs I, Langereis S, Cobben D, Williams S, Curfs J, et al. Looking through the eyes of the multidisciplinary team: The design and clinical evaluation of a decision support system for lung cancer care. Translational Lung Cancer Research. 2020;9:1422–32. https://doi.org/10.21037/tlcr-19-441. | **Technical support system/AI** |
| Powell HA, Baldwin DR. Multidisciplinary team management in thoracic oncology: More than just a concept? The European Respiratory Journal. 2014;43:1776–86. https://doi.org/10.1183/09031936.00150813. | **No study** |
| Prabhakar CN, Fong KM, Peake MD, Lam DC, Barnes DJ. The effectiveness of lung cancer MDT and the role of respiratory physicians. Respirology. 2015;20:884–8. https://doi.org/10.1111/resp.12520. | **Profession** |
| Prades J, Coll-Ortega C, Dal Lago L, Goffin K, Javor E, Lombardo C, et al. Use of information and communication technologies (ICTs) in cancer multidisciplinary team meetings: An explorative study based on EU healthcare professionals. BMJ Open. 2022;12:e051181. https://doi.org/10.1136/bmjopen-2021-051181. | **Technical support system/AI** |
| Price SJ, Joannides A, Plaha P, Afshari FT, Albanese E, Barua NU, et al. Impact of COVID-19 pandemic on surgical neuro-oncology multi-disciplinary team decision making: A national survey (COVID-CNSMDT Study). BMJ Open. 2020;10:e040898. https://doi.org/10.1136/bmjopen-2020-040898. | **Topic** |
| Punshon G, Endacott R, Aslett P, Brocksom J, Fleure L, Howdle F, et al. The experiences of specialist nurses working within the uro-oncology multidisciplinary team in the United Kingdom. Clinical Nurse Specialist. 2017;31:210–8. https://doi.org/10.1097/NUR.0000000000000308. | **Profession** |
| Pype P, Mertens F, Belche J, Duchesnes C, Kohn L, Sercu M, et al. Experiences of hospital-based multidisciplinary team meetings in oncology: An interview study among participating general practitioners. European Journal of General Practice. 2017;23:155–63. https://doi.org/10.1080/13814788.2017.1323081. | **Profession** |
| Quero G, Salvatore L, Fiorillo C, Bagalà C, Menghi R, Maria B, et al. The impact of the multidisciplinary tumor board (MDTB) on the management of pancreatic diseases in a tertiary referral center. ESMO Open. 2021;6:100010. https://doi.org/10.1016/j.esmoop.2020.100010. | **Patient management without teamwork** |
| Quero G, Sio D, Fiorillo C, Menghi R, Rosa F, Massimiani G, et al. The role of the multidisciplinary tumor board (MDTB) in the assessment of pancreatic cancer diagnosis and resectability: A tertiary referral center experience. Frontiers in Surgery. 2023;10:1119557. https://doi.org/10.3389/fsurg.2023.1119557. | **Patient management without teamwork** |
| Rajasekaran RB, Whitwell D, Cosker TDA, Gibbons CLMH, Carr A. Will virtual multidisciplinary team meetings become the norm for musculoskeletal oncology care following the COVID-19 pandemic? - Experience from a tertiary sarcoma centre. BMC Musculoskeletal Disorders. 2021;22:18. https://doi.org/10.1186/s12891-020-03925-8. | **Rare diseases** |
| Rasmussen TR, Gouliaev A, Jakobsen E, Hjorthaug K, Larsen LU, Meldgaard P, et al. Impact of multidisciplinary team discrepancies on comparative lung cancer outcome analyses and treatment equality. BMC Cancer. 2024;24:1423. https://doi.org/10.1186/s12885-024-13188-4. | **Medical outcome** |
| Reboux N, Cadieu E, Pruvost-Couvreur M, Carioub M, Kermarrecd M, Kermarrecb T, et al. Factors associated with non-presentation in a multidisciplinary team meeting for colon cancer: A matched retrospective cohort study in a French area. Clinics and Research in Hepatology and Gastroenterology. 2022;46:101950. | **Patient management without teamwork** |
| Rollet Q, Bouvier V, Moutel G, Launay L, Bignon A-L, Bouhier-Leporrier K, et al. Multidisciplinary team meetings: Are all patients presented and does it impact quality of care and survival - A registry-based study. BMC Health Services Research. 2021;21:1032. https://doi.org/10.1186/s12913-021-07022-x. | **Patient involvement** |
| Ronmark E, Hoffmann R, Skokic V, Klerk-Starmans M, Jaderling F, Vos P, et al. Effect of digital-enabled multidisciplinary therapy conferences on efficiency and quality of the decision making in prostate cancer care. BMJ Health & Care Informatics. 2022;29:e100588. https://doi.org/10.1136/bmjhci-2022-100588. | **Technical support system/AI** |
| Rosell L, Melander W, Lindahl B, Nilbert M, Malmström M. Registered nurses’ views on consideration of patient perspectives during multidisciplinary team meetings in cancer care. BMC Nursing. 2022;21:350. https://doi.org/10.1186/s12912-022-01127-2. | **Profession** |
| Rosell L, Wihl J, Hagberg O, Ohlsson B, Nilbert M. Function, information, and contributions: An evaluation of national multidisciplinary team meetings for rare cancers. Rare Tumors. 2019;11:1–9. https://doi.org/10.1177/2036361319841696. | **Rare diseases** |
| Rosell L, Wihl J, Nilbert M, Malmström M. Health professionals’ views on key enabling factors and barriers of national multidisciplinary team meetings in cancer care: A qualitative study. Journal of Multidisciplinary Healthcare. 2020;13:179–86. https://doi.org/10.2147/JMDH.S240140. | **Rare diseases** |
| Rosen MA, DiazGranados D, Dietz AS, Benishek LE, Thompson D, Pronovost PJ, et al. Teamwork in healthcare: Key discoveries enabling safer, high-quality care. The American Psychologist. 2018;73:433–50. https://doi.org/10.1037/amp0000298. | **Topic** |
| Ruiz-Casado A, Ortega MJ, Soria A, Cebolla H. Clinical audit of multidisciplinary care at a medium-sized hospital in Spain. World Journal of Surgical Oncology. 2014;12:53. | **Topic** |
| Savino G, Pagliara MM, Sammarco MG, Caputo CG, Blasi MA, Mattei R, et al. The role of multidisciplinary ocular and periocular cancers meetings in uveal melanoma management: A 2-year analysis. Cancers. 2025;17:2274. https://doi.org/10.3390/cancers17142274. | **Medical outcome** |
| Savino G, Piccinni F, Pagliara MM, Sammarco MG, Caputo CG, Moro A, et al. Multidisciplinary ocular and periocular cancers meetings: Implementation in a tertiary referral center and analysis over a 12-months period. BMC Ophthalmology. 2022;22:497. https://doi.org/10.1186/s12886-022-02694-3. | **Patient management without teamwork** |
| Savitz A, Fong B, Hochberg A, Rumore G, Chen C, Yun J, et al. Endocrine tumor board: Ten years’ experience of a multidisciplinary clinical working conference. The Permanente Journal. 2020;24:19.140. https://doi.org/10.7812/TPP/19.140. | **Sample** |
| Scarberry K, Ponsky L, Cherullo E, Larchian W, Bodner D, Cooney M, et al. Evaluating the impact of the genitourinary multidisciplinary tumour board: Should every cancer patient be discussed as standard of care? Canadian Urological Association Journal. 2018;12:E403–8. https://doi.org/10.5489/cuaj.5150. | **Patient management without teamwork** |
| Schellenberger B, Heuser C, Diekmann A, Krüger E, Schreiber L, Ansmann L, et al. Interruptions in multidisciplinary tumor conferences with patient participation: A video interaction analysis. Patient Education and Counseling. 2025;131:108550. https://doi.org/10.1016/j.pec.2024.108550. | **Patient involvement** |
| Scher KS, Tisnado DM, Rose DE, Adams JL, Ko CY, Malin JL, et al. Physician and practice characteristics influencing tumor board attendance: Results from the provider survey of the Los Angeles women’s health study. Journal of Oncology Practice. 2011;7:103–10. | **Topic** |
| Schmidl B, Hütten T, Pigorsch S, Stögbauer F, Hoch CC, Hussain T, et al. Assessing the role of advanced artificial intelligence as a tool in multidisciplinary tumor board decision-making for primary head and neck cancer cases. Frontiers in Oncology. 2024;14:1353031. https://doi.org/10.3389/fonc.2024.1353031. | **Technical support system/AI** |
| Shao J, Rodrigues M, Corter AL, Baxter NN. Multidisciplinary care of breast cancer patients: A scoping review of multidisciplinary styles, processes, and outcomes. Current Oncology. 2019;26:e385–97. https://doi.org/10.3747/co.26.4713. | **Sample** |
| Shore ND, Morgans AK, El-Haddad G, Srinivas S, Abramowitz M. Addressing challenges and controversies in the management of prostate cancer with multidisciplinary teams. Targeted Oncology. 2022;17:709–25. https://doi.org/10.1007/s11523-022-00925-7. | **Patient management without teamwork** |
| Sinha AP, Badawy K, Shifa B, Peterson Z, Attia M, Pinder S, et al. Improving breast cancer multidisciplinary meetings through streamlining with protocol-based management. BMJ Health & Care Informatics. 2024;31:e100949. https://doi.org/10.1136/bmjhci-2023-100949. | **Streamlining** |
| Somashekhar SP, Sepúlveda M-J, Puglielli S, Norden AD, Shortliffe EH, Rohit Kumar C, et al. Watson for oncology and breast cancer treatment recommendations: Agreement with an expert multidisciplinary tumor board. Annals of Oncology. 2018;29:418–23. https://doi.org/10.1093/annonc/mdx781. | **Technical support system/AI** |
| Soukup T, Hull L, Smith EL, Healey A, Bakolis I, Amiel SA, et al. Effectiveness-implementation hybrid type 2 trial evaluating two psychoeducational programmes for severe hypoglycaemia in type 1 diabetes: Implementation study protocol. BMJ Open. 2019;9:e030370. https://doi.org/10.1136/bmjopen-2019-030370. | **Topic** |
| Soukup T, Lamb BW, Arora S, Darzi A, Sevdalis N, Green JS. Successful strategies in implementing a multidisciplinary team working in the care of patients with cancer: An overview and synthesis of the available literature. Journal of Multidisciplinary Healthcare. 2018;11:49–61. https://doi.org/10.2147/JMDH.S117945. | **No systematic review and scoping review** |
| Soukup T, Lamb BW, Weigl M, Green JSA, Sevdalis N. An integrated literature review of time-on-task effects with a pragmatic framework for understanding and improving decision-making in multidisciplinary oncology team meetings. Frontiers in Psychology. 2019;10:1245. https://doi.org/10.3389/fpsyg.2019.01245. | **No systematic review and scoping review** |
| Soukup T, Morbi A, Lamb BW, Gandamihardja TAK, Hogben K, Noyes K, et al. A measure of case complexity for streamlining workflow in multidisciplinary tumor boards: Mixed methods development and early validation of the MeDiC tool. Cancer Medicine. 2020;9:5143–54. https://doi.org/10.1002/cam4.3026. | **Streamlining** |
| Soukup T, Stewart GD, Lamb BW. Defining an evidence-based strategy for streamlining cancer multidisciplinary team meetings. The Lancet Oncology. 2023;24:1061–3. https://doi.org/10.1016/s1470-2045(23)00440-0. | **Streamlining** |
| Specchia ML, Di Pilla A, Gambacorta MA, Filippella A, Beccia F, Farina S, et al. An IT platform supporting rectal cancer tumor board activities: Implementation process and impact analysis. International Journal of Environmental Research and Public Health. 2022;19:15808. https://doi.org/10.3390/ijerph192315808. | **Technical support system/AI** |
| Specchia ML, Frisicale EM, Carini E, Di Pilla A, Cappa D, Barbara A, et al. The impact of tumor board on cancer care: Evidence from an umbrella review. BMC Health Services Research. 2020;20:73. https://doi.org/10.1186/s12913-020-4930-3. | **No systematic review and scoping review** |
| Stairmand J, Signal L, Sarfati D, Jackson C, Batten L, Holdaway M, et al. Consideration of comorbidity in treatment decision making in multidisciplinary cancer team meetings: A systematic review. Annals of Oncology. 2015;26:1325–32. https://doi.org/10.1093/annonc/mdv025. | **Topic** |
| Steen MW, van Rijssen LB, Festen S, Busch OR, Groot Koerkamp B, van der Geest LG, et al. Impact of time interval between multidisciplinary team meeting and intended pancreatoduodenectomy on oncological outcomes. BJS Open. 2020;4:884–92. https://doi.org/10.1002/bjs5.50319. | **Medical outcome** |
| Stirling RG, Harrison A, Huang J, Lee V, Taverner J, Barnes H. Multidisciplinary meeting review in nonsmall cell lung cancer: A systematic review and meta-analysis. European Respiratory Review. 2024;33:230157. https://doi.org/10.1183/16000617.0157-2023. | **Medical outcome** |
| Stone E, Rankin NM, Currow D, Fong KM, Phillips JL, Shaw T. Optimizing lung cancer MDT data for maximum clinical impact - A scoping literature review. Translational Lung Cancer Research. 2020;9:1629–38. https://doi.org/10.21037/tlcr.2020.01.02. | **Medical outcome** |
| Taberna M, Gil Moncayo F, Jané-Salas E, Antonio M, Arribas L, Vilajosana E, et al. The multidisciplinary team (MDT) approach and quality of care. Frontiers in Oncology. 2020;10:85. https://doi.org/10.3389/fonc.2020.00085. | **Topic** |
| Takeda T, Takeda S, Uryu K, Ichihashi Y, Harada H, Iwase A, et al. Multidisciplinary lung cancer tumor board connecting eight general hospitals in Japan via a high-security communication line. JCO Clinical Cancer Informatics. 2019;3. | **Topic** |
| Taplin SH, Weaver S, Salas E, Chollette V, Edwards HM, Bruinooge SS, et al. Reviewing cancer care team effectiveness. Journal of Oncology Practice. 2015;11:239–46. https://doi.org/10.1200/JOP.2014.003350. | **Sample** |
| Taroeno-Hariadi KW, Herdini C, Briliant AS, Husodoputro HK, Dhamiyati W, Indrasari SR, et al. Multidisciplinary team meeting in the core of nasopharyngeal cancer management improved quality of care and survival of patients. Health Services Insights. 2023;16:1–8. https://doi.org/10.1177/11786329231204757. | **Patient management without teamwork** |
| Taylor C, Brown K, Lamb B, Harris J, Sevdalis N, Green JSA. Developing and testing TEAM (Team Evaluation and Assessment Measure), a self-assessment tool to improve cancer multidisciplinary teamwork. Annals of Surgical Oncology. 2012;19:4019–27. https://doi.org/10.1245/s10434-012-2493-1. | **Validation** |
| Taylor C, Finnegan-John J, Green JSA. “No decision about me without me” in the context of cancer multidisciplinary team meetings: A qualitative interview study. BMC Health Services Research. 2014;14:488. | **Patient involvement** |
| Taylor C, Shewbridge A, Harris J, Green JS. Benefits of multidisciplinary teamwork in the management of breast cancer. Breast Cancer (Dove Med Press). 2013;5:79–85. https://doi.org/10.2147/BCTT.S35581. | **No systematic review and scoping review** |
| Taylor J, Wright P, Rossington H, Mara J, Glover A, West N, et al. Regional multidisciplinary team intervention programme to improve colorectal cancer outcomes: study protocol for the Yorkshire Cancer Research Bowel Cancer Improvement Programme (YCR BCIP). BMJ Open. 2019;9:e030618. https://doi.org/10.1136/bmjopen-2019-030618. | **Sample** |
| Tong S-S, Chen Y-L, Cheng Y-F, Cheng C-Y, Huang C-L, Hung W-H, et al. Multidisciplinary tumour boards and surgical intervention improve overall survival in patients with Stage III non-small-cell lung cancer: a retrospective cohort study. Interdisciplinary CardioVascular and Thoracic Surgery. 2025;40:ivaf141. https://doi.org/10.1093/icvts/ivaf141. | **Medical outcome** |
| Tran TH, Boer J, Gyorki DE, Krishnasamy M. Optimising the quality of multidisciplinary team meetings: A narrative review. Cancer Medicine. 2022;11:1965–71. https://doi.org/10.1002/cam4.4432. | **No systematic review and scoping review** |
| Umihanic S, Osmanovic H, Selak N, Kopric D, Huseinbasic A, Sehic-Kozica E, et al. Evaluating the concordance between ChatGPT and multidisciplinary teams in breast cancer treatment planning: A study from Bosnia and Herzegovina. JCM. 2025;14:6460. https://doi.org/10.3390/jcm14186460. | **Technical support system/AI** |
| Ustabaşıoğlu F. A radiologist’s perspective on musculoskeletal tumors multidisciplinary team meetings. Balkan Medical Journal. 2024;41:1–3. https://doi.org/10.4274/balkanmedj.galenos.2023.2023-14122023. | **Profession** |
| van der Velden DL, van Herpen CML, van Laarhoven HWM, Smit EF, Groen HJM, Willems SM, et al. Molecular tumor boards: Current practice and future needs. Annals of Oncology. 2017;28:3070–5. https://doi.org/10.1093/annonc/mdx528. | **Rare diseases** |
| Walraven JEW, Desar IME, van Hoeven der JJM, Aben KKH, Hillegersberg van R, Rasch CRN, et al. Analysis of 105.000 patients with cancer: Have they been discussed in oncologic multidisciplinary team meetings? A nationwide population-based study in the Netherlands. European Journal of Cancer. 2019;121:85–93. https://doi.org/10.1016/j.ejca.2019.08.007. | **Sample** |
| Walraven JEW, Ripping TM, Oddens JR, van Rhijn BWG, Goossens-Laan CA, Hulshof MCCM, et al. The influence of multidisciplinary team meetings on treatment decisions in advanced bladder cancer. BJU International. 2023;131:244–52. https://doi.org/10.1111/bju.15856. | **Patient management without teamwork** |
| Walraven JEW, van der Meulen R, van der Hoeven JJM, Lemmens VEPP, Verhoeven RHA, Hesselink G, et al. Preparing tomorrow’s medical specialists for participating in oncological multidisciplinary team meetings: Perceived barriers, facilitators and training needs. BMC Medical Education. 2022;22:502. https://doi.org/10.1186/s12909-022-03570-w. | **Profession** |
| Walraven JEW, Verhoeven RHA, van der Hoeven JJM, van der Meulen R, Lemmens VEPP, Hesselink G, et al. Pros and cons of streamlining and use of computerised clinical decision support systems to future-proof oncological multidisciplinary team meetings. Frontiers in Oncology. 2023;13:1178165. https://doi.org/10.3389/fonc.2023.1178165. | **Technical support system/AI** |
| Wang H, Buljac-Samardzic M, Wang W, van Wijngaarden J, Yuan S, van de Klundert J. What do we know about teamwork in Chinese hospitals? A systematic review. Frontiers in Public Health. 2021;9:735754. https://doi.org/10.3389/fpubh.2021.735754. | **Topic** |
| Westeel V, Bourdon M, Cortot AB, Debieuvre D, Toffart A-C, Acquadro M, et al. Management of lung cancer patients’ quality of life in clinical practice: A Delphi study. ESMO Open. 2021;6:1–8. https://doi.org/10.1016/j.esmoop.2021.100239. | **Topic** |
| Wihl J, Falini V, Borg S, Stahl O, Jiborn T, Ohlsson B, et al. Implementation of the measure of case discussion complexity to guide selection of prostate cancer patients for multidisciplinary team meetings. Cancer Medicine. 2023;12:15149–58. https://doi.org/10.1002/cam4.6189. | **Streamlining** |
| Wihl J, Rosell L, Bendahl P-O, Mattos CBR, Kinhult S, Lindell G, et al. Leadership perspectives in multidisciplinary team meetings; observational assessment based on the ATLAS instrument in cancer care. Cancer Treatment and Research Communications. 2020;25:100231. https://doi.org/10.1016/j.ctarc.2020.100231. | **Profession** |
| Winters DA, Soukup T, Sevdalis N, Green JSA, Lamb BW. The cancer multidisciplinary team meeting: In need of change? History, challenges and future perspectives. BJU International. 2021;128:271–9. https://doi.org/10.1111/bju.15495. | **No study** |
| Yuan Y, Ye J, Ren Y, Dai W, Peng J, Cai S, et al. The efficiency of electronic list-based multidisciplinary team meetings in management of gastrointestinal malignancy: A single-center experience in southern China. World Journal of Surgical Oncology. 2018;16:146. https://doi.org/10.1186/s12957-018-1443-1. | **Technical support system/AI** |
| Yvonnet S. Une analyse de la prise de décision médicale lors des réunions de concertations pluridisciplinaires. Bulletin du Cancer. 2022;109:346–57. | **Language** |
| Zabaleta J, Aguinagalde B, Lopez I, Fernandez-Monge A, Lizarbe JA, Mainer M, et al. Utility of artificial intelligence for decision making in thoracic multidisciplinary tumor boards. Journal of Clinical Medicine. 2025;14:399. https://doi.org/10.3390/jcm14020399. | **Technical support system/AI** |
| Zandee WT, Merola E, Poczkaj K, Mestier L, Klümpen H-J, Geboes K, et al. Evaluation of multidisciplinary team decisions in neuroendocrine neoplasms: Impact of expert centres. European Journal of Cancer Care. 2022;31:e13639. https://doi.org/10.1111/ecc.13639. | **Rare diseases** |
| Zhang D-S, Zheng J-W, Zhang C-P, Cai Z-G, Li L-J, Liao G-Q, et al. 口腔癌合并全身系统性疾病患者的多学科协作诊疗模式专家共识. West China Journal of Stomatology. 2020;38:603–15. https://doi.org/10.7518/hxkq.2020.06.001. | **Language** |
| Zhang L, Yang J, Li J-J, Chen C-Y, Wang X-D, Xie Y, et al. Multidisciplinary tumor board is associated with improved survival in patients with hepatocellular carcinoma after liver transplantation. World J Clin Oncol. 2025;16. https://doi.org/10.5306/wjco.v16.i4.100729. | **Medical outcome** |
| Zhao S, Qi W, Chen J. Role of a multidisciplinary team in administering radiotherapy for esophageal cancer. BMC Cancer. 2020;20:974. https://doi.org/10.1186/s12885-020-07467-z. | **Medical outcome** |
